# Function-guided proximity mapping unveils electrophilic-metabolite sensing by proteins not present in their canonical locales

**DOI:** 10.1073/pnas.2120687119

**Published:** 2022-01-26

**Authors:** Yi Zhao, Pierre A. Miranda Herrera, Dalu Chang, Romain Hamelin, Marcus J. C. Long, Yimon Aye

**Affiliations:** ^a^Institute of Chemical Sciences and Engineering, School of Basic Sciences, Swiss Federal Institute of Technology Lausanne, 1015 Lausanne, Switzerland;; ^b^National Centre of Competence in Research Chemical Biology, University of Geneva, 1211 Geneva, Switzerland;; ^c^BayRay Innovation Center, Shenzhen Bay Laboratory, Shenzhen 518055, Guangdong, China;; ^d^Proteomics Core Facility, School of Life Sciences, Swiss Federal Institute of Technology Lausanne, 1015 Lausanne, Switzerland;; ^e^Department of Biochemistry, Faculty of Biology and Medicine, University of Lausanne, 1066 Epalinges, Switzerland

**Keywords:** reactive metabolites, function-guided proximity mapping, electrophile signaling

## Abstract

Enzyme-assisted posttranslational modifications (PTMs) constitute a major means of signaling across different cellular compartments. However, how nonenzymatic PTMs—despite their direct relevance to covalent drug development—impinge on cross-compartment signaling remains inaccessible as current target-identification (target-ID) technologies offer limited spatiotemporal resolution, and proximity mapping tools are also not guided by specific, biologically-relevant, ligand chemotypes. Here we establish a quantitative and direct profiling platform (Localis-rex) that ranks responsivity of compartmentalized subproteomes to nonenzymatic PTMs. In a setup that contrasts nucleus- vs. cytoplasm-specific responsivity to reactive-metabolite modification (hydroxynonenylation), ∼40% of the top-enriched protein sensors investigated respond in compartments of nonprimary origin or where the canonical activity of the protein sensor is inoperative. CDK9—a primarily nuclear-localized kinase—was hydroxynonenylated only in the cytoplasm. Site-specific CDK9 hydroxynonenylation—which we identified in untreated cells—drives its nuclear translocation, downregulating RNA-polymerase-II activity, through a mechanism distinct from that of commonly used CDK9 inhibitors. Taken together, this work documents an unmet approach to quantitatively profile and decode localized and context-specific signaling/signal-propagation programs orchestrated by reactive covalent ligands.

## Significance

The cell is a hive of information flow in which metabolite signals and proteins translocate across subcompartments. Peering into this submicroscopic world is hugely challenging. Using a strategy termed Localis-rex, we identify subcellular locale-specific sensors of a native reactive electrophilic metabolite. Surprisingly, several proteins sense electrophiles in locales where they do not canonically reside. One example is the nuclear protein, CDK9. In the cytosol, the electrophilic-metabolite–modified CDK9 has no negative function. However, following nuclear translocation, the electrophilic-metabolite–modified CDK9 downregulates the transcriptional activator, RNA-Polymerase-II. This exquisitely-nuanced signaling modality highlights the need to assign means, motive, and opportunity to proteins involved in reactive metabolite signaling.

Reactive metabolite sensing is emerging as a key regulatory element (1–3). In this paradigm, specific sensor proteins, or privileged first responders, are modulated by native electrophilic metabolite signals largely without enzyme assistance (2, 4). These individual protein-specific substoichiometric electrophile-labeling events may drive either gain-of-function or dominant loss-of-function signaling. Privileged sensing can arise due to a protein having enhanced association kinetics with a specific electrophile and/or through a specific electrophile-modified protein manifesting phenotypically dominant outputs. However, both characteristics are likely to contribute. Despite their importance, we remain unable to predict these first responders a priori, and we cannot explain why a known first responder shows such behavior (5).

Several ingenious methods have arisen to profile proteome ligandability, including the susceptibility of a specific cysteine to labeling by a specific electrophile. In the case of cysteine–electrophile engagement, these methods include competitive activity-based protein profiling (6–8) and direct capture of reactive electrophile species (RES)-modified targets following bulk electrophile exposure (4, 9). These bolus approaches have revolutionized the speed and scale of mining potential first responders, particularly those that can obtain high ligand occupancy under the conditions deployed. However, emerging data hint that substoichiometric covalent ligand occupancy, particularly among the fastest-reacting electrophile-sensor proteins, triggers electrophile-sensitive signaling nodes (5). We have begun to successfully exploit such substoichiometric ligand-occupancy–driven biological behaviors to covalent drug development targeting endogenous kinase isoforms through dominant-negative inhibition (10–12). Despite burgeoning biomedical significance, it is likely that occupancy thresholds (13) for triggering signaling changes are inherently below the limits of many electrophile-sensor–profiling methods to be confidently scored as a hit. Interestingly, current strategies provide limited insight into how discrete cellular microenvironments affect sensing properties, in terms of both how electrophile sensing varies with subcellular locale and how compartment-specific electrophile sensing coincides with canonical protein activity. Thus, defining the permissiveness of different locales to electrophile signaling and precise consequences of locale-specific reactive-ligand–driven events remains a formidable challenge. These limitations are of significant medicinal relevance given that protein mislocalization is linked to disease, with some proteins manifesting vacillatory tumor suppressor vs. promoter behavior as a function of altered subcellular residence (14, 15). The preponderance of evidence points to intriguing heterogeneity across compartmentalized stress responses (16). Whether these differences are due to locale-specific fluctuations in sensor-protein abundance, or compartment-specific variations in physicochemical environments, or other unidentified factors, remains unknown.

Given the growing number of covalent drugs harboring cysteine-responsive electrophilic motifs of structure/function similar to native lipid-derived electrophilic metabolites, e.g., hydroxynonenal (11, 17–19), it is likely that sensors conditioned to react rapidly with native electrophiles are also good candidates for electrophilic drug design. Here we develop a general approach to mine context-specific first responders and explore how compartmentalized nonenzymatic posttranslational modifications (PTMs) impinge on signal rewiring. We demonstrate that this approach can identify compartment-specific sensors, even proteins sensing electrophiles in a locale where they do not reside canonically. We focus on one of these proteins, the key driver of transcription, CDK9, that is a nuclear-active protein, but senses endogenous amounts of electrophiles in the cytoplasm. We identify molecular regulation underlying cytoplasmic-specific electrophile sensing and sequential inhibitory events that affect CDK9 activity in the nucleus. We further document that a modest covalent labeling of cytosolic CDK9 by the reactive metabolite hydroxynonenal can decrease the levels of several short-lived transcripts.

## Results

### Discovery of Locale-Specific Sensors.

**Fig. 1.**
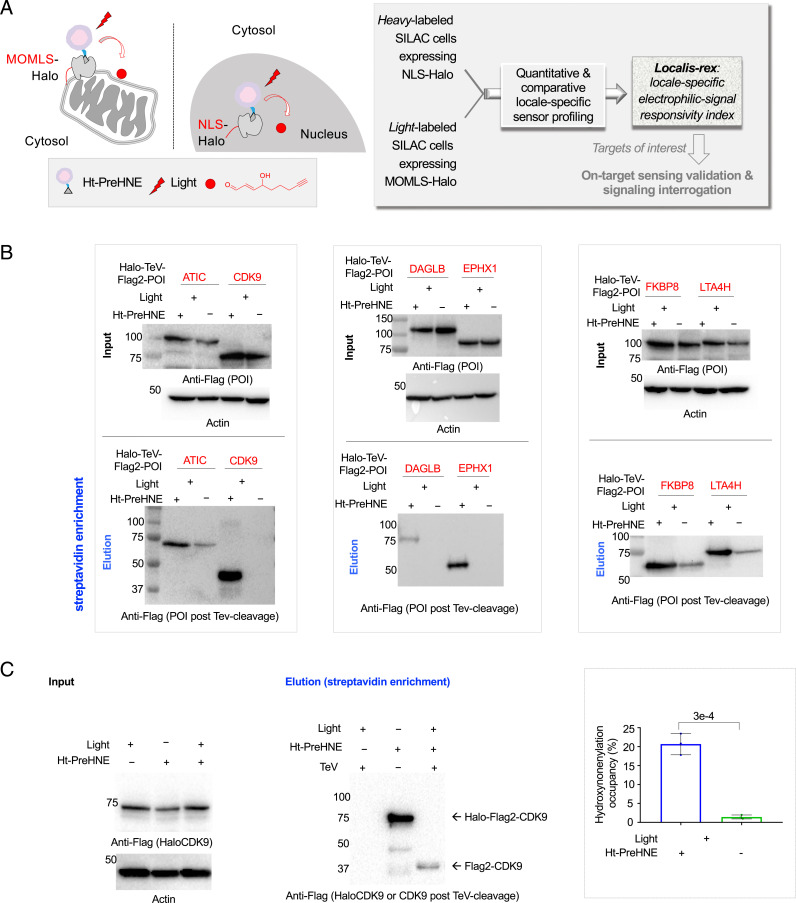
Quantitative and comparative indexing of locale-specific native first responders. Also see *SI Appendix*, Figs. S1–S13. (*A*) Localis-rex setup for indicated subcellular locales. (*Left*) Mitochondrial outer membrane (MOM) vs. nuclear-targeted spatiotemporally controlled as well as dosage-controlled release of electrophilic signals, exemplified by alkyne-functionalized hydroxynonenal (HNE) (red dot). MOMLS, MOM-localization sequence. NLS, nuclear-localization sequence. Ht-PreHNE, Halo-targetable photocaged HNE(alkyne). (*Right*, *Inset*) A representative Localis-rex strategy (*SI Appendix*, Fig. S1*A*), coupled with the established target- and signal-specific pathway-interrogation method, T-REX (10, 23) (*SI Appendix*, Fig. S1*B*). (*B*) Validation of candidate first responders by T-REX (10, 23) in HEK293T cells expressing the respective Halo-TeV-Flag2-POI (POI, protein of interest; “TeV” designates a linker housing a TeV-protease-cleavable site, here and elsewhere) by Click-biotin pulldown assay followed by Western blot analysis using indicated antibodies. Cell lysates were subjected to Click coupling (with biotin-azide), protein precipitation, resolubilization, streptavidin enrichment, washing, and elution, followed by Western blot analysis. (*C*) Determination of fractional ligand occupancy on CDK9 following T-REX (10, 23, 49) in HEK293T cells expressing Halo-TeV-Flag2-CDK9 (against controls: light alone, Ht-PreHNE-probe alone; see *SI Appendix*, Fig. S1 *B*, *Inset*). (*Left*) Representative data from Click-biotin pulldown assay (49) followed by Western blot analysis using indicated antibodies. (*Right*, *Inset*) Quantitation. Hydroxynonenal occupancy was calculated as the ratio of [“Flag2-CDK9” precipitated post–TeV-mediated separation of Halo and CDK9 in T-REX–treated samples]:[“Halo-TeV-Flag2-CDK9” precipitated from light-protected and non–TeV-treated samples], which is equivalent to (hydroxynonenylated-CDK/total CDK9). (*n* = 3 biological replicates; error bars, SD; two-tailed *t* test was applied.)

Toward the goal of discovering locale-specific sensors, HEK293T cells grown in heavy- and light-labeled stable isotope labeling with amino acids in cell culture (SILAC) (20) media and respectively expressing nuclear localization sequence (NLS)-tagged Halo or mitochondrial outer membrane localization sequence (MOMLS)-tagged Halo (Fig. 1*A* and *SI Appendix*, Fig. S1 *A* and *B*) were treated with Halo-targetable (21) photocaged hydroxynonenal (10, 22–25). MOMLS was chosen as mitochondrial outer membrane is an endogenous point source of hydroxynonenal generation from mitochondria-specific phospholipid cardiolipin oxidation (2, 26, 27). On the other hand, failure to defend excessive nuclear proteome alkylation by reactive electrophilic lipids, following lipid peroxidation of nuclear membrane rich in polyunsaturated fatty acids, plays a role in diseases such as nonalcoholic fatty liver disease (28). However, the role of specific nuclear proteins in redox-dependent stress signaling remains poorly understood (2), warranting further investigations. Correct localization of probe-bound Halo was first validated by imaging (*SI Appendix*, Fig. S1*C*). Following removal of excess unbound photocaged probe by rinsing, light illumination (*t*_1/2 of photouncaging_ < 1 min) (24, 29) liberates the electrophile in a space-, time-, and dosage-controlled manner (Fig. 1 *A*, *Inset* and *SI Appendix*, Fig. S1*A*). At both locales, Halo was expressed and functioned similarly (*SI Appendix*, Fig. S1 *D–F*). Postcell lysis, Click coupling to biotin-azide and streptavidin enrichment, native sensors hydroxynonenylated upon electrophile release in the specific locales were discernable in gels and biotin blots of eluates (*SI Appendix*, Fig. S2 *A* and *B*). First-responder proteins enriched when hydroxynonenal was released in each locale were identified using digest liquid chromatography–tandem mass spectrometry (LC-MS/MS). Given that the half-life of hydroxynonenal in cells is short (5), that we intend to catch the most responsive proteins, and that at least some hydroxynonenylated proteins may be rapidly degraded (30, 31), cells were rapidly harvested postelectrophile release, with all subsequent processing steps performed at 4 °C.

Since native electrophile signaling in vivo proceeds as a diffusion-limited second-order reaction between a specific electrophile and a protein of interest (POI) (2), our approach gives a very clear indication of POIs prone to engage electrophiles generated in a specific locale. Such locale-specific events could occur due to inherent localization of the POI to that specific locale or due to the POI being intrinsically reactive only in the identified locale.

On average from three SILAC replicates (*SI Appendix*, Fig. S3 *A* and *B* and Dataset S1), ∼62% of the proteins enriched (> +1σ or < −1σ, depending on individual replicate) upon hydroxynonenal liberation from NLS-Halo were nuclear localized; and ∼93% of the proteins enriched (> +1σ or < −1σ, depending on individual replicate) upon hydroxynonenal liberation from MOMLS-Halo were canonically cytoplasmic or mitochondrial localized. This divergence in proteins profiled matching the locale of electrophile released strongly indicates that the locale-restricted hydroxynonenal liberation faithfully reports on locale-specific sensors. Roughly 500 to 600 proteins fell into the region outside of these confidence intervals; possibly these “unenriched” proteins bind nonspecifically to the resin/biotin-azide or sense hydroxynonenal similarly in both compartments. Several well-known hydroxynonenal-responsive proteins, e.g., Keap1 (23, 25) and Pin1 (32) (both primarily cytoplasm localized), were identified within this unenriched region.

A recent report identified six locale-independent hydroxynonenal-sensitive proteins from ∼2,000 cysteines profiled (33), indicating that there are relatively few hydroxynonenal sensors. Given this precedent and because our setup liberates low hydroxynonenal levels (1 to 5 μM) (5, 22) in defined locales, no protein was similarly enriched in every run over triplicate runs (*SI Appendix*, Fig. S3*C*). Nevertheless, nine proteins were enriched in the same locale in two independent runs, but were absent in the third run (*SI Appendix*, Fig. S3*D*, *Left*, group 1). Additionally, 12 proteins were enriched in two of three runs, but were not enriched in either fraction in one run (*SI Appendix*, Fig. S3*D*, *Left*, group 2). Eleven proteins showed enrichment in two runs, but were enriched in the opposite locale in the third run (*SI Appendix*, Fig. S3*D*, *Left*, group 3). Several of these compartment-specific first responders were not enzymes. More interestingly, in ∼40% of these instances (*SI Appendix*, Fig. S4), the compartment where the sensor operated as a first responder was not its primary residence (*SI Appendix*, Figs. S3*D* and S4). Such context-specific first responders are not/have not been identified using bolus electrophile-flooding procedures. We term this unique strategy “Localis-rex,” to assess the locale-specific electrophilic-signal responsivity index. Surprisingly, in group 1 (*SI Appendix*, Fig. S3*D*, *Left*), 30% of the proteins were previously unrecognized electrophile sensors, and others (e.g., IPO7) had not been directly implicated as hydroxynonenal sensors (*SI Appendix*, Fig. S3*D*, *Right*).

### Locale-Specific Sensors Are First Responders to Hydroxynonenal.

We next used T-REX—an established individual protein-specific electrophile-sensing validation and signaling interrogation tool (10, 23–25)—to validate that the newly discovered POIs are hydroxynonenal sensors under electrophile-limited conditions (*SI Appendix*, Fig. S1*B* and Table S1). We focused on unknown sensors from group 1 (*SI Appendix*, Fig. S3 *D*, *Left*) and proteins available in the Halo-ORF library (wherein Halo fusions are validated to localize to the expected subcellular locale) (*SI Appendix*, Fig. S3 *D*, *Left*). In all, we investigated 11 proteins (*SI Appendix*, Fig. S4). We first screened the Halo-POI expression of these 11 proteins and found an ∼10-fold range of expression levels (*SI Appendix*, Fig. S5 *A* and *B*). A similar outcome was observed when the amount of active Halo within each fusion protein was assayed by the addition of “TMR-Cl” (Halo-targetable small-molecule fluorophore) (21) to lysates of cells expressing individual Halo-POI (*SI Appendix*, Fig. S5*C*). Greater than 90% loss of TMR labeling occurred when cells were pretreated with Halo-targetable photocaged hydroxynonenal (Ht-PreHNE) prior to lysis and TMR-Cl treatment (*SI Appendix*, Fig. S5*A*). We also assessed the subcellular localization of each Halo-POI against its canonical residence and also against where it sensed hydroxynonenal by Localis-rex (*SI Appendix*, Fig. S6 *A* and *B*). Halo-ATP6V1A did not localize to the expected locale (*SI Appendix*, Figs. S4 and S6 *A* and *B*) and was thus not examined further. Furthermore, Halo-NAPA showed two distinct bands post sodium dodecyl sulphate-polyacrylamide gel electrophoresis (SDS-PAGE) and overall low expression (*SI Appendix*, Fig. S5*A*) and was not analyzed further.

For POIs manifesting expression levels no more than approximately fourfold lower than LCMT1 (the most abundantly expressed protein; *SI Appendix*, Fig. S5*B*), we assayed hydroxynonenal-sensing efficiency (φ) using in-gel fluorescence analysis, following T-REX, cell lysis, TeV-protease cleavage, and Click coupling to Cy5-azide (*SI Appendix*, Fig. S7 *A–C*). Gel/blot-based methods to quantify 1) φ (also known as electrophile delivery efficiency) and 2) ligand occupancy, following T-REX–assisted precision electrophile buildup proximal to specific Halo-POI, have been previously reported (23, 29). Applying this approach to in-gel fluorescence data, ATIC, EPHX1, FKBP8, GGCT, LCMT1, LTA4H, and RCC1 emerged to be first responders to hydroxynonenal (φ = 10, 5, 16, 8, 19, 43, and 30%, respectively; *SI Appendix*, Figs. S4 and S7). Intriguingly, LTA4H was as efficient as the best hydroxynonenal sensor from all our previous studies (Keap1, φ = 20 to 40%). For POIs of expression level too low to be detectable by in-gel fluorescence analysis, namely DAGLB and CDK9, Click-biotin–streptavidin pulldown was used to evaluate electrophile sensitivity (Fig. 1 *B* and *C*). Using this alternative readout, we further confirmed four other sensors validated above: ATIC, EPHX1, FKBP8, and LTA4H (Fig. 1*B*). The Click-biotin pulldown assay in this case does not provide φ directly, but only ligand occupancy (29). However, because ligand occupancy is directly linked to φ^29^ (see equation in *SI Appendix*, Fig. S4 legend) and because photouncaging efficiency of our photocaged electrophile on Halo-CDK9 was quantifiable from in-gel fluorescence analysis in *SI Appendix*, Fig. S7*A*, we derived φ_CDK9_ to be 23%. These results attest to the ability of Localis-rex to identify electrophile sensors.

There was no correlation between Halo-POI expression and φ (*SI Appendix*, Fig. S7*C*), indicating that φ is an absolute measure of the POI’s electrophile-responsivity index. However, the proteins in this plot were identified in our Localis-rex datasets, raising the possibility that φs calculated from the ensemble of cytoplasm:nucleus-localized protein may not reflect the true electrophile-sensing capacity of the POI. Tools to study protein-specific electrophile sensing and signaling across different compartments have not yet been disclosed. We thus chose to test this posit on the six proteins described below. This set was chosen to allow us to assess locale-specific sensing in a set of proteins covering a wide range of expression levels (∼10-fold difference across) and φs (∼8-fold) (*SI Appendix*, Fig. S7*C*); functions, including transcription, nuclear translocation, axonal growth, etc.; and electrophile responsivity in locales in which these proteins do not canonically reside.

### Locale-Specific Sensing Predicted by Localis-rex is Recapitulated by Both Locale-Specific T-REX and Whole-Cell Hydroxynonenal Treatment.

**Fig. 2.**
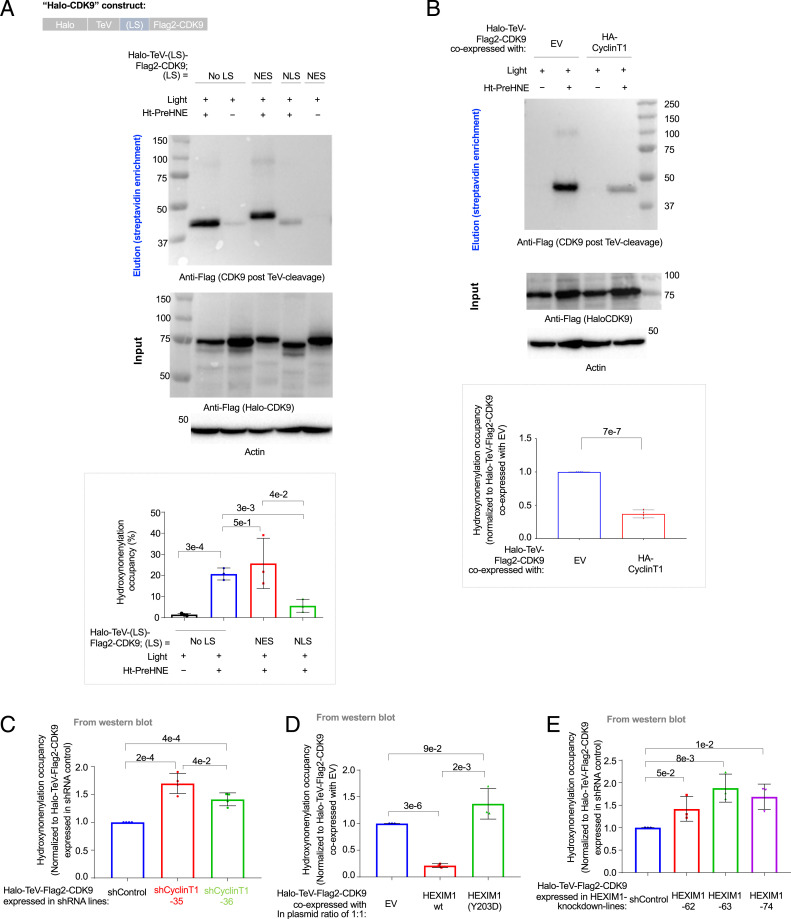
CDK9 is a context-specific electrophile sensor; CyclinT1 and HEXIM1 both antagonize CDK9 hydroxynonenylation (*SI Appendix*, Figs. S8 *A* and *B* and S14–S19). (*A*) HEK293T cells ectopically expressing Halo-TeV-(LS)-Flag2-CDK9 (where “LS” designates a specific localization sequence tagged and “No LS” means no-locale restricted) were assayed for extent of hydroxynonenal modification post Click-biotin pulldown. *(Top)* Schematic of CDK9 constructs. *(Middle)* Representative blots for Click-biotin pulldown assay, including both elution and input, showing anti-Flag [Halo-TeV-(LS)-Flag2-CDK9] and anti-actin blots. *(Bottom*, *Inset*) Quantitation (*n* = 3 biological replicates, error bars indicate SD; two-tailed *t* test was applied). See also *SI Appendix*, Figs. S8 *A* and *B*, S14*A*, and S17*A*. [Note: In all subsequent figures, Halo-TeV-Flag2-CDK9 denotes the “No LS” version; where necessary, Halo-TeV-(LS)-Flag2-CDK9 denotes constructs bearing a specific LS.] (*B*) T-REX (and corresponding controls) was performed in HEK293T cells transfected with a plasmid expressing Halo-TeV-Flag2-CDK9 together with either an empty vector (EV) or a plasmid expressing HA-CyclinT1. The extent of CDK9 hydroxynonenylation was assessed using a Click-biotin pulldown assay. *(Top)* Elution (anti-Flag, for Flag2-CDK9 post-TeV cleavage). *(Middle)* Input (anti-Flag, for full-length Halo-TeV-Flag2-CDK9 and anti-actin). *(Bottom*, *Inset*) Quantitation (*n* = 4 biological replicates, error bars indicate SD; two-tailed *t* test was applied) (*SI Appendix*, Fig. S17*C*). (*C*) HEK293T cells were infected with lentivirus delivering a single CyclinT1-specific short hairpin RNA (shRNA) or a nontargeting shRNA (shControl) (35 and 36 indicate two independent knockdown lines expressing different CyclinT1-specific shRNAs). Relative hydroxynonenal occupancy on CDK9 was evaluated by Click-biotin pulldown assay and Western blot analysis, following T-REX in each sh(CyclinT1) line [or sh(Control) line] expressing Halo-TeV-Flag2-CDK9. Quantitation: (*n* = 4 biological replicates, error bars indicate SD; two-tailed *t* test was applied) (*SI Appendix*, Figs. S17 *D* and *E* and S18*A*). (*D*) HEK293T cells were transfected with a plasmid encoding Halo-TeV-Flag2-CDK9 and either empty vector (EV) or another plasmid expressing HA-HEXIM1(WT) or CDK9-binding-defective mutant, HEXIM1(Y203D), in a plasmid ratio of 1:1. Following T-REX, cells were lysed, and the extent of hydroxynonenylation on CDK9 was evaluated by Click-biotin pulldown assay and Western blot analysis. Quantitation: (*n* = 3 biological replicates, error bars indicate SD; two-tailed *t* test was applied) (*SI Appendix*, Fig. S19*A*). (*E*) Similar experiment to *D*, but lines expressing different specific shRNAs, each targeting HEXIM1, were used (*n* = 3 biological replicates, error bars indicate SD; two-tailed *t* test was applied) (*SI Appendix*, Fig. S19 *B* and *C*).

To probe locale-specific electrophile responsivity, we envisioned that T-REX (*SI Appendix*, Fig. S1*B*) could be replicated on Halo-POI constructs bearing different localization tags (NLS or nuclear exclusion sequence [NES]). Focusing first on the pool of proteins predicted to sense in the cytoplasm (CDK9, DAGLB, GGCT, LCMT1, and RCC1), all these proteins, when NLS or NES tagged, showed, respectively, strong nuclear and cytoplasmic localization (*SI Appendix*, Fig. S8 *A–C*). Following T-REX, all five proteins showed significantly increased sensing when expressed with an NES tag than with an NLS tag (Fig. 2*A* and *SI Appendix*, Figs. S9*A*, S10*A*, S11*A*, and S12*A*). This observation applied even to CDK9 whose canonical function is in the nucleus. Conversely, for EPHX1, which also showed expected localization in the NLS and NES fusions (*SI Appendix*, Fig. S13*A*), Localis-rex predicted better sensing in the nucleus than in the cytoplasm. NLS-EPHX1 was a significantly better electrophile sensor than NES-EPHX1 (*SI Appendix*, Fig. S13*B*). As we go on to demonstrate further below, this result indicates that electrophile sensing can occur in either the nucleus or the cytoplasm, but specific proteins have specific regions in which they sense preferentially.

We further assessed this locale-specific sensing ability in the context of bolus hydroxynonenal treatment (2 h, 15 µM), which is the go-to method in the field to mimic global electrophilic stress. Furthermore, this approach constitutes a stringent test for targets profiled by Localis-rex because hydroxynonenal available in the cell following bolus treatment is unconstrained, prolonged, and in high excess. Thus, observing selective labeling on a POI is strongly disfavored by this bolus approach (especially under low-dose/limited-time exposure).

The outcomes (*SI Appendix*, Figs. S9*B*, S10*B*, S11*B*, S12*B*, S13*C*, S14 *A* and *B*, and S15 *A–C*) largely agreed with data derived from T-REX above; in only one instance (LCMT1)—one of our most highly expressed proteins that also had the most potent sensing ability—was there no difference in sensing under bolus conditions between the NLS and NES constructs (*SI Appendix*, Fig. S11*B*). Notably, for four of the six proteins, a more pronounced difference in locale-specific sensing was observed following T-REX than under bolus conditions (*SI Appendix*, Fig. S15 *A* and *B*). This discrepancy is likely due to mass action effects and possibly overalkylation that can happen during whole-cell administration of reactive electrophiles. Consistent with this notion, plotting fold difference between NES and NLS sensing (under bolus conditions) against protein expression level fitted to a line with a significantly negative gradient (*SI Appendix*, Fig. S15 *C*, *Left*). When the same analysis was performed for T-REX–derived data, there was no deviation from 0 (*SI Appendix*, Fig. S15 *C*, *Right*). All proteins also showed ability to undergo nuclear–cytoplasmic translocation to variable degrees (*SI Appendix*, Fig. S15*D*). Thus, the locale specificity inferred under bolus conditions could also have been affected by translocation during the assay.

### CDK9-Specific Sensing Is CyclinT1 and HEXIM1 Dependent.

To study signaling ramifications of locale-specific electrophile sensing, we elected to investigate CDK9 further particularly because its canonical function occurs in the nucleus, whereas Localis-rex profiling and subsequent validations altogether pointed to cytoplasm-specific electrophile sensing (Fig. 2*A* and *SI Appendix*, Figs. S4, S8 *A* and *B*, and S14 *A* and *B*). Furthermore, CDK9 is a regulator of transcription, a process not commonly associated with electrophile sensing.

We first confirmed that HaloTag fusion does not significantly affect CDK9 cellular distribution. We directly compared nuclear:cytoplasmic distribution of Flag-CDK9 and Halo-CDK9. Both constructs showed ∼0.7:1 nuclear:cytoplasmic content by immunofluorescence (*SI Appendix*, Fig. S8 *A* and *B*), consistent with several literature reports (34–37). Furthermore, the nuclear import rates of Halo-CDK9 and Flag-CDK9 were identical, following treatment with Leptomycin-B, a nuclear-export blocker (*SI Appendix*, Fig. S16). Also consistent with previous data (38), we discovered that the total expression of CDK9 is self-regulated (i.e., combined levels of endogenous CDK9 and Halo-CDK9 in Halo-CDK9–transfected cells were similar to the levels of endogenous CDK9 in empty-vector [EV]-transfected cells [*SI Appendix*, Fig. S14*A*]), thereby minimizing potential POI-overexpression–associated artifacts.

Upon transfection of Halo-CDK9, the phosphorylation of a direct downstream nuclear target of CDK9, RNA-polymerase-II (RNAPII) (39), remained unaltered (*SI Appendix*, Fig. S17*A*). We refer to this CDK9-promoted RNAPII activity as phosphorylation at the RNAPII C-terminal domain (CTD) “serine-2” site (“*Ser2*” hereafter). Conversely, in CDK9-knockdown lines (∼60% knockdown) (*SI Appendix*, Table S2), RNAPII CTD phospho-*Ser2* was downregulated (*SI Appendix*, Fig. S17*B*), consistent with previous data (40). Since the loss of endogenous CDK9 upon ectopic expression of Halo-CDK9 (*SI Appendix*, Fig. S14*A*) is similar in magnitude to CDK9 depletion in CDK9-knockdown cells (*SI Appendix*, Fig. S17*B*), which themselves undergo a reduction in RNAPII CTD phospho-*Ser2* (*SI Appendix*, Fig. S17*B*), we conclude that ectopic Halo-CDK9 is approximately as active as wild-type CDK9. In other words, the ectopic Halo-CDK9 complements loss of endogenous CDK9 incurred upon Halo-CDK9 ectopic expression (which would otherwise lead to reduction in phosphorylation of a key downstream target), allowing us to conclude that Halo-CDK9 is kinase active.

### CDK9-Specific Sensing Is CyclinT1 and HEXIM1 Dependent.

We hypothesized that a binding partner of CDK9 in the nucleus may preclude CDK9 hydroxynonenylation. In the nucleus, CDK9 associates with CyclinT1, to assemble the P-TEFb complex that regulates RNAPII activity (39). Specifically, P-TEFb–catalyzed CTD *Ser2* phosphorylation is a checkpoint to transition RNAPII to productive elongation (39). Although other proteins have been proposed to promote *Ser2* phosphorylation, based on in vitro experiments (41), the principal player in *Ser2* phosphorylation in cells is agreed to be CDK9 (42). Since CyclinT1 expression in some cell lines elevates the nuclear content of CDK9, we tested whether CyclinT1 inhibits CDK9 hydroxynonenal sensing. CyclinT1 overexpression increased the nuclear proportion of CDK9 (*SI Appendix*, Fig. S17*C*). We also observed a decrease in the extent of CDK9 hydroxynonenylation in CyclinT1-overexpressing lines (Fig. 2*B*). Conversely, CyclinT1 knockdown increased CDK9 hydroxynonenylation (Fig. 2*C*) in a manner dependent on knockdown efficiency (*SI Appendix*, Fig. S17*D* and Tables S2 and S3), although the cytoplasmic pool of CDK9 also increased upon CyclinT1 knockdown (*SI Appendix*, Fig. S17*E*). A similar increase in hydroxynonenylation was also observed for endogenous CDK9 in CyclinT1-knockdown lines, following whole-cell hydroxynonenal treatment (*SI Appendix*, Fig. S17*F*). The enriched band in this case was not detected in CDK9-knockdown lines (*SI Appendix*, Fig. S17 *F*, *Inset*, last three bars), validating our detection strategy.

We next uncoupled the effects of nuclear accumulation and hydroxynonenal sensing that accompany changes in CyclinT1 expression. Following T-REX in CyclinT1-knockdown cells expressing Halo-NLS-CDK9, we examined how CyclinT1 deficiency affected sensing by nuclear-restricted CDK9. Consistent with CyclinT1 competitively affecting CDK9 hydroxynonenylation, enhanced hydroxynonenylation of nuclear-restricted CDK9 was observed in CyclinT1-deficient cells (*SI Appendix*, Fig. S18*A*). Indeed, in CyclinT1-knockdown lines, φ values were similar between nuclear-targeted Halo-CDK9 and the untargeted construct [e.g., φ (in shCyclinT1 line 36) = 26% and 33%, respectively] (Fig. 2*C* and *SI Appendix*, Fig. S18*A*). When CyclinT1-knockdown cells expressing nuclear-restricted CDK9 were treated directly with hydroxynonenal (*SI Appendix*, Fig. S18*B*), hydroxynonenylation was also observed.

We further investigated how HEXIM1—a nuclear protein that negatively regulates the P-TEFb complex (39)—affects CDK9 hydroxynonenylation. HEXIM1 overexpression significantly reduced CDK9 hydroxynonenylation (Fig. 2*D* and *SI Appendix*, Fig. S19*A*). HEXIM1(Y203D)—which cannot bind CDK9 (43, 44)—overexpression did not suppress CDK9 hydroxynonenylation. Conversely, HEXIM1 depletion elevated CDK9 hydroxynonenylation (Fig. 2*E* and *SI Appendix*, Fig. S19 *B* and *C*). Thus, for CDK9, changes in locale usher in reorganization of CDK9 association in terms of HEXIM1 and CyclinT1, which modulate hydroxynonenal sensing. Overall these data on CDK9—and results from EPHX1 above—both indicate that the nucleus is not a refractory environment to electrophile signaling, but the interactome and other protein-intrinsic aspects are responsible for locale-specific sensing.

### Site-Specific CDK9 Hydroxynonenylation Occurs on C95.

**Fig. 3.**
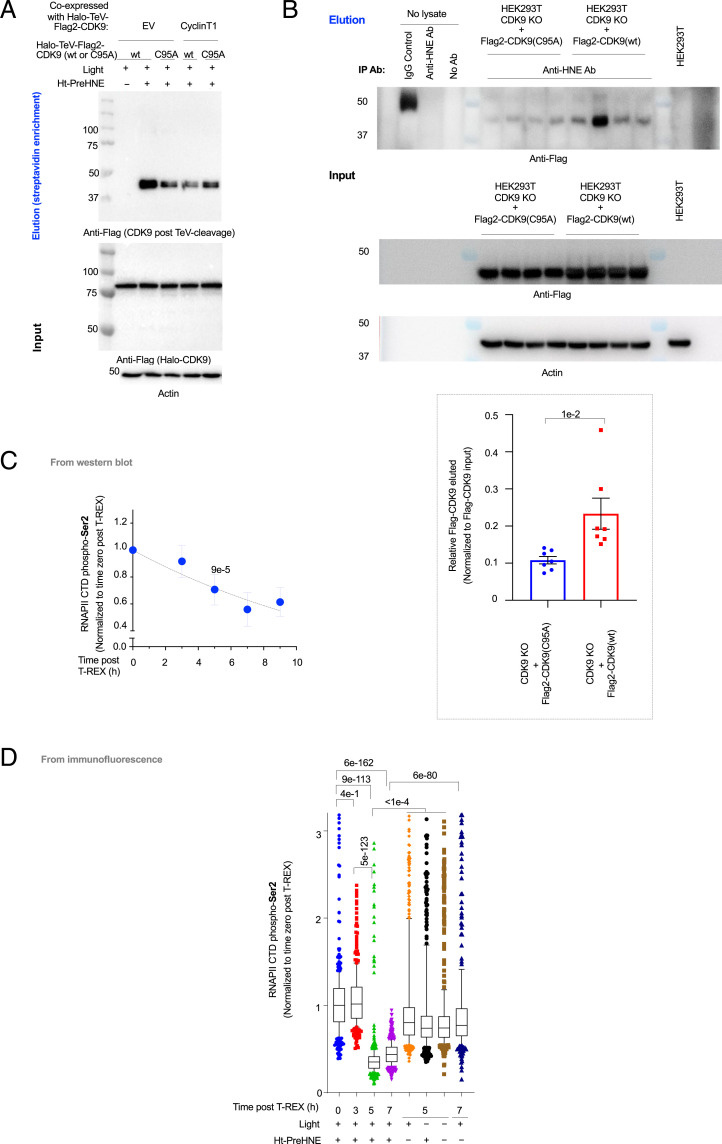
CDK9 functional hydroxynonenal sensing at C95 downregulates CDK9 kinase activity, inhibiting RNAPII CTD phosphorylation at *Ser2*. Also see *SI Appendix*, Figs. S14–S21. (*A*) HEK293T cells were transfected with the indicated pairs of constructs (EV denotes empty vector) and subjected to either light alone or T-REX. Representative Western blot following Click-biotin pulldown is shown (*SI Appendix*, Fig. S20 *A* and *B*). (*B*) Immunoprecipitation of hydroxynonenylated CDK9 using anti-hydroxynonenal antibody (anti-HNE Ab)-treated protein-G resin that was incubated with corresponding cell lysate originating from CDK9 KO HEK293T cells rescued with either Flag2-CDK9(WT) or (C95A). The elution (*Top*) and input (*Bottom*) samples were subjected to Western blot analysis. (*Inset*) Quantitation (*n* = 7 biological replicates for each condition. Error bars indicate SEM; two-tailed *t* test was applied) (*SI Appendix*, Fig. S20 *C–E*). (*C*) HEK293T cells expressing Halo-TeV-Flag2-CDK9 were subjected to T-REX and harvested at the indicated times post–CDK9-specific hydroxynonenylation (i.e., postlight exposure), and whole-cell lysates were analyzed by Western blot using anti-RNAPII CTD phospho-*Ser2* antibodies. Quantitation is shown and see *SI Appendix*, Fig. S21*A* for representative data. CTD denotes C-terminal domain (*n* = 3, error bars indicate SD; two-tailed *t* test was applied). *P* value at 5-h timepoint results from two-tailed *t* test performed against time 0. A 5-h timepoint was used in several functional assays discussed elsewhere. (*D*) A similar experiment to *C* but cells were fixed at the indicated times post–CDK9-specific hydroxynonenylation, and IF was used to analyze RNAPII CTD phospho-*Ser2* levels. (*n* > 400 for each individual bar. For box plots, center lines indicate median, box limits are the first and third quartiles, and whisker ends represent 10 to 90% confidence intervals. Data not included between the whiskers are plotted as dots.)

The crystal structure of the human P-TEFb complex (Protein Data Bank [PDB]: 3BLQ) (45) indicates that CDK9(C95) lies close to where CyclinT1 binds CDK9 (*SI Appendix*, Fig. S20*A*). Hence C95 could be the site of electrophile sensing within CDK9. We first confirmed that Halo-CDK9(C95A) was kinase active since its ectopic expression—which effectively replaces endogenous CDK9(wild type [WT]) with this mutant, as described above—did not affect RNAPII CTD phospho-*Ser2* levels (relative to empty vector- or WT-CDK9–transfected cells) (*SI Appendix*, Fig. S14*A*). Replicating T-REX–assisted hydroxynonenal delivery to CDK9(C95A) showed significantly reduced (60%) hydroxynonenylation relative to what was observed with WT-CDK9 (Fig. 3*A* and *SI Appendix*, Fig. S20*B*). Suppression of hydroxynonenylation in CDK9(C95A) was equal to suppression upon CyclinT1 overexpression (*SI Appendix*, Fig. S20*B*). Furthermore, CyclinT1 overexpression did not affect CDK9(C95A) hydroxynonenylation (*SI Appendix*, Fig. S20*B*), indicating that the regulation of residual hydroxynonenylation in CDK9(C95A) is distinct from the predominant mode of CDK9 hydroxynonenylation.

Similar results were observed under bolus hydroxynonenal treatment (*SI Appendix*, Fig. S14 *B*, *Inset*, bar 1 vs. bar 4). Intriguingly, under these conditions, parallel analysis of hydroxynonenylation of endogenous CDK9 showed that in cells expressing hydroxynonenal–sensing-competent Halo-CDK9 (either no LS or NES tagged), endogenous CDK9 was also more electrophile sensitive. Conversely, in cells expressing hydroxynonenal–sensing-incompetent constructs, namely Halo-NLS-CDK9 or Halo-CDK9(C95A), endogenous CDK9 was refractory to hydroxynonenal (*SI Appendix*, Fig. S14 *B*, *Inset*, bars 3 and 4 vs. bars 7 and 8).

We finally validated that CDK9 expressed at endogenous levels can be modified by endogenous hydroxynonenal at C95. CDK9 knockout (KO) lines were created using CRISPR-*cas9* and a guide RNA targeting CDK9 (*SI Appendix*, Table S4). These lines were rescued by transduction with a virus delivering either Flag-CDK9(WT) or Flag-CDK9(C95A) and GFP as a marker expressed bicistronically (*SI Appendix*, Fig. S20*C* and Table S1). Sorting for cells with similar, low GFP-expression levels, gave Flag-CDK9(WT)– and Flag-CDK9(C95A)–expressing polyclonal cells with close-to-endogeonous levels of CDK9 expression (*SI Appendix*, Fig. S20 *D* and *E*). When these cells were immunoprecipitated with an anti-hydroxynonenal antibody and blotted for Flag, CDK9(WT) was significantly more enriched than CDK9(C95A) (Fig. 3*B* and *SI Appendix*, Table S5). Thus, C95 functions as a site-specific electrophile sensor when CDK9 and hydroxynonenal are present at physiologically relevant levels in nonhydroxynonenal-treated cells. Mass spectrometry of immunoprecipitated endogenous CDK9 from either lysates of untreated HEK293T and HeLa cells or the same cell lines treated with excess hydroxynonenal identified adduction at lysine (K345) (*SI Appendix*, Tables S6–S10). This result, i.e., identification of modification at a low-reactivity but thermodynamically more stable site, gives further credence for a second modification site, as indicated by the anti–HNE-IP experiments above (Fig. 3*B* and *SI Appendix*, Fig. S20 *C–E*), as well as by the fact that CDK9(C95A) retained ∼40% of hydroxynonenal signal found on CDK9(WT) following T-REX (Fig. 3*A* and *SI Appendix*, Fig. S20*B*), although this residual signal did not give rise to a functional output, as our subsequent series of experiments collectively showed.

### Hydroxynonenylation Downregulates CDK9 Activity.

Accumulating data show that target- (and site-)specific hydroxynonenylation of first responders alters their activity/function (2). Given that CDK9 hydroxynonenylation is competitive with CyclinT1 association, we predicted that hydroxynonenylation would reduce P-TEFb activity, which would decrease RNAPII CTD phospho-*Ser2* levels. As our data already had indicated the functional significance of nuclear/cytoplasmic translocation and the role of HEXIM1—a protein that negatively regulates P-TEFb—in the process, we tested our predictions directly in cells using T-REX (10, 23), as we have done for several other proteins (*SI Appendix*, Fig. S1*B*). A time-course measurement following CDK9 hydroxynonenylation showed a lag phase and then a rapid drop in RNAPII CTD phospho-*Ser2* levels between 3 and 7 h by both Western blot (Fig. 3*C* and *SI Appendix*, Fig. S21*A*) and immunofluorescence (IF) (Fig. 3*D*). The magnitudes of these effects varied, although they were in the region of two- to fourfold at maximum.

**Fig. 4.**
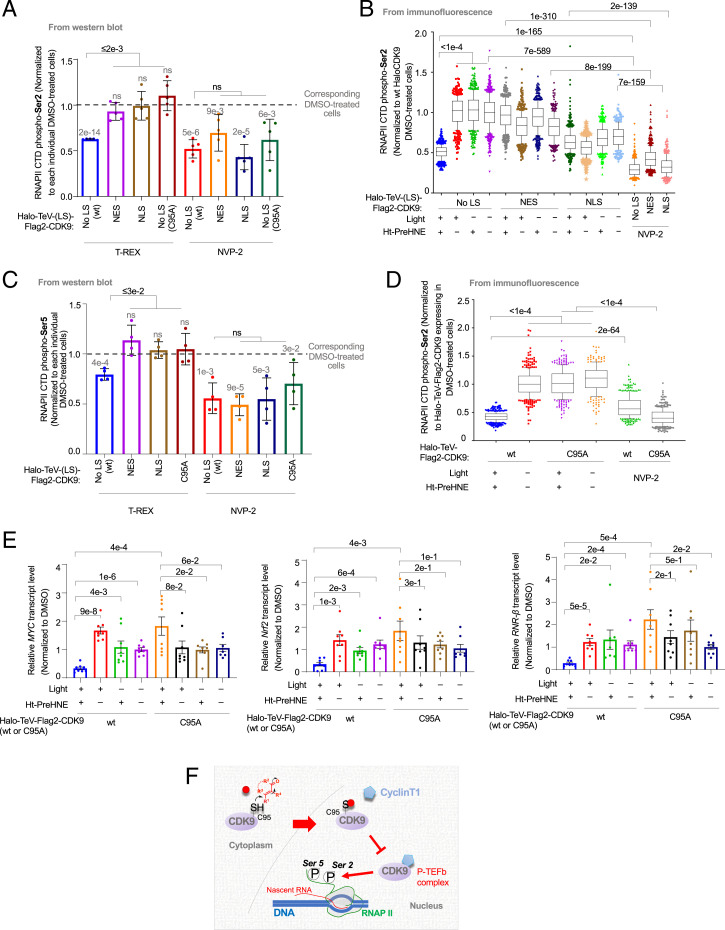
Cytoplasm-specific CDK9(C95) hydroxynonenylation selectively downregulates RNAPII CTD phospho-*Ser2* in a dominant-negative manner, with consequent reduction in the abundance of short-lived mRNAs (*SI Appendix*, Figs. S21–S25). (*A*) HEK293T cells ectopically expressing the indicated Halo-TeV-(LS)-Flag2-CDK9 constructs were subjected to T-REX and harvested for analysis 7 h post–CDK9-specific hydroxynonenylation. [See Fig. 2 *A*, *Top*, for orientation of tags within different constructs; C95A indicates Halo-TeV-Flag2-CDK9(C95A).] In parallel experiments, the same cells were treated with ATP-competitive CDK9 inhibitor (NVP-2) (200 nM, 2 h). The relative extent of RNAPII CTD phospho-*Ser2* was analyzed by Western blot using anti-RNAPII CTD phospho-*Ser2* antibody. Bars are normalized to RNAPII CTD phospho-*Ser2* levels in dimethyl sulfoxide (DMSO)-treated cells expressing the corresponding Halo-TeV-(LS)-Flag2-CDK9 construct in each case (levels of which are shown as a gray dashed line). Numbers above each bar denote *P* value from two-tailed *t* test between the bar and DMSO-treated cells expressing the corresponding Halo-TeV-(LS)-Flag2-CDK9 construct in each case (levels of which are shown as a gray dashed line) (*n* = 5 biological replicates, error bars indicate SD) (*SI Appendix*, Figs. S21 *B* and *C*, S22, and S23). (*B*) Similar experiment to *A* but IF analysis was used (note: *y* axis = 1.0 corresponds to the value from DMSO-treated cells expressing WT-HaloCDK9, i.e., fourth bar from left; and the rest of the data were normalized against this value). (*n* > 400 for each individual bar. For box plots, center lines indicate medians, box limits are the first and third quartiles, and whisker ends represent 10 to 90%. Data not included between the whiskers are plotted as dots.) (*C*) Similar experiment to *A* but RNAPII CTD phospho-*Ser5* levels were examined. Bars are normalized to RNAPII CTD phospho-*Ser*5 levels in DMSO-treated cells expressing the corresponding Halo-TeV-(LS)-Flag2-CDK9 construct in each case (levels of which are shown as a gray dashed line). Numbers above each bar denote *P* value from two-tailed *t* test between the bar and DMSO-treated cells expressing the corresponding Halo-TeV-(LS)-Flag2-CDK9 construct in each case (levels of which are shown as a gray dashed line) (*n* = 4 biological replicates, error bars indicate SD) (*SI Appendix*, Figs. S21 *B* and *C*, S22, and S23). (*D*) Similar experiment to that in *B* but comparison was made between Halo-TeV-Flag2-CDK9(WT) and -(C95A). (*n* > 400 for each individual bar. For box plots, center lines indicate medians, box limits are the first and third quartiles, and whisker ends represent 10 to 90%. Data not included between the whiskers are plotted as dots.) (*E*) HEK293T cells expressing either Halo-TeV-Flag2-CDK9 (WT) or Halo-TeV-Flag2-CDK9 (C95A) were subjected to T-REX or T-REX controls, and cells were harvested 5 h post–T-REX execution (the point at which RNAPII CTD phospho-*Ser*2 levels first reached their lowest levels). RNAs were extracted and reverse transcribed, priming off the poly-A tail. The levels of mRNAs encoding MYC, Nrf2, and RNR-β were then analyzed using qRT-PCR; insert validity was confirmed by product gel and melting-point analysis. The levels of each transcript at each timepoint were normalized against *GAPDH* (housekeeping gene) in each instance, with levels of each transcript also being normalized to its own 0-h timepoint (*n* = 8 biological replicates, error bars indicate SEM) (*SI Appendix*, Fig. S24 *A* and *B*). (*F*) The proposed model: The kinase CDK9 senses metabolite hydroxynonenal exclusively in the cytoplasm. Hydroxynonenylated CDK9, once inside the nucleus, suppresses canonical CDK9 nuclear-specific signaling activity that together with CyclinT1 in the P-TEFb complex promotes RNAPII CTD *Ser2* phosphorylation (39). P, phosphate PTM; red dot, hydroxynonenal (and likely other electrophilic metabolites of generic chemical structure shown) (*SI Appendix*, Fig. S25).

Replicating T-REX in cells expressing NES- and NLS-tagged Halo-CDK9 showed no change in RNAPII CTD phospho-*Ser2* (Fig. 4*A* and *SI Appendix*, Fig. S21 *B* and *C*). Thus, cytoplasmic-to-nuclear translocation of hydroxynonenylated CDK9 is required for RNAPII activity regulation. Indeed, in HeLa cells, treatment with hydroxynonenal led to accumulation of nuclear CDK9 (*SI Appendix*, Fig. S22 *A–C*). This occurred similarly to HeLa cells treated with leptomycin B (*SI Appendix*, Fig. S22*C*). Conversely, the ATP-competitive CDK9 inhibitor, NVP-2 (46, 47), suppressed RNAPII CTD phospho-*Ser2* in all cases studied (Fig. 4*A*). Similar outcomes were observed by IF (Fig. 4*B*). We also investigated how CDK9 hydroxynonenylation enabled by T-REX affected RNAPII CTD phospho-*Ser5*, an event required for promoter clearance and RNAPII pausing for 5′ capping. There was a relatively small extent of RNAPII CTD phospho-*Ser5* suppression following CDK9 hydroxynonenylation (Fig. 4*C* and *SI Appendix*, Fig. S21 *B* and *C*). NVP-2 once again induced significant suppression of RNAPII CTD phospho-*Ser5*.

Neither RNAPII CTD phospho-*Ser2* nor -*Ser5* levels were affected when T-REX was replicated in cells expressing the sensing-defective but otherwise-functional mutant, Halo-CDK9(C95A). This result confirms that the responses were specific to CDK9(C95) site-specific hydroxynonenylation. However, cells expressing Halo-CDK9(C95A) were sensitive to NVP-2 (Fig. 4 *A* and *D*), again confirming that phosphor-*Ser2* regulation remains functional and that Halo-CDK9(C95A) is kinase active. Similar results were found when RNAPII *pan*-phosphorylation was measured (*SI Appendix*, Fig. S23).

### CDK9 Hydroxynonenylation at C95 Alters the Abundance of Short-Lived mRNAs.

These data indicate that CDK9-specific hydroxynonenylation may suppress transcription. Such a process would by default deplete short-lived messenger ribonucleic acids (mRNAs), such as Cyclin-D, Cyclin-E, Nrf2, MYC, and ribonucleotide reductase-β. We first confirmed that the half-life of these mRNAs remained, for the most part, the same between Halo-CDK9–expressing cells and empty-vector–transfected cells (*SI Appendix*, Fig. S24*A* and Table S11). This result further supports the observations above (*SI Appendix*, Figs. S14*A* and S17*A*) that ectopic expression of Halo-TeV-Flag2-CDK9 does not affect P-TEFb activity. We then measured the changes in the abundance of these mRNAs at a time point closely following the onset of RNAPII CTD phospho-*Ser2* suppression, i.e., 5 h post–T-REX-enabled CDK9 hydroxynonenlyation, relative to T-REX technical controls (Fig. 4*E* and *SI Appendix*, Fig. S24*B*). A significant decrease in mRNA levels was measured under these conditions. Replicating these experiments in cells expressing Halo-TeV-Flag2-CDK9(C95A) did not suppress mRNA levels, relative to all controls (Fig. 4*E* and *SI Appendix*, Fig. S24*B*). Thus, fractional electrophile labeling of CDK9(C95) in the cytoplasm, even when CDK9 is present at endogenous levels, significantly reduces mRNA levels.

## Discussion

This paper underscores the nuanced and surprisingly diverse regulation that can be enacted by arguably “unsophisticated” electrophilic metabolites on numerous (at least 32) proteins. Although this level of regulation is most apparent when studying spatiotemporally-controlled electrophile engagement processes (i.e., T-REX and G-REX) (48), it is retained in a very commonly-deployed approach to study effects of electrophiles on cells, namely, hydroxynonenal-bolus dosing (2, 4, 9, 33). These consistent outputs give good confidence that such sensing is a physiologically-relevant process retained both under stress (high electrophile dose) and under spatiotemporally-regulated signaling processes mimicked by Localis-rex. Such a posit was confirmed through detection of endogenous CDK9 hydroxynonenylated at C95. One critical conclusion from this study is that electrophile sensing is partitioned across different subcellular locales for numerous proteins and that maximum electrophile occupancy on many electrophile-sensor proteins is likely to be less than 100%. It has long been assumed that ligand occupancy is the main determinant of physiological activity of biological electrophiles (2, 13). This assumption underpins biochemistry and is the cornerstone of most go-to methods to understand signaling roles of biological and medicinal electrophiles and to rank their sensitivities. The divergent sensing properties we show here are unprecedented and previously overlooked in target-profiling/ranking systems, likely because tools to decode subcellular-specific privileged sensing functions have remained limited and such ability in intact cells is currently unknown.

Of course, the limitation of stoichiometry could hamper the biological relevance of metabolite-sensing events. However, as our data show here, such low-occupancy modifications, even in regions where the protein is seemingly inactive and/or present in low abundance, can usher in important downstream signaling events. For instance, treatment with a saturating amount of CDK9 inhibitor gave ∼65% suppression (at maximum) of both RNAPII CTD phospho-*Ser2* and -*Ser5*. Conversely, less than 20% hydroxynonenylation of Halo-CDK9 affected RNAPII CTD phospho-*Ser2* levels by 40%, with more modest effects on RNAPII CTD phospho-*Ser5*. These results are puzzling, but clearly stoichiometry-restricted modifications must be able to manifest phenotypic effects even at low stoichiometry, if they are to be biologically relevant. There is indeed a growing number of such examples where electrophile modification rewires signaling pathways at low target occupancy in the literature. We have also been able to develop isoform-specific inhibitors around such an emerging paradigm (10–12). A question of growing importance is thus how such dominant processes come about. That will be addressed in further work, although a number of mechanisms could be used by different proteins, so paradigmatic examples are at present limited.

Furthermore, in the case of CDK9, even though sensing occurred in the cytoplasm, retro-translocation to the nucleus was required for the modified protein to impact RNAPII CTD phospho-*Ser2* and transcription (Fig. 4*F* and *SI Appendix*, Fig. S25). Thus, it appears that electrophiles, which likely have relatively low diffusion distances, coopt proteins to transfer information from the cytosol to the nucleus. This concept of the “target becoming the signal” could well become paradigmatic and indeed fits well with the dominant-negative signaling processes that we and others have reported. The cytosol-to-nuclear communication is further reminiscent of canonical signal-transduction mechanisms, wherein extracellular signals interact with surface proteins, triggering cascades ending in the nucleus. However, as we have hypothesized previously (5, 12), in the case of electrophile sensors, both the signal recognition and the downstream signaling event are choreographed by the same protein.

Such sensitive trigger switches that are essential genes are particularly interesting and their ramifications deserve further investigation. For instance, such regulatory mechanisms could be beneficial for drug design, because nontransformed cells should be much less susceptible to this mode of regulation than transformed cells. We anticipate that similar comparisons of organelle-specific electrophilic-metabolite sensing will uncover other elaborate and covalent drug-relevant regulatory mechanisms.

## Materials and Methods

All chemicals used in this work unless otherwise indicated were from Sigma and purchased at highest available purity. NVP-2 was purchased from MCE (MedChemExpress) HaloTag-targetable photocaged precursor to hydroxynonenal [HNE(alkyne)] (namely, Ht-PreHNE) was synthesized as described previously (25). The 4-HNE (32100) was purchased from Cayman Chemical. TransIT 2020 and TransIT LT1 transfection reagents were from Mirus Bio. Leptomycin B (L2913) and Actinomycin D (A9415) were from Sigma-Aldrich. Cyanine5 (Cy5)-azide and Copper(II)-TBTA complex were from Lumiprobe. Dithiothreitol (DTT), streptomycin sulfate, and tris(2-carboxyethyl)phosphine (TCEP)-HCl were from Goldbio Biotechnology. Calf intestinal alkaline phosphatase (CIP) was from NEB (M0290S). Biotin-dPEG_11_-azide (10784) was from Quanta BioDesign. Pierce high-capacity streptavidin agarose beads were from Thermo Fisher (20361). ANTI-Flag M2 affinity gel (A2220) was from Sigma-Aldrich. Pierce Protein G agarose beads were from Thermo Fisher (20397). The 3× Flag peptide was from APEXBIO (A6001). Anti-HA monoclonal agarose (A2095) was from Sigma-Aldrich. Bovine serum albumin (BSA) powder was from Thermo Scientific. Phusion HotStart II polymerase was from Thermo Scientific. All restriction enzymes were from NEB. Complete EDTA free protease inhibitor was from Roche (Sigma Aldrich). The 1× RIPA buffer was from Santa Cruz Biotech. The 1× Bradford dye was from BioRad. The plasmids for recombinant expression of TeV protease (pRK793; Addgene 8827), Flag-CDK9 (Addgene 28100) were from Addgene. BL21 (DE3)-RIL codon plus cells were from Stratagene. His_6_-TEV S219V protease was recombinantly expressed and purified from BL21(DE3)-RIL cells using Ni-NTA resin (Thermo Fisher) as previously described (25). HEK293T, HeLa, RAW 264.7, and U2OS cells were from American Type Culture Collection (ATCC). The 1× Dulbecco‘s phosphate-buffered saline (DPBS) (Gibco 14190250), 1× Trypsin (TrypLe; Gibco 12604021), 100× NEAA (Gibco 11140050), 100× sodium pyruvate (Gibco 11360070), 100× penicillin-streptomycin (Gibco 15140122), DMEM (Gibco 41966029), and MEM+Glutamax media (Gibco 41090093) were from Life Technologies. Fetal bovine serum (FBS) was from Sigma Aldrich (F2442). Photouncaging was performed using Spectroline and Camag ultraviolet (UV) lamps (for illumination of small area, ENF-240C [Spectroline] and UV lamp 4 [Camag]; if larger surface areas were needed, XX-15N [Spectroline]). The lamps were positioned above a confluent monolayer of cells such that the power of UV irradiation was ∼5 mW/cm^2^ on samples (measured by a light intensity sensor [Spectroline, XDS-1000]). For all confocal imaging experiments, a Zeiss LSM710 confocal microscope was used. Quantitation of fluorescence intensity was performed using Image-J software (NIH, version 1.52e). In-gel fluorescence analysis, imaging of Western blots, and Coomassie staining of gels were performed using a BioRad Chemi-Doc MP Imaging system and a Vilber Fusion FX imaging system. RT-qPCR analysis was performed using Thermo Fisher QuantStudio 6. A Cy5-excitation source by epi-illumination and a 695/55 emission filter were used. Cell counting was done by Thermo Fisher Countess II FL (A25750). All microplate-based assays, such as Bradford protein assay, were performed using a BioTek Cytation 3 Cell Imaging Multimode reader with dual reagent injectors. All sterile cell culture plasticware were from CellTreat, except for glass-bottomed dishes used for imaging that were from In Vitro Scientific (D35-20-1.5-N). Mitotracker Red CMXRos (mitochondria) was from Thermo Fisher (M7512). *Mycoplasma* testing for all the cell lines in this work was performed using the Sigma LookOut PCR detection kit (MP0035) every 3 mo. TRIzol reagent (15596018) was from Ambion. Superscript III (18080085) and RNaseOUT (10777019) enzymes for qPCR experiment were from Thermo Fisher. SYBR Green Master Mix reagent for RT-qPCR was from Bio-Rad. Proteomics data are provided in a single Excel file (corresponding to Dataset S1). Asp-N (V1621) and Arg-C (V1881) for mass spectrometry experiments were from Promega. Information on cloning primers, shRNA sequences, small interfering RNA (siRNA) sequences, guide RNA sequence, antibodies, and RT-qPCR primers used are listed in *SI Appendix*, Tables S1–S5 and S11, respectively.

### Safety Comments.

All experiments were performed according to standards of operational practice in compliance with the expected standard operational procedures for chemical safety and biosafety implemented by institutional safety compliance guidelines.

### Validation of Antibodies.

Many of the antibodies were themselves used to show knockdown of endogenous proteins using multiple sh/siRNAs, wherever possible. The antibody specificities of the transgenes of interest were verified using an empty vector as control. In detail, for many experiments, results were replicated by detecting ectopic expression of an epitope-tagged version (where the gene of interest from both nontransfected and transfected cells can be viewed in the same frame, further validating the specificity of the antibody). By this metric, we confirmed that the data for ectopically overexpressed proteins are consistent with the IF data for the endogenous protein. Wherever possible, anti-Flag/-HA/-HaloTag was used to detect the ectopic proteins, thereby eliminating doubts about antibody specificity. Halo protein was confirmed to be expressed ubiquitously throughout the transfected cells and it was confirmed that the photocaged small-molecule probe (Ht-PreHNE) colocalized with Halo, validated by Click assay using Cy5-azide.

### Cell Growth and Culture Maintenance.

HEK293T and HeLa cells were maintained in 1× MEM + Glutamax media supplemented with 10% FBS, 1× NEAA, 1× sodium pyruvate, and 1× Pen-Strep. U2OS and RAW 264.7 cells were cultured in 1× DMEM media supplemented with 10% FBS and 1× Pen-Stre. (Gibco catalog number as in Materials and Methods).

For Localis-rex protocol, SILAC HEK293T cells were cultured and passaged at least five times (more than 2 wk) before using. The culturing media consisted of, in final concentrations, SILAC drop-off media 1× DMEM (Thermo Fisher 89985), 10% dialyzed FBS (Sigma-Aldrich F0392), 1× sodium pyruvate (Gibco 11360070), 1× Pen-Strep (Gibco 15140122), and the corresponding light/heavy amino acids. For light amino acids, in final concentrations, 146 μg/mL of L-lysine (Sigma-Aldrich L8662) and 84 μg/mL of L-arginine (Sigma-Aldrich A8094) were used and the same concentrations for heavy amino acids, L-lysine-^13^C_6_, ^15^N_2_ hydrochloride (Sigma-Aldrich 608041) and L-arginine-^13^C_6_, ^15^N_4_ hydrochloride (Sigma-Aldrich 608033), respectively. Other culturing/handling methods are the same as for normal HEK293T cells.

### Localis-rex in Living Cells.

HEK293T cells were cultured in SILAC media described previously. After 2 wk of culturing, cells were transfected with locale-specific Halo plasmid (NLS or MOMLS) using TransIT-2020 transfection reagent per the manufacturer’s recommendation. Subsequent steps were performed under red light. Thirty-six hours posttransfection, cells were treated with 20 μM Ht-PreHNE in serum-free media and incubated for 2 h, followed by gentle rinsing with serum-free media three times for every 30 min over the next 1.5 h. UV lamps were turned on 10 min prior to use (light source: 365 nm, ∼5 mW/cm^2^ hand-held UV-lamp placed 10 cm above samples). For samples designated as “samples exposed to light,” lids were removed from the culture dishes and cells were irradiated for 5 to 8 min. The cells were harvested, washed two times with ice-cold DPBS, and frozen in liquid nitrogen. Cell pellets were lysed in 300 µL buffer containing 50 mM Hepes (pH 7.6), 150 mM NaCl, 1% Nonidet P-40, 1× Roche cOmplete, mini, EDTA-free protease inhibitor mixture, and 0.3 mM TCEP by rapid freeze–thaw (×3). Cell debris was removed by centrifugation at 18,000 × *g* for 8 to 10 min at 4 °C. Protein concentration of the clarified lysate was determined using Bradford assay. After cell lysis, Click coupling with biotin-azide (as described in biotin/streptavidin pulldown procedure elsewhere in *SI Appendix*, *Supplementary Methods*), and pulldown enrichment by Streptavidin agarose beads, the enriched protein was eluted using 3× Laemmli dye containing 6% βME at 98 °C for 10 min. The sample was subjected to SDS-PAGE followed by Coomassie stain and the gel was excised into five pieces and sent for MS identification (see detailed methods regarding in-gel trypsin digestion of SDS gel bands, protein identification by nano-LC/MS/MS analysis, and LC-MS/MS data analysis reported elsewhere in *Materials and Methods*).

## Supplementary Material

Supplementary File

Supplementary File

## Data Availability

The mass spectrometry proteomics data have been deposited to the ProteomeXchange Consortium via the Proteomics Identifications Database (PRIDE) (50) partner repository with the dataset identifier PXD017774.
