# Supplementary material for: Function-guided proximity mapping unveils electrophilic-metabolite sensing by proteins not present in their canonical locales
Source: Proc Natl Acad Sci U S A. 2022 Jan 26;119(5):e2120687119. doi: 10.1073/pnas.2120687119 (PMC8812531; doi:10.1073/pnas.2120687119)
Supplement: Supplementary File [file pnas.2120687119.sapp.pdf]

PNAS
www.pnas.org

Supplementary Information

Function-guided proximity mapping unveils electrophilic-metabolite sensing by proteins not present in their canonical locales

Yi Zhao[1-3], Pierre A. Miranda Herrera[1,2], Dalu Chang[1,2], Romain Hamelin[4], Marcus J. C. Long[2,5]*, and Yimon Aye[1,2]*

[1]Swiss Federal Institute of Technology Lausanne (EPFL), 1015 Lausanne, Switzerland

[2]NCCR Chemical Biology and University of Geneva, 1211 Geneva, Switzerland

[3]BayRay Innovation Center, Shenzhen Bay Laboratory, Shenzhen 518055, Guangdong, China

[4]Proteomics Core Facility, School of Life Sciences, Swiss Federal Institute of Technology Lausanne (EPFL), 1015 Lausanne, Switzerland

[5]University of Lausanne (UNIL), Lausanne, Switzerland

*Co-correspondence

Primary contact: yimon.aye@epfl.ch

Supplementary figures (pp 2-38),

Full-view data from western blot and FACS analyses (pp 39-56),

Method details, including antibody validations (pp 57-66),

Statistical analysis (pp 67-77),

Supplementary datasets (pp 78-79),

Supplementary tables (pp 80-96),

Supplemental references (p 97)

Supplementary figures:

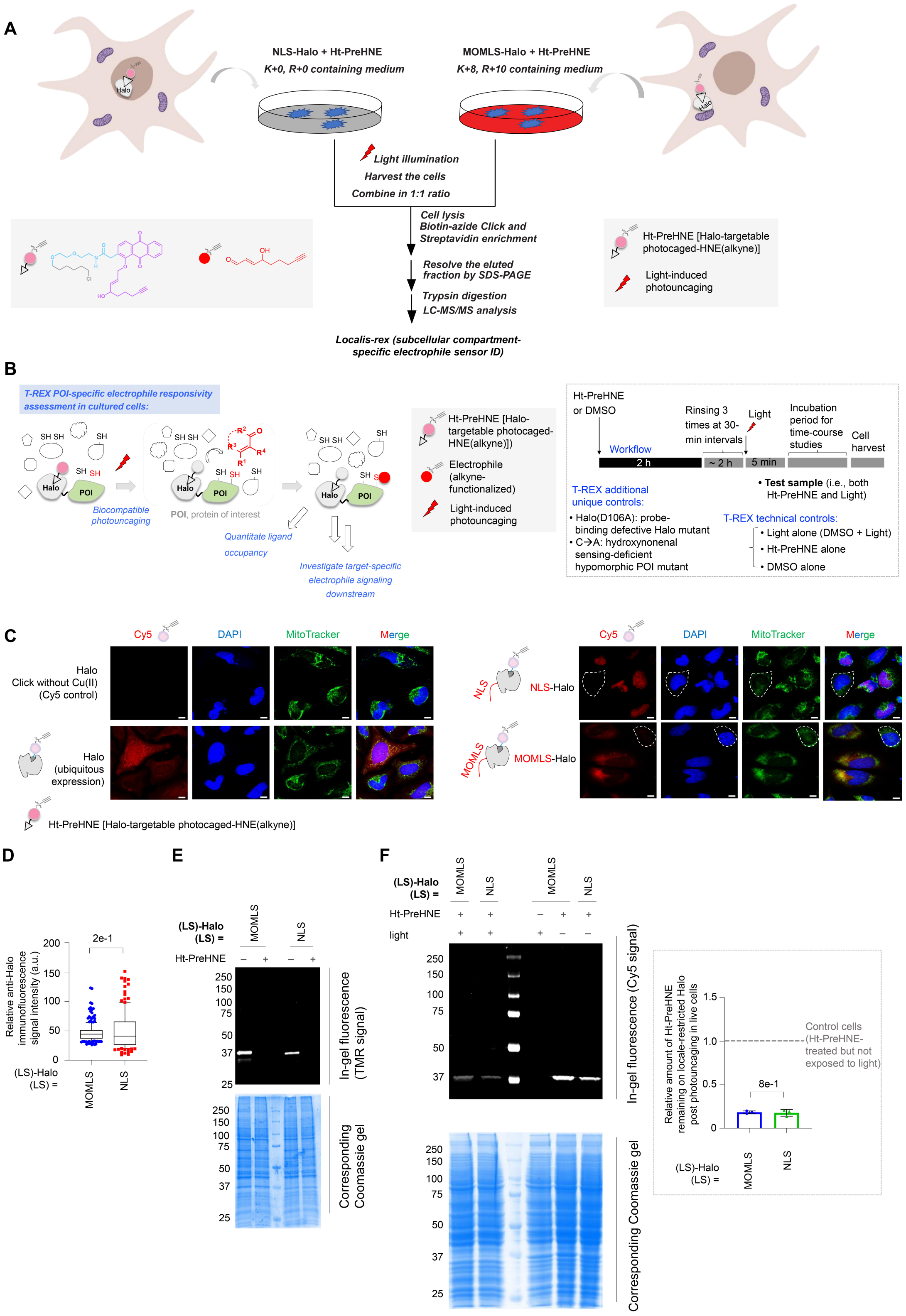

Supplementary Figure 1. **Localis-rex enables quantitative locale-specific sensor ID**.

(**A**) Workflow for Localis-rex, exemplified for the first of the three replicates. Also see **Fig.1A** and **S2A**. To quantitatively ID locale-specific sensors for the native electrophilic metabolite hydroxynonenal and related Michael-acceptor electrophiles, SILAC-labeled HEK293T cells expressing Halo, genetically encoded with a specific localization sequence (LS), were treated with photocaged-hydroxynonenal(alkyne-functionalized) [Ht-PreHNE[1,2] (20 µM, 2 h)], followed by 3 rinses to remove unbound Ht-PreHNE. Following light exposure (5 min, 5 mW/cm^2 at 365 nm), cells were further incubated (5 min) prior to lysis followed by Click-biotin-pulldown-assisted proteomics-based target ID.

(**B**) Workflow of T-REX method[1-4] enables top enriched sensors identified by Localis-rex to be validated for individual-protein-specific sensing capability and signaling consequences. Briefly, a specific protein of interest (POI) (ID'ed by Localis-rex) is expressed as a Halo-fusion (Halo-POI). Following treatment with the same photocaged-electrophile used in Localis-rex (here, Ht-PreHNE) and rinsing, light exposure (same conditions as above) gives the POI first refusal to the liberated electrophilic-signal before it diffuses from the POI, offering a genuine test of POI's electrophile responsivity in living systems. Measurements of any downstream events post T-REX permit *POI-electrophilic-modification-specific time-dependent* cell responses to be assayed directly (5-7 h in this study). ***Inset*** (dotted rectangular box) shows workflow and both technical controls (DMSO alone, light alone, or photocaged-probe alone) and unique controls (D106A-Halo-mutant, or sensing-deficient-POI-mutant) used in this study.

(**C**) Imaging analysis to validate expected localization of Ht-PreHNE covalently bound to Halo in Localis-rex, thereby confirming the electrophile release within the designated subcellular locale. Here, Halo is expressed either ubiquitously (i.e., no LS), or in nucleus (NLS) or mitochondrial-outer-membrane (MOMLS). Representative IF images of HeLa cells ectopically expressing the indicated Halo-construct that were treated with Ht-PreHNE, rinsed, and fixed. Localization of Ht-PreHNE-bound Halo was subsequently analyzed by Click coupling to Cy5-azide and counterstained by DAPI (nucleus) and Mitotracker Red CMXRos (mitochondria). NLS, nuclear-localization sequence-tagged. MOMLS, mitochondria-outer-membrane-localization sequence-tagged. Ht-PreHNE, Halo-targetable photocaged-HNE(alkyne)[2,5]. See also **Fig.1A & S1A.** The dotted white lines on right hand side indicate cells of low Halo-transfection efficiency (***note***: all cells in the frame showing Halo-"ubiquitous expression" samples express detectable Halo protein). These dotted cells serve as an internal control for any background labeling associated with Cy5-azide Click-coupling. Scale bars, 2 µm.

(**D**) Quantification of Halo expression levels: representative images in (**C**). (n > 130 for both bars. For box plots, center lines indicate medians, box limits are the first and third quartiles and whisker ends represent 10–90% confidence intervals. Data not included between the whiskers are plotted as dots).

(**E**) Blocking experiments using Ht-PreHNE and TMR-Cl showed that Ht-PreHNE fully saturates Halo-binding site for each (LS)-Halo (LS = MOMLS, or NLS) construct. HEK293T cells ectopically expressing (LS)-Halo (LS = MOMLS, or NLS) were treated by either Ht-PreHNE (20 µM) or DMSO for 2 h followed by rinsing cycles to remove excess unbound Ht-PreHNE, prior to harvesting. Normalized cell lysates were treated with 3 µM TMR-Cl (Halo-targetable TMR dye) for 30 min at 37 °C, and analyzed by in-gel fluorescence (detecting TMR-signal, and reporting active HaloTag protein, *upper panel*) and corresponding Coomassie gel (reporting protein loading, *lower panel*). The lower band (around ~33 kDa) with weaker signal in the western blot is HaloTag protein alone, likely resulting from cleavage during lysis.

(**F**) Photouncaging efficiency remained similar for Ht-PreHNE when it was bound to (LS)-Halo expressed in different locales (using either MOMLS- or NLS-tags). See also **Fig.S1C**. HEK293T cells ectopically expressing (LS)-Halo (LS = MOMLS, or NLS) were treated by either Ht-PreHNE (20 µM) or DMSO for 2 h followed by rinsing cycles to remove excess unbound Ht-PreHNE. Following light exposure (5 min, 5 mW/cm^2 at 365 nm), cells were further incubated (5 min) prior to lysis, followed by Click coupling to Cy5-azide to visualize residual Ht-PreHNE that had not undergone photouncaging and thus remained covalently bound to (LS)-Halo. ***Inset***: quantification of the relative amount of Cy5 signal (reporting Ht-PreHNE) that remained on Halo post light exposure. (n = 3 biological replicates, error bars indicate s.d.; two-tailed *t*-test was applied). Horizontal dotted line designates the relative value of corresponding Cy5 signal from the cells treated with Ht-PreHNE only but no light, which was set to a value of 1.0.

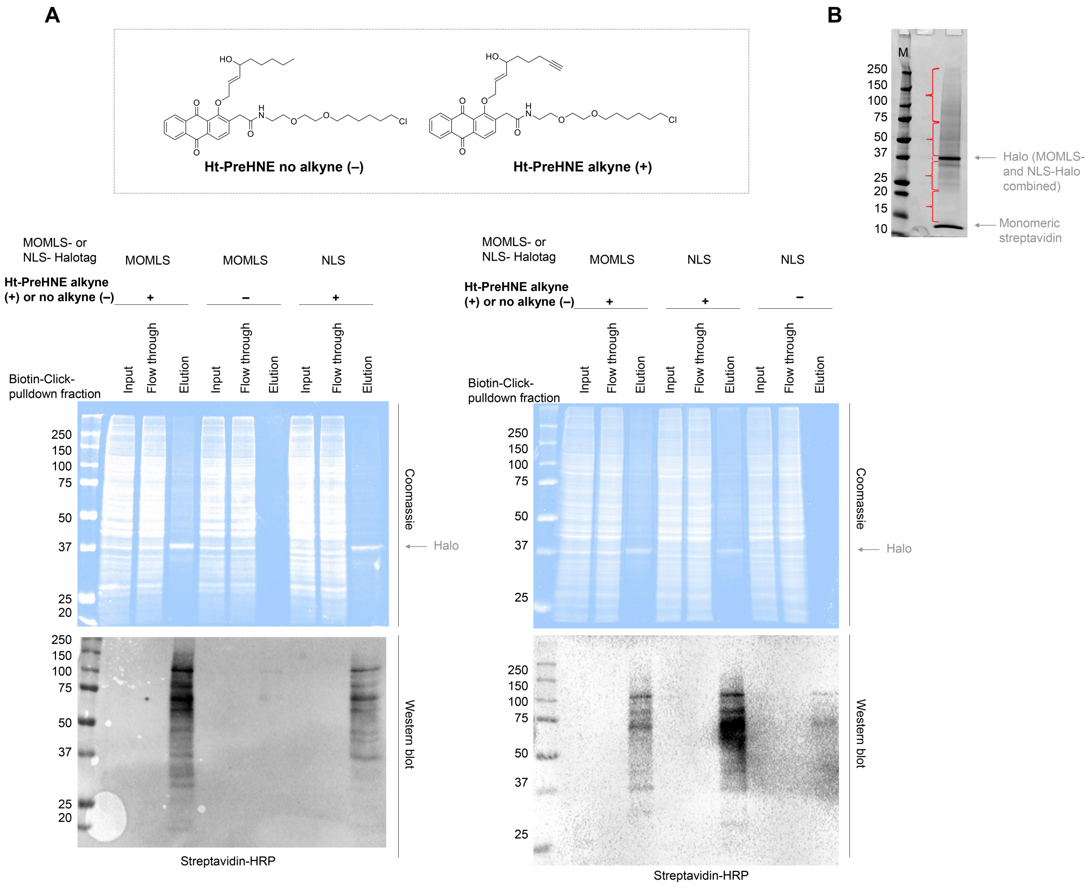

Supplementary Figure 2. Following locale-specific delivery of hydroxynonenal at precise time and dosage in live cells, locale-specific first responders to hydroxynonenal were captured using standard biotin-pulldown procedure and these proteins were identified by first resolving the eluted samples on SDS-PAGE and subsequent digestion and proteomics analysis (See supplemental methods for details).

(A) Validation of enrichment procedure. Halo-targetable photocaged hydroxynonenal that had not been alkyne-functionalized (indicated as '**—**' in the figure, also see ***inset*** at top) was deployed to account for non-specific binding. Locale specificity was achieved by genetically fusing Halo to nuclear localization sequence (NLS) or mitochondrial outer-membrane-targeted localization sequence (MOMLS) (also see **Fig.S1C**). Following procedures in live cells (see supplemental methods), cells were lysed and lysates were subjected to Click coupling to biotin-azide according to the procedure described in SI. **Note:** 'Ht-PreHNE alkyne' is equivalent to 'Ht-PreHNE' used throughout the manuscript and in all figures. When there is no alkyne-functionalization, the corresponding probe is consistently called 'Ht-PreHNE no alkyne', as in this figure.

(B) Following Click coupling to biotin-azide according to the procedure described in SI, eluted samples were resolved by denaturing gel-electrophoresis (4-20 % gradient gel, Biorad #4561095, 150 volts for 55 min, and stained with Colloidal Coomassie to visualize the proteins). See supplemental methods for the detailed procedure. Representative sections of the gel excised were shown. Note: equal amounts of MOMLS-Halo and NLS-Halo-expressing cells were mixed together, post the execution of probe treatment and photouncaging procedures performed in both samples. The resulting pooled cells were subjected to lysis and Click coupling to biotin-azide according to the procedure described in Supplemental Methods. Thus, the Halo band (~37 kDa) reflects ~1:1 combination of MOMLS-Halo and NLS-Halo. Also see **Fig.S1D-F**.

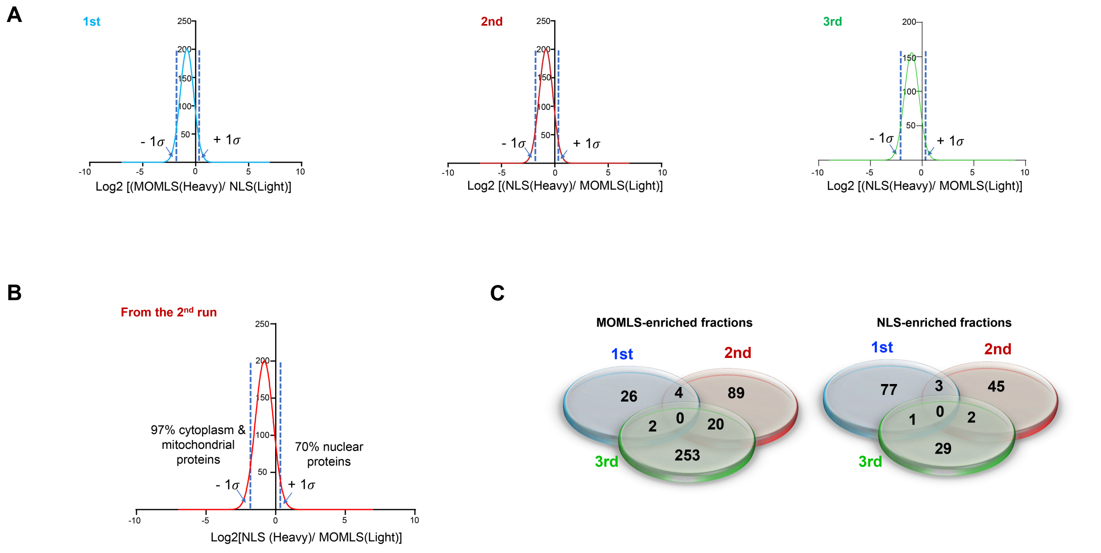

D

Group	Category	Potentially locale-specific hydroxynonenal sensors	Known hydroxynonenal sensors	% of hits validated for sensing	% of hits validated for locale-specific sensing
1	Hits 2X; Null 1X (9 hits)	ATIC, ACO2, CDK9, DAGLB, GGCT, LCMT1, IPO7, NAPA, FAM49B	ATIC, ACO2, GGCT, LCMT1, IPO7, FAM49B	89%	44%
2	Hits 2X; Middle 1X (12 hits)	ATP6V1A, CALR[a], CKMT1A, DDX17, EPHX1, FKBP8, KCTD12, PCCA, RCC1, SCCPDH, SLC25A13, TUFM	All 12 hits except EPHX1	100%	17%
3	Hits 2X; Opposite 1X (11 hits)	ANXA2, ATP5B, DSG1, FLG2, HRNR, PGRMC1, LTA4H, SERPINB3, SERPINB12, S100A8, S100A9	All 11 hits except S100A8, S100A9	82%	

- Hits in Blue are carried forward to general hydroxynonenal sensing analysis (also see Fig.S4);
- Hits in Red are validated for locale-specific hydroxynonenal sensing ability;
- Hits in Green are known hydroxynonenal-sensors previously reported, albeit without defined locale for sensing. (For clarity, these proteins are listed in the panel on the right);
- Hits in Brown are not available as Halo-fusion proteins in Halo ORF library.

[a]CALR bears an ER-localization sequence (amino acids 1-17) that is unlikely to localize correctly in Halo-POI validation assays.

Note: each row in the above panel corresponds to the respective line entries in the left panel

Supplementary Figure 3. **Selection criteria for enriched targets indexed by Localis-rex**.

(**A**) Histogram showing the Gaussian fit of protein ratios distribution from three individual SILAC replicates (1st blue, 2nd red, and 3rd green), where the x-axis indicates $\log_2$[(heavy:light)] of each protein detected in each SILAC replicate data set, and the y-axis indicates the number of proteins. For each SILAC replicate, proteins deviating from the mean by > +1σ or < -1σ (either ranked top 16% or bottom 16%) assuming a Gaussian distribution, were analyzed further as described in **Fig.S3B-D**. ***Note***: keratin/keratin-related proteins and proteins that have no designated heavy:light ratios are omitted from this analysis as previously described[6]. The list in **Dataset S1-1**, **S1-2**, **and S1-3** correspond to the data from these three replicates post removal of such proteins. Raw data for all three replicates have been uploaded to the PRIDE database. ***Note:*** the raw data in these plots are not normalized have a mean of 1.

(**B**) UniProt and the Human Protein Altas database were used to identify the predominant activity/function-based subcellular-locale for the proteins from the 2nd SILAC replicate, specifically for potentially locale-specific sensors [> +1σ or < -1σ from the assumed Gaussian distribution (either ranked top 16% or bottom 16%)].

(C) Venn diagram analysis was used to overlap hydroxynonenal-specific sensors in the specific locale (from MOMLS- or NLS-Halo) over the 3 independent (1st blue, 2nd red, and 3rd green) SILAC proteomics replicates. 32 proteins were observed from the overlap to be potentially locale-specific sensors, defined as appearing as enriched in the same locale in at least **two** of the **three** replicates. Among these 32 proteins, there were 26 proteins from MOMLS-Halo enriched fraction (left) and 6 proteins from NLS-Halo enriched fraction (right). See also **Fig.S3D,S4**.

(**D**) Potential locale-specific sensor proteins ID'ed in replicate SILAC data sets**.** All 32 proteins from both MOMLS- or NLS-enriched fractions among three SILAC data sets were pooled and analyzed based on the criteria discussed in the text (also see **Fig.S4**). *Left Panel:* Group-1: "**Hits 2X; Null 1X**" denotes proteins enriched when hydroxynonenal was released within the same locale in two replicates [with heavy:light ratio either > $+1\sigma$ or < -1σ, (either ranked top 16% or bottom 16%)], but these proteins were missing altogether in the remaining replicate. Group-2: "**Hits 2X; Middle 1X**" denotes proteins enriched when hydroxynonenal was released within the same locale in two replicates (with heavy:light ratio either > $+1\sigma$ or < -1σ), but these proteins appeared in the middle (or unenriched) group in the remaining replicate. Group-3: "**Hits 2X; Opposite 1X**" denotes proteins enriched when hydroxynonenal was released within the same locale in two replicates (with heavy:light ratio either > $+1\sigma$ or < -1σ), but these proteins were enriched in the opposite locale in the remaining replicate. See table footnote for reference to color codes. *Right panel*: Shows the number (and percentage) of sensors from the hits that were known to sense electrophiles regardless of locale; validation; and locale-specific sensing validation. Each row within this table corresponds to the respective line entries within the table on the left (left panel). See main text for discussion and also **Fig.S4**, and **Dataset S1**.

Entry	Gene name (Data are on Halo-tagged variants)	Confidence interval 1st, 2nd, and 3rd data sets, respectively	Predominant localization previously reported	Fraction enriched	Relative Halo-POI expression level (by TMR-Halo labeling) (normalized to LCMT1 as 1.0)	Halo-POI fusion protein locale (average ratio of nucleus: cytoplasm)	Sensing propensity (by in-gel fluorescence)	% Hydroxynoneny - lation efficiency (by in-gel fluorescence)	Relative locale-specific sensing (by biotin pulldown) (ratio for no-LS-tag: NES-tag: NLS-tag constructs)
1	**ATIC**	Nucleus top 10%, Nucleus top 16%, N/A	Cytoplasm	Nucleus	0.66	0.41	Yes	10	ND[a]
2	**ATP6V1A**	Cytoplasm top 5%, Middle 68%, Cytoplasm top 16%	Cytoplasm	MOMLS	0.15	0.79	ND[c]	--	ND[b]
3	**CDK9**	Cytoplasm top 5%, Cytoplasm top 5%, N/A	Nucleus*	MOMLS	0.10	0.71	ND[d]	23[e]	1:1.24:0.27
4	**DAGLB**	Cytoplasm top 8%, Cytoplasm top 10%, N/A	Plasma membrane	MOMLS	0.23	0.69	ND[d]	--	1:0.74:0.15
5	**EPHX1**	Middle 68%, Nucleus top 14%, Nucleus top 15%	ER	Nucleus	0.30	0.57	Yes	5	1:0.42:1.33
6	**FKBP8**	Nucleus top 15%, Nucleus top 10%, Middle 68%	Mitochondrial	Nucleus	0.48	0.44	Yes	16	ND[b]
7	**GGCT**	N/A Cytoplasm top 10% Cytoplasm top 8%	Cytoplasm	MOMLS	0.89	0.70	Yes	8	1:2.15:0.75
8	**LCMT1**	Cytoplasm top 12%, N/A Cytoplasm top 16%	Cytoplasm	MOMLS	1.00	0.49	Yes	19	1:0.84:0.16
9	**LTA4H**	Nucleus top 13%, Nucleus top 5%, Cytoplasm top 15%	Cytoplasm, Nucleoplasm	Nucleus	0.85	0.52	Yes	43	ND[b]
10	**NAPA**	N/A Cytoplasm top 10% Cytoplasm top 8%	Cytoplasm	MOMLS	0.25[f]	----------------------------------	ND[g]	----------------------------------	
11	**RCC1**	Cytoplasm top 8%, Cytoplasm top 5%, Middle 68%	Nucleus, Cytoplasm	MOMLS	0.79	0.99	Yes	30	1:0.48:0.07

*Catalytically-functional locale is nucleus although presence in the cytoplasm is also widely reported

Supplementary Figure 4. Winnowing the 32 top-enriched hits to 11 top-picked potential locale-specific and hydroxynonenal-signal-specific and sensors.

Representative analysis of the 11 proteins (picked from the 32 potential hits; see also **Fig.S3C-D**). ND, not determined. [a]Known hydroxynonenal sensor and thus locale-specific sensing ability was not determined. [b]In lower priority Groups, Group-2 and -3 (**Fig.S3D**) and thus locale-specific sensing ability was not determined. [c]Halo-POI construct of ATP6V1A (V-ATPase composed of cytoplasmic V1 domain and a transmembrane V0 domain) shows relatively high levels of nuclear distribution (**Fig.S6B**) and hence this fusion construct was not assayed. [d]Low protein-expression levels render the measurements below the detection limit of in-gel fluorescence assay. [e]Hydroxynonenal ligand occupancy determined by Click-biotin-pulldown assay (see **Fig.1C**). Targeting efficiency (φ) was derived from this equation:

$$\textit{ligand occupancy} = (\varphi) \times \textit{photouncaging efficiency of Ht-PreHNE,}^{1}$$

[f]Quantification includes both bands (see **Fig.S5A**: gels/blots in bottom row), corresponding to full-length Halo-NAPA and the band resulting from cleavage below 75 kDa marker (See main text discussion and **Fig.S3D**); [g]Instability to cleavage precluded further assessment of Halo-NAPA.

[1] whereby ligand occupancy was calculated from biotin-Click pulldown analysis following T-REX delivery in cells: i.e., the ratio of the anti-Flag-signal of the elution band from T-REX-treated samples (post Tev-mediated separation of Halo and Flag-POI) to the anti-Flag-signal of the input band that corresponds to the full length fusion protein in samples not exposed to light; and targeting efficiency (φ) was calculated from in-gel fluorescence analysis following T-REX delivery in cells: i.e., the ratio of the Cy5-signal on POI from T-REX-treated samples (post Tev-mediated separation of Halo and POI) to the Cy5-signal on Halo-POI fusion protein from samples not exposed to light, from which the Cy5-signal on Halo from T-REX-treated samples (post Tev-mediated separation of Halo and POI) had been subtracted

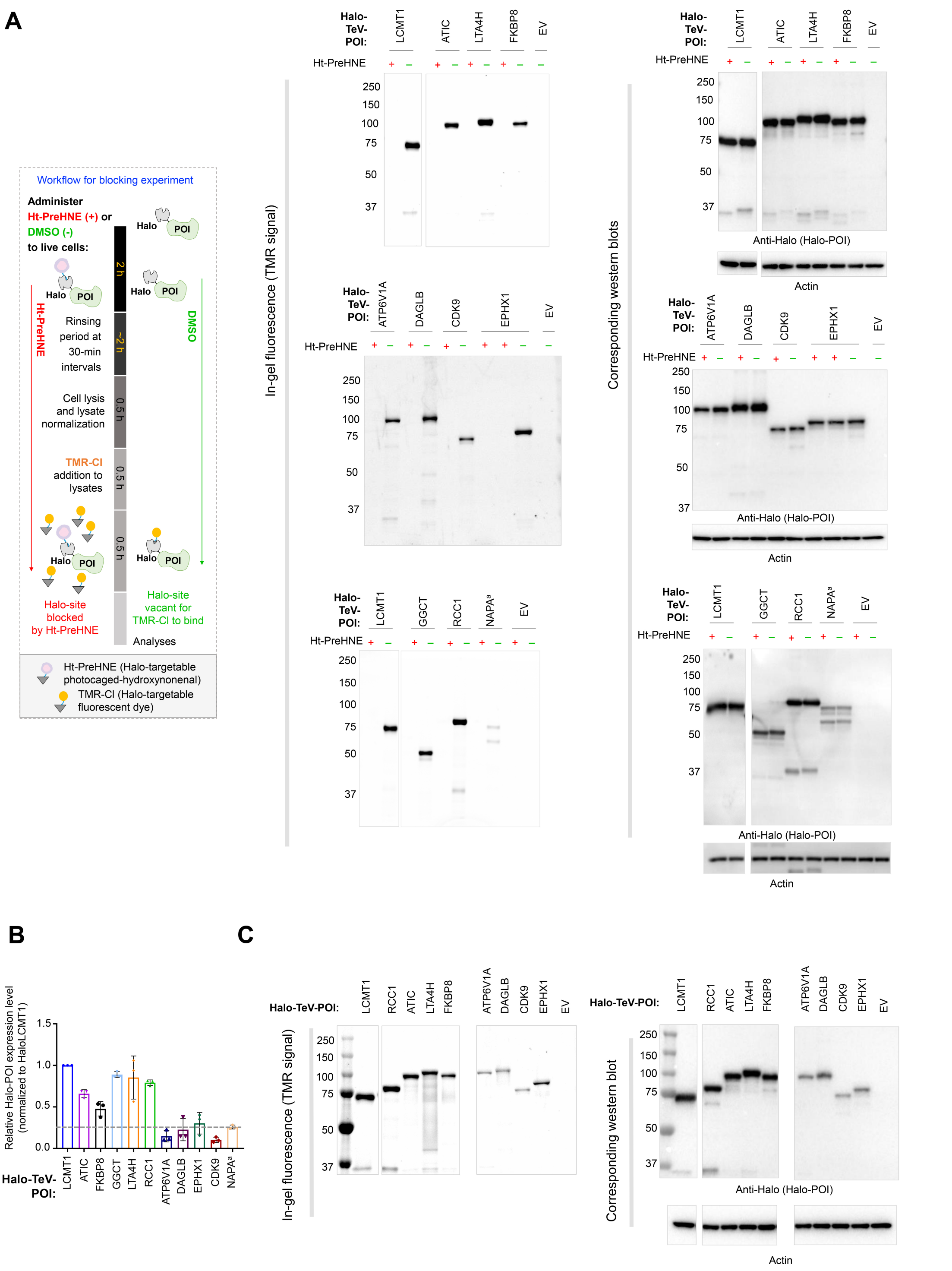

Supplementary Figure 5. Validation of selected proteins of interest (POIs) for Halo-POI-expression and saturation of Ht-PreHNE-binding (to Halo).

Signal from anti-actin was used for loading control in all western blots.

(**A**) Blocking experiments (see ***inset*** for workflow) using Ht-PreHNE and TMR-Cl showed that Ht-PreHNE fully saturates HaloTag for each Halo-POI fusion, regardless of Halo-POI expression level. HEK293T cells ectopically expressing Halo-POI were treated by either Ht-PreHNE (20 μM) or DMSO for 2 h followed by rinsing to remove excess Ht-PreHNE, prior to harvesting. Normalized cell lysates were treated with 3 μM TMR-Cl for 30 min at 37 °C, and analyzed by in-gel fluorescence (detecting TMR-signal, and reporting active HaloTag protein, *left panel*) and western blot (reporting Halo-POI expression, *right panel*). The lower band (around ~33 kDa) with weaker signal in the western blot is HaloTag protein alone, likely resulting from cleavage during lysis. [a]Halo-NAPA was consistently found to be susceptible to cleavage.

(**B**) Quantification of relative expression levels of the indicated Halo-fusion proteins of interest (POIs) determined by western blot analysis using anti-Halo antibodies. (The expression level of Halo-TeV-LCMT1 is set to be 1). (n = 3 biological replicates, error bars indicate s.d.; two-tailed *t*-test was applied). Horizontal dotted line designates cut off where we determined biotin Click was necessary to perform HNE sensing validations by T-REX. [a]Halo-NAPA was consistently found to be susceptible to cleavage.

(**C**) Comparison of Halo-activity (in-gel fluorescence) and Halo-POI protein expression level (western blot) across the indicated Halo-POI hits, respectively reported by Halo-targetable TMR-Cl small-molecule fluorophore and western blot using anti-Halo antibody. Workflow was similar to that shown in the ***inset*** in **A** but after HEK293T cells were harvested, lysed and lysate protein content normalized, lysates were treated with 3 μM TMR-Cl for 30 min at 37 °C then directly analyzed by SDS-PAGE. *Left panel:* in-gel fluorescence analysis (TMR signal); *right panel:* western blot analysis using anti-Halo.

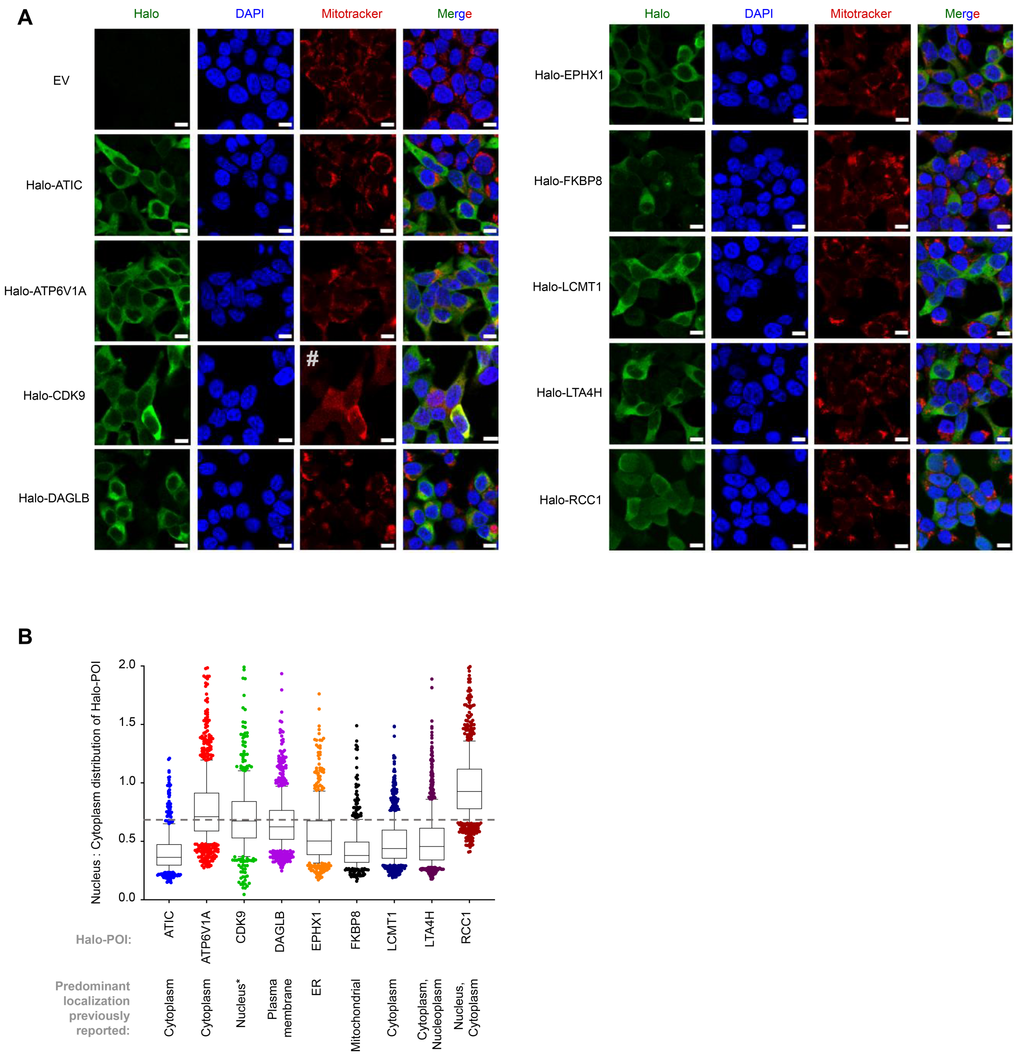

Supplementary Figure 6. **Localization of Halo-POI from the indicated hits**.

(**A**) Representative IF images of HEK293T cells ectopically expressing the indicated Halo-POI. Following transfection, cells were treated with Mitotracker Red CMXRos before fixing with 2% paraformaldehyde in DPBS [except for the sample marked by **#** (where POI = CDK9), in which this mitotracker-staining step was skipped, and IF analysis was performed with the anti-CDK9 antibody (from ProteinTech); thus, the red stain in this panel probes endogenous CDK9]. Thus, for all samples except that marked by **#**, the only antibody used detected Halo (green). Scale bars, 10 μm.

(**B**) Quantification of nucleus:cytoplasm distribution of representative Halo-POI ectopically expressed in HEK293T cells. (n > 450 for all POIs. For box plots, center lines indicate median, box limits are the first and third quartiles and whisker ends represent 10–90% confidence intervals. Data not included between the whiskers are plotted as dots). Horizontal dotted line denotes the point at which we considered nuclear contribution to be significant. Preferred subcellular locales previously reported in the literature for the corresponding POIs are also listed within **Fig.S4**. *For CDK9, catalytically-functional locale is nucleus although presence in the cytoplasm is also widely reported. See discussion in main text.

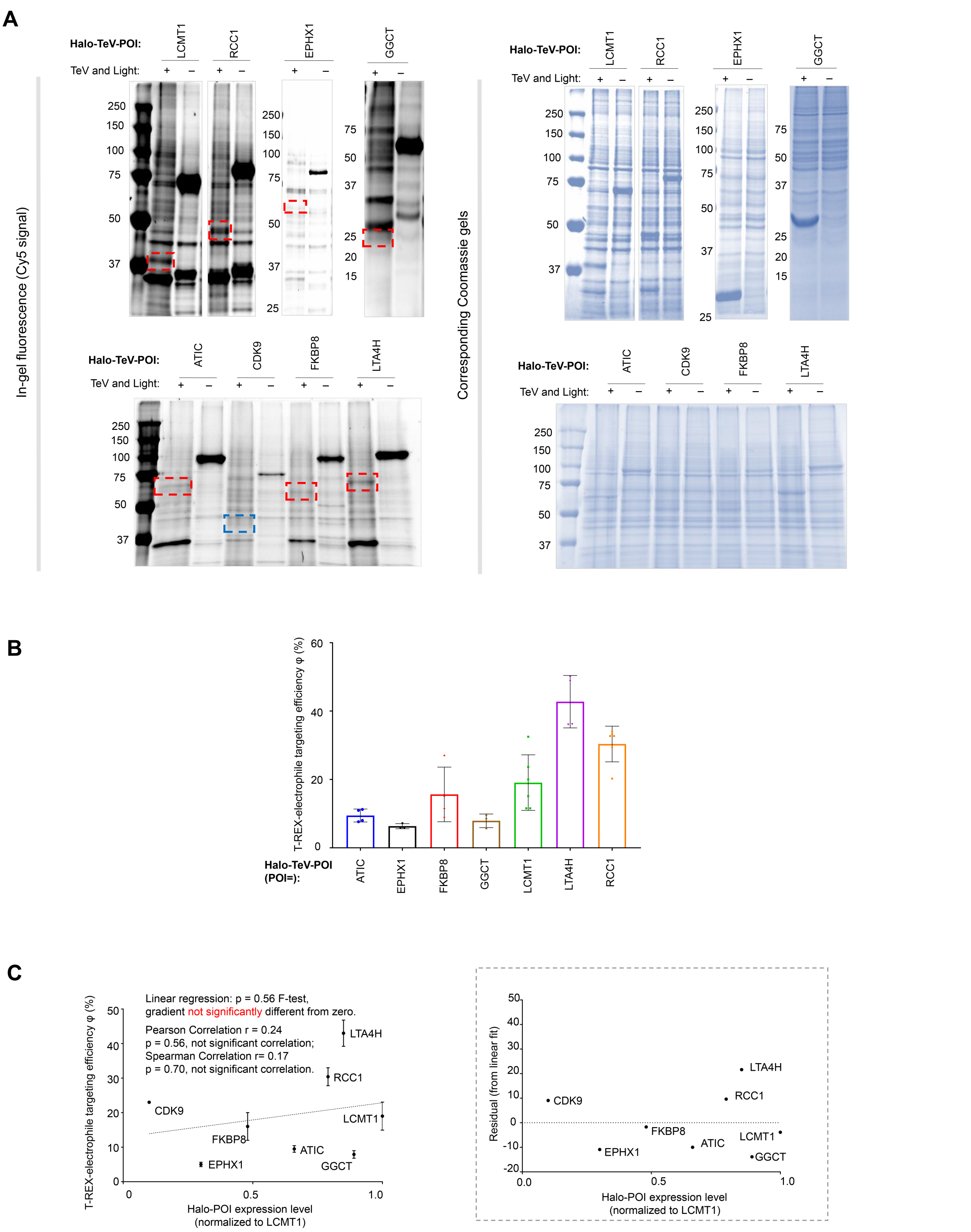

Supplementary Figure 7. **Evaluation of electrophile-sensing ability of specific POI enabled by T-REX (see Fig. S1B).**

(**A**) HEK293T cells were transfected with the specified Halo-TeV-POI construct and treated with Ht-PreHNE (20 μM) for 2 h. Following rinsing of unbound Ht-PreHNE, cells were either illuminated (to trigger T-REX-assisted hydroxynonenal-delivery) or untreated with light (i.e., probe-alone control) (**Fig.S1B-C**). Normalized lysates from these two sets of cells were treated with either TeV-protease (for light-illuminated samples) or buffer alone (for probe-alone control samples). Lysates were then subjected to Click coupling with Cy5-azide and analyzed by in-gel fluorescence. *Left panel:* Cy5 fluorescence signal. Red dashed rectangles mark the region of interest within the Cy5 gel, for each POI (i.e., where the hydroxynonenylated

protein band lies) except for CDK9, where the region of interest is marked in by a blue rectangle (in this instance, as described in **Fig.S4**, CDK9 expression levels are too low to allow us to assign the Cy5-signal confidently using in-gel fluorescence analysis, thus no judgment call was made). *Right panel:* Coomassie staining of the same gels assessing total lysate-protein loading. ***Note***: in gels analyzing EPHX1 and GGCT, the band at ~30 kDa in Coomassie-stained gels exposed to light is TeV protease (~27 kDa)[7], whereas in other gels where this band was not visible, this is because the 25 kDa marker had been run out of the gel (see additional supplemental file for blots/gels in full view).

(**B**) Quantification of T-REX-electrophile targeting efficiency (ϕ) of all POIs except CDK9, from in-gel fluorescence analysis in **Fig.S7A**. (n $\geq$ 3 biological replicates for all bars, error bars indicate s.e.m.; two-tailed *t*-test was applied). For derivation of the value of ϕ, please see **Fig.S4** legend.

(**C**) A plot of T-REX-electrophile targeting efficiency (ϕ) (from **Fig.S7B**) against the relative Halo-POI-expression level (from **Fig.S5C**) for the 8 Halo-POIs for which ϕ was quantitated. ***Note-1*:** uncut gels/blots were included in supplemental file pertaining to full-view gels/blots, allowing relative quantification of bands. ***Note-2*:** the corresponding ligand occupancy for where POI = CDK9 was derived from Click-biotin-pulldown assay (see also **Fig.S4** legend; see **Fig.1C** and discussion in main text). The data were analyzed using linear regression fit. The fit is poor (R^2 = 0.06 for the goodness of linear fit) and shows a no significant deviation from a slope of 0. This indicates *no clear correlation* between Halo-POI expression-level and hydroxynonenal-sensing efficiency (ϕ) was observed. (This observation is expected based on the known working mechanisms underlying T-REX system). Both Pearson r and Spearman r have been computed by GraphPad Prism 8. The r- and p- values for both Pearson r and Spearman r analysis were computed using the means of protein expression level (x-axis) against every protein's targeting efficiency (phi, y-axis), with the assumption that all data followed Gaussian-distribution. The p-values were calculated using a two-tailed students t-test. ***Inset** on right:* Residual of linear regression for the plot of percentage delivery vs. expression.

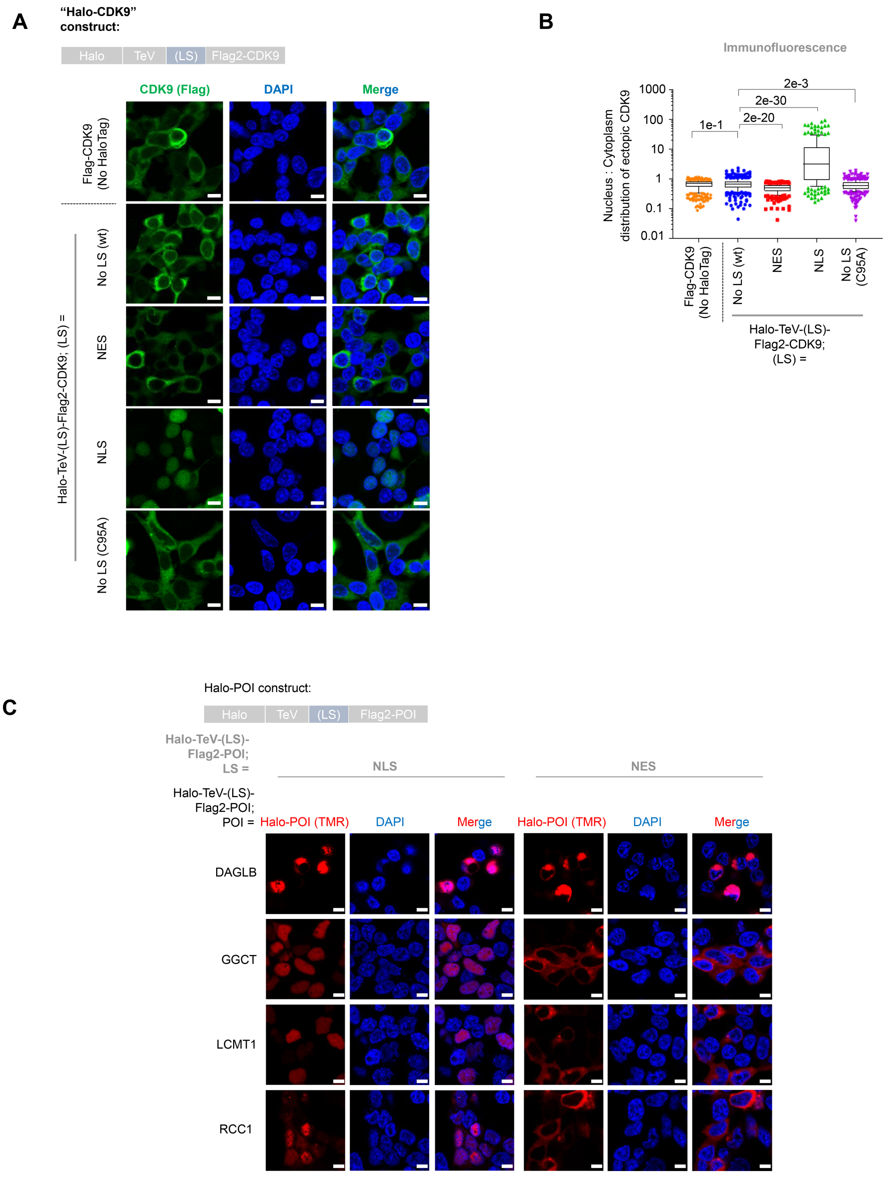

Supplementary Figure 8. **Localization sequence (LS) tags within the indicated Halo-(LS)-TeV-Flag2-POI constructs gave rise to expected nucleus/cytoplasm localization.**

(**A**) HEK293T cells expressing different Halo-(LS)-TeV-Flag2-CDK9s, wherein "LS" designates a specific localization-sequence-tag: untargeted (no LS), nuclear (NLS), or cytoplasmic (NES)-targeted tags, were fixed and analyzed by IF using anti-Flag antibody. DAPI shows the nucleus. Scale bars, 10 µm. For the significance of the images associated with CDK9 functional mutant, 'No LS(C95A)', see later sections of the main text. For functional comparisons between ectopic and endogenous CDK9, see **Fig.S17A, S8A-B**, and **S6A-B**.

(**B**) Quantification of the nucleus:cytoplasm ratio of the indicated CDK9-variant (probing for Flag signal; i.e., transfected CDK9). (***Note***: even Halo-TeV-(NLS)-Flag2-CDK9 has a nucleus:cytoplasm ratio close to 1:1 because of the intrinsic difficulties in quantifying the cytoplasmic fraction in HEK293T cells). (n > 220 for all bars. For box plots, center lines indicate medians, box limits are the first and third quartiles and whisker ends represent 10–90% confidence intervals. Data not included between the whiskers are plotted as dots). For the significance of the data associated with 'No LS(C95A)', see later sections of the main text.

(**C**) Similar to **A**, HEK293T cells were transfected with the indicated construct (Halo-TeV-(LS)-Flag2-POI) (wherein POI = DAGLB, GGCT, LCMT1, and RCC1), but live-cell treatment with TMR-Cl (Halo-targetable TMR dye) (3 μM, 30 min) was performed to trace the location of the indicated fusion protein. DAPI indicates nuclear staining. LS = NLS (*left panel*) and NES (*right panel*) respectively. Scale bars, 10 μm. ***Note***: expression of Halo-DAGLB altered cell morphology and hence the TMR signal showed some abnormal aggregation.

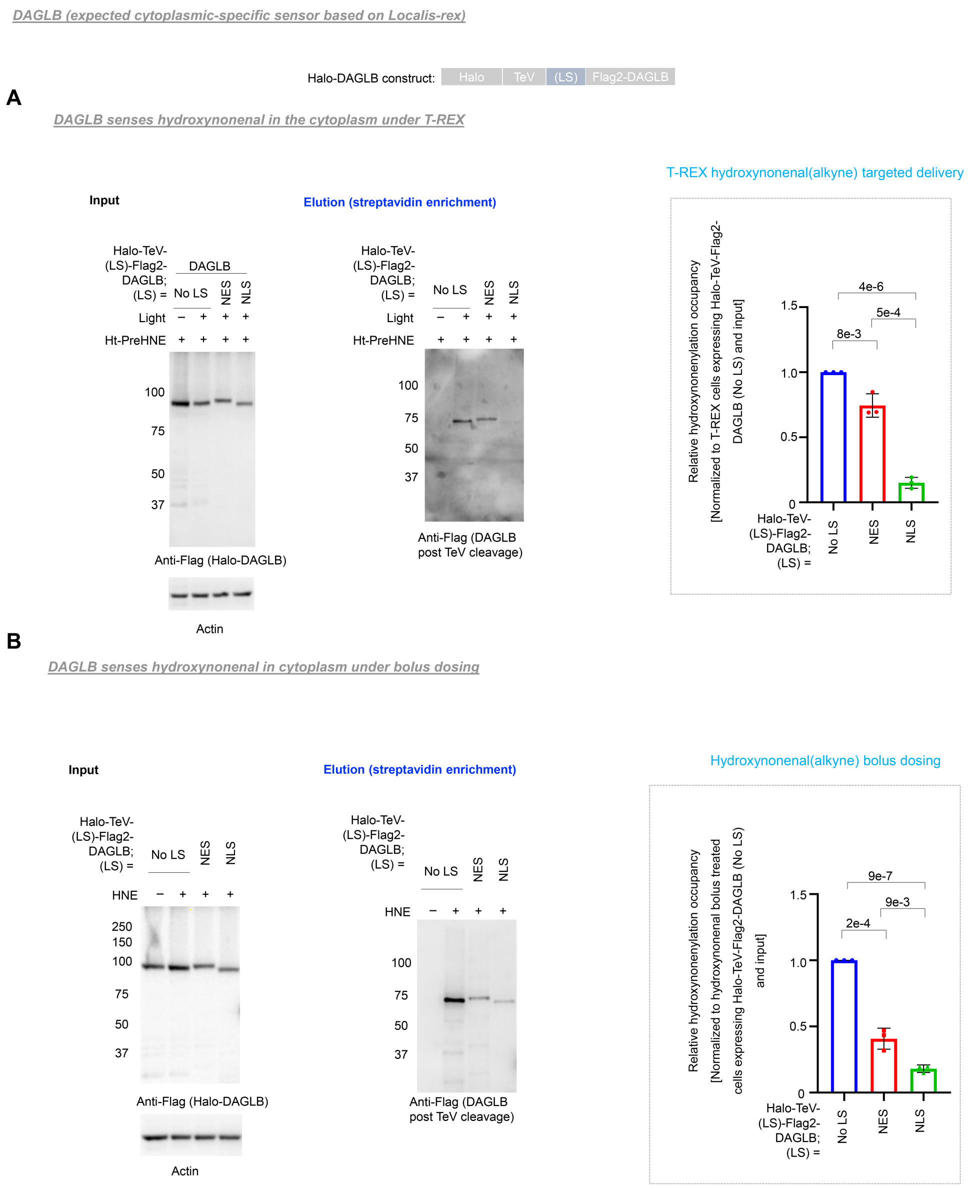

Supplementary Figure 9. **DAGLB senses hydroxynonenal better in the cytoplasm, as predicted from Localis-rex, by either T-REX or bolus HNE dosing**.

(A) HEK293T cells expressing Halo-TeV-(LS)-Flag2-DAGLB were subjected to T-REX (LS designates the localization sequences as described above). Post cell lysis and TeV-cleavage to separate Halo and POI, the extent of hydroxynonenylation on DAGLB was evaluated using Click-biotin-pulldown assay. Representative western blots are shown. ***Inset***: quantification (n = 3 biological replicates, error bars indicate s.d.; two-tailed *t*-test was applied).

(B) Similar to **A**, cells were treated with bolus hydroxynonenal (HNE, alkyne-functionalized) (15 μM, 2 h) before cell lysis, TeV-cleavage to separate Halo and POI, and Click-biotin-pulldown assay to measure the extent of hydroxynonenylation on DAGLB. Representative western blots are shown. ***Inset***: quantification (n = 3 biological replicates, error bars indicate s.d.; two-tailed *t*-test was applied).

Note: input samples in all cases are not treated with TeV protease, whereas elution samples are derived from samples treated with TeV protease. Thus, the molecular weight of input and elution differ.

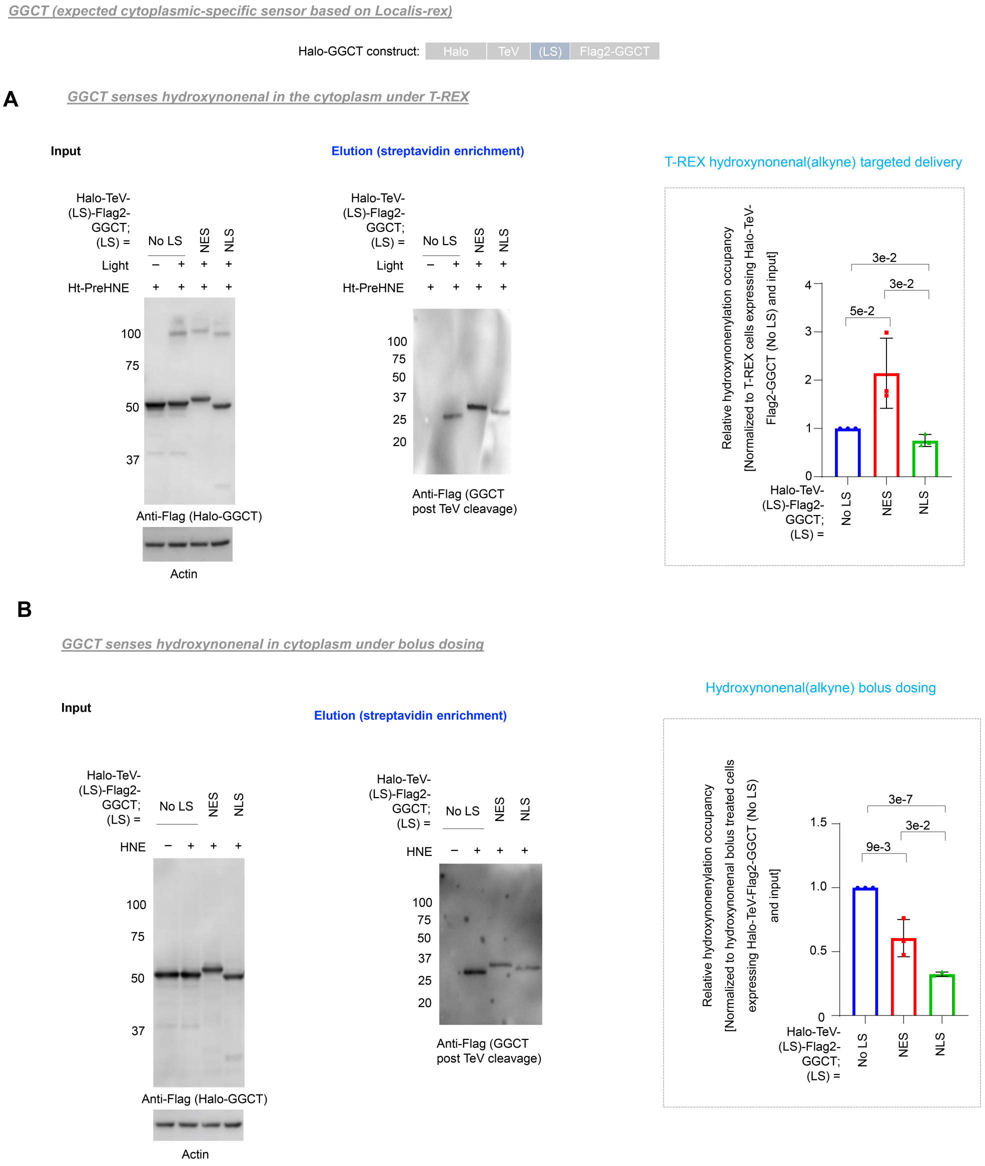

Supplementary Figure 10. **GGCT senses hydroxynonenal better in the cytoplasm, as predicted from Localis-rex, by either T-REX or bolus HNE dosing.**

(A) HEK293T cells expressing Halo-TeV-(LS)-Flag2-GGCT were subjected to T-REX (LS designates the localization sequences as described above). Post cell lysis, and TeV-cleavage that separates Halo from POI, the extent of hydroxynonenylation on GGCT was evaluated using Click-biotin-pulldown assay. Representative western blots are shown. ***Inset***: quantification (n = 3 biological replicates, error bars indicate s.d.; two-tailed *t*-test was applied).

(B) Similar to **A**, cells were treated with bolus hydroxynonenal (HNE, alkyne-functionalized) (15 μM, 2 h) before cell lysis, TeV-cleavage that separates Halo from POI, Click-biotin-pulldown assay to measure the extent of hydroxynonenylation on GGCT. Representative western blots are shown. ***Inset***: quantification n = 3 biological replicates, error bars indicate s.d.; two-tailed *t*-test was applied).

Note: input samples in all cases are not treated with TeV protease, whereas elution samples are derived from samples treated with TeV protease. Thus, the molecular weight of input and elution differ.

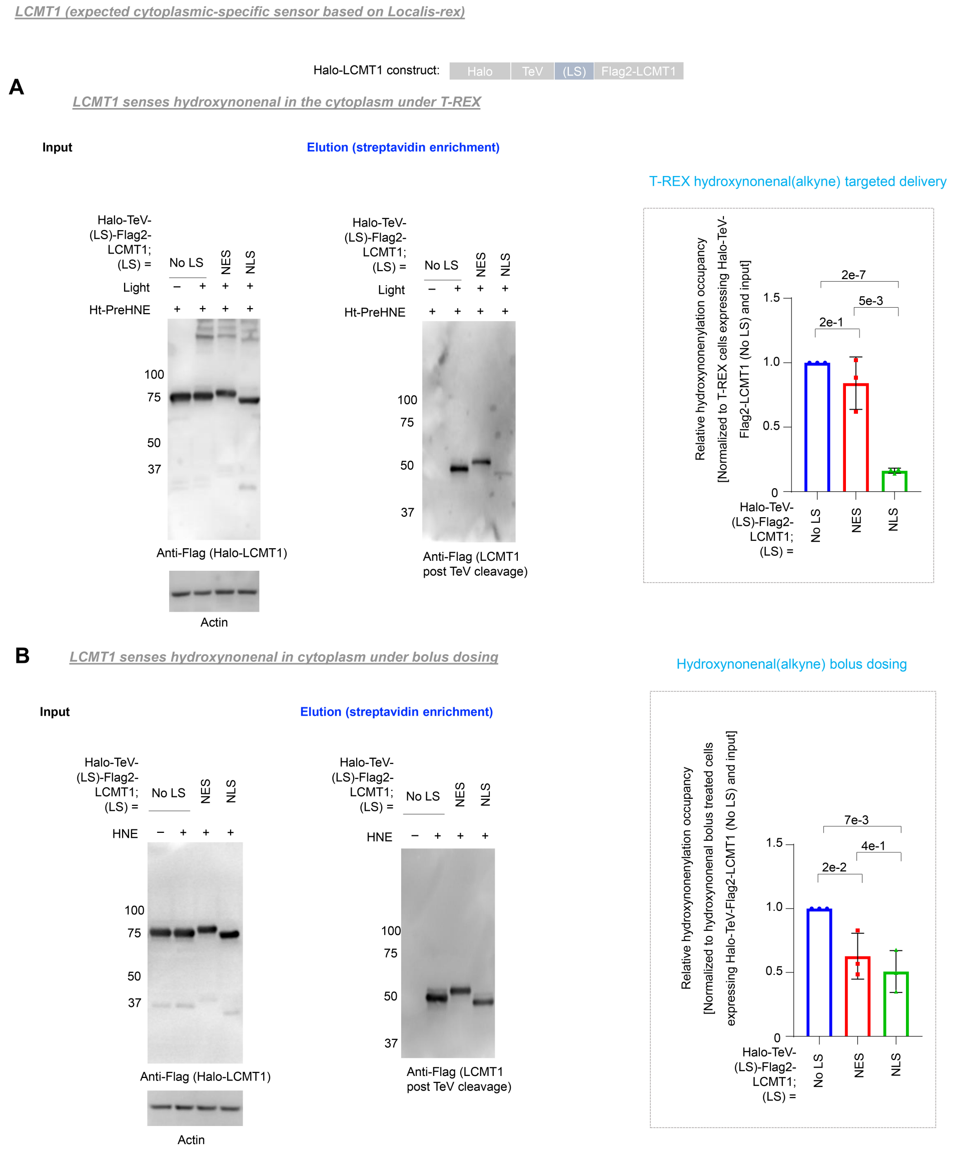

Supplementary Figure 11. **LCMT1 senses hydroxynonenal better in the cytoplasm, as predicted from Localis-rex, by T-REX.**

(A) HEK293T cells expressing Halo-TeV-(LS)-Flag2-LCMT1 were subjected to T-REX (LS designates the localization sequences as described above). Post cell lysis, and TeV-cleavage that separates Halo from POI, the extent of hydroxynonenylation on LCMT1 was evaluated using Click-biotin-pulldown assay. Representative western blots are shown. ***Inset***: quantification (n = 3 biological replicates, error bars indicate s.d.; two-tailed *t*-test was applied).

(B) Similar to **A**, cells were treated with bolus hydroxynonenal (HNE, alkyne-functionalized) (15 μM, 2 h) before cell lysis, TeV-cleavage that separates Halo from POI, and Click-biotin-pulldown assay to measure the extent of hydroxynonenylation on LCMT1. Representative western blots are shown. ***Inset***: quantification (n = 3 biological replicates, error bars indicate s.d.; two-tailed *t*-test was applied).

Note: input samples in all cases are not treated with TeV protease, whereas elution samples are derived from samples treated with TeV protease. Thus, the molecular weight of input and elution differ.

RCC1 (expected cytoplasmic-specific sensor based on Localis-rex):

Halo-RCC1 construct: Halo | TeV | (LS) | Flag2-RCC1

A *RCC1 senses hydroxynonenal in the cytoplasm under T-REX*

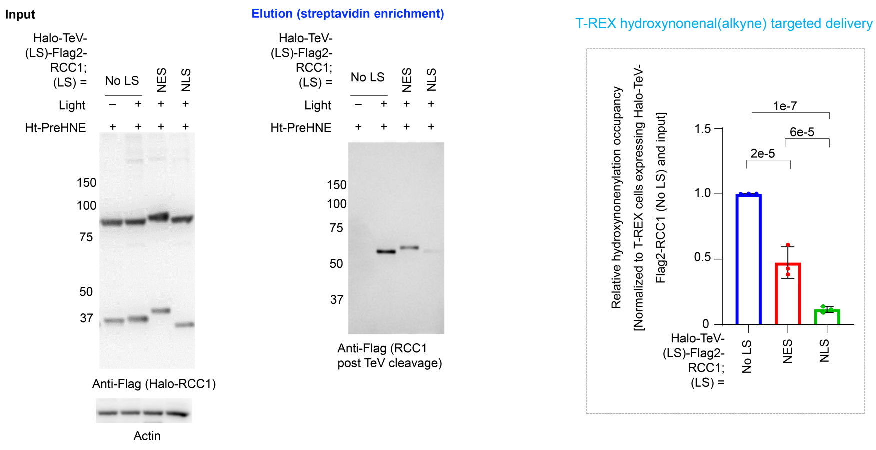

B *RCC1 senses hydroxynonenal in cytoplasm under bolus dosing*

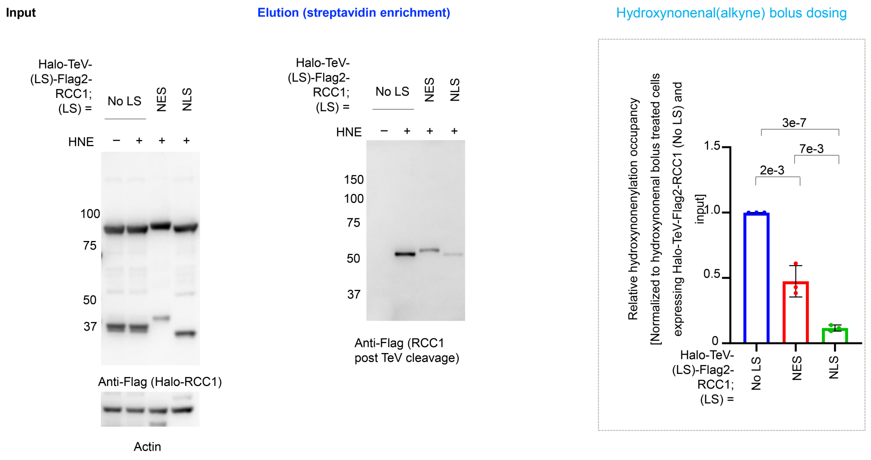

Supplementary Figure 12. RCC1 senses hydroxynonenal better in the cytoplasm, as predicted from Localis-rex, by either T-REX or bolus HNE dosing.

(A) HEK293T cells expressing Halo-TeV-(LS)-Flag2-RCC1 were subjected to T-REX (LS designates the localization sequences as described above). Post cell lysis, and TeV-cleavage that separates Halo from POI, the extent of hydroxynonenylation on RCC1 was evaluated using Click-biotin-pulldown assay. Representative western blots are shown. ***Inset***: quantification (n = 3 biological replicates, error bars indicate s.d.; two-tailed *t*-test was applied).

(B) Similar to **A**, cells were treated with bolus hydroxynonenal (HNE, alkyne-functionalized) (15 µM, 2 h) before cell lysis, TeV-cleavage that separates Halo from POI, and Click-biotin-pulldown assay to measure the extent of hydroxynonenylation on RCC1. Representative western blots are shown. ***Inset***: quantification (n = 3 biological replicates, error bars indicate s.d.; two-tailed *t*-test was applied).

Note: input samples in all cases are not treated with TeV protease, whereas elution samples are derived from samples treated with TeV protease. Thus, the molecular weight of input and elution differ.

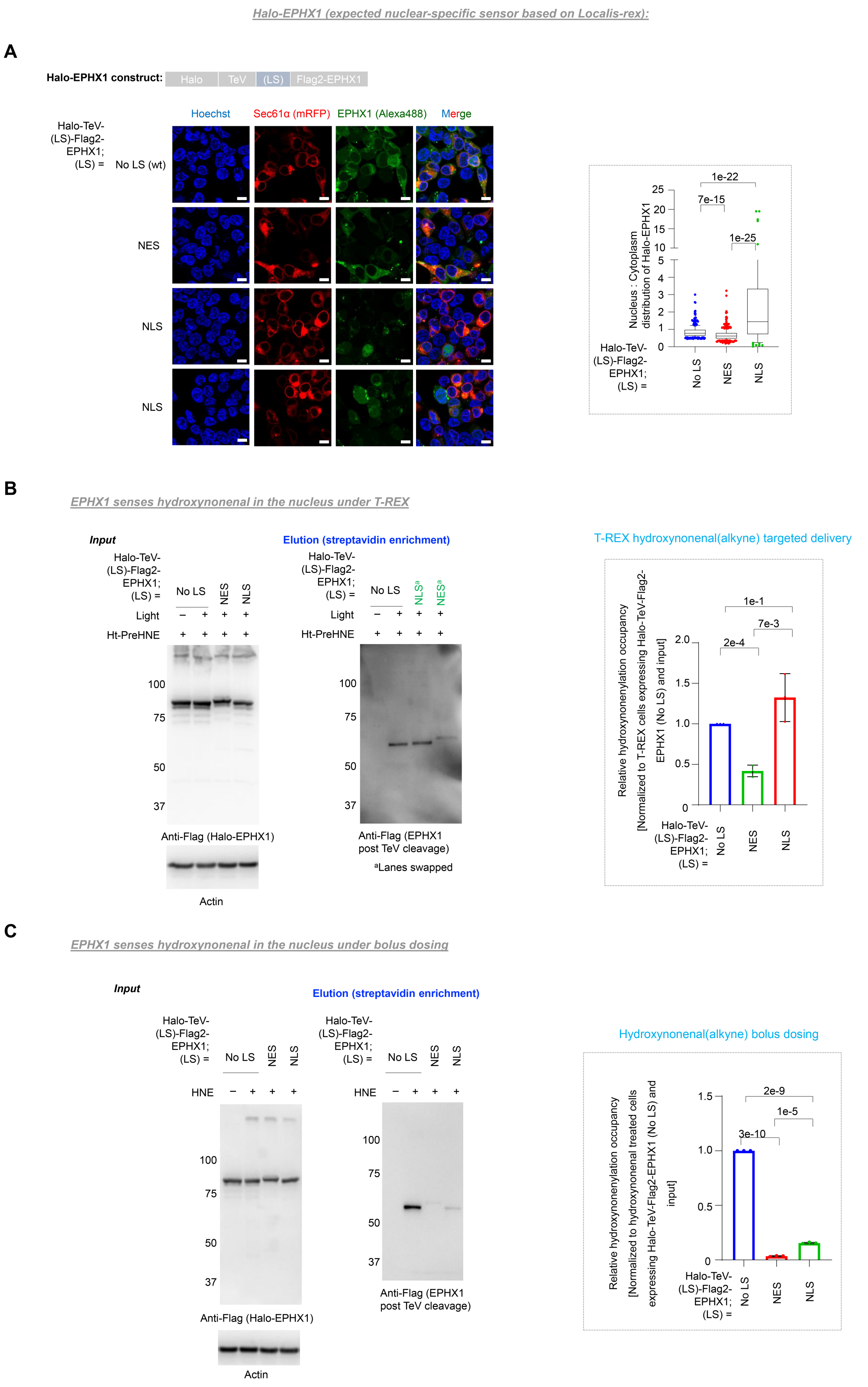
Halo-EPHX1 (expected nuclear-specific sensor based on Localis-rex):
A
Halo-EPHX1 construct: Halo TeV (LS) Flag2-EPHX1
Hoechst
Sec61α (mRFP)
EPHX1 (Alexa488)
Merge
Halo-TeV-(LS)-Flag2-EPHX1; (LS) = No LS (wt)
NES
NLS
NLS
Nucleus : Cytoplasm distribution of Halo-EPHX1
1e-22
7e-15
1e-25
Halo-TeV-(LS)-Flag2-EPHX1; (LS) =
No LS
NES
NLS
B
EPHX1 senses hydroxynonenal in the nucleus under T-REX
Input
Elution (streptavidin enrichment)
Halo-TeV-(LS)-Flag2-EPHX1; (LS) =
No LS
NES
NLS
NLSa
NESa
Light
Ht-PreHNE
Anti-Flag (Halo-EPHX1)
Actin
Anti-Flag (EPHX1 post TeV cleavage)
aLanes swapped
T-REX hydroxynonenal(alkyne) targeted delivery
Relative hydroxynonenylation occupancy [Normalized to T-REX cells expressing Halo-TeV-Flag2-EPHX1 (No LS) and input]
1e-1
7e-3
2e-4
C
EPHX1 senses hydroxynonenal in the nucleus under bolus dosing
Input
Elution (streptavidin enrichment)
HNE
Anti-Flag (Halo-EPHX1)
Actin
Anti-Flag (EPHX1 post TeV cleavage)
Hydroxynonenal(alkyne) bolus dosing
Relative hydroxynonenylation occupancy [Normalized to hydroxynonenal treated cells expressing Halo-TeV-Flag2-EPHX1 (No LS) and input]
2e-9
1e-5
3e-10

Supplementary Figure 13. **EPHX1 senses hydroxynonenal better in the nucleus, as predicted from Localis-rex, by either T-REX or bolus HNE dosing.**

(A) HEK293T cells expressing Halo-TeV-(LS)-Flag2-EPHX1 (LS designates the localization sequences as described above), and Sec61α-mRFP (an ER marker), were treated with Alexa488-Cl (that specifically and covalently binds to Halo, similar to TMR-Cl used above (**Fig.S8C**) and is chromatically orthogonal to mRFP) for 2 h at 37 °C. After rinsing to remove excess Alexa488-Cl, the live cells were analyzed using confocal microscopy. *Left panel*: Representative cell images are shown. Scale bars, 10 μm; ***Inset***: quantification of nucleus:cytoplasm ratios for Halo-TeV-(LS)-Flag2-EPHX1 bearing different LS (Mean ± s.e.m. are presented; (n > 70 for each bar). For box plots, center lines indicate medians, box limits are the first and third quartiles and whisker ends represent 10–90% confidence intervals. Data not included between the whiskers are plotted as dots).

(B) HEK293T cells expressing Halo-TeV-(LS)-Flag2-EPHX1 were subjected to T-REX. Post cell lysis, and TeV-cleavage that separates Halo and POI, the extent of hydroxynonenylation on EPHX1 was evaluated using Click-biotin-pulldown assay. Representative western blots are shown. ***Inset***: quantification (n = 3 biological replicates, error bars indicate s.d.; two-tailed *t*-test was applied). ***Note***[a]**:** in the elution blot lanes representing NES and NLS samples were swapped relative to the input gel (this is also apparent from a relative shift in the molecular weight of the respective gel bands).

(C) Similar to **A**, the cells were treated with bolus hydroxynonenal (HNE, alkyne-functionalized) (15 μM, 2 h) before cell lysis, TeV-cleavage that separates Halo and POI, and Click-biotin-pulldown assay, to measure the extent of hydroxynonenylation on EPHX1. Representative western blots are shown. ***Inset***: quantification (n = 3 biological replicates, error bars indicate s.d.; two-tailed *t*-test was applied).

Note: input samples in all cases are not treated with TeV protease, whereas elution samples are derived from samples treated with TeV protease. Thus, the molecular weight of input and elution differ.

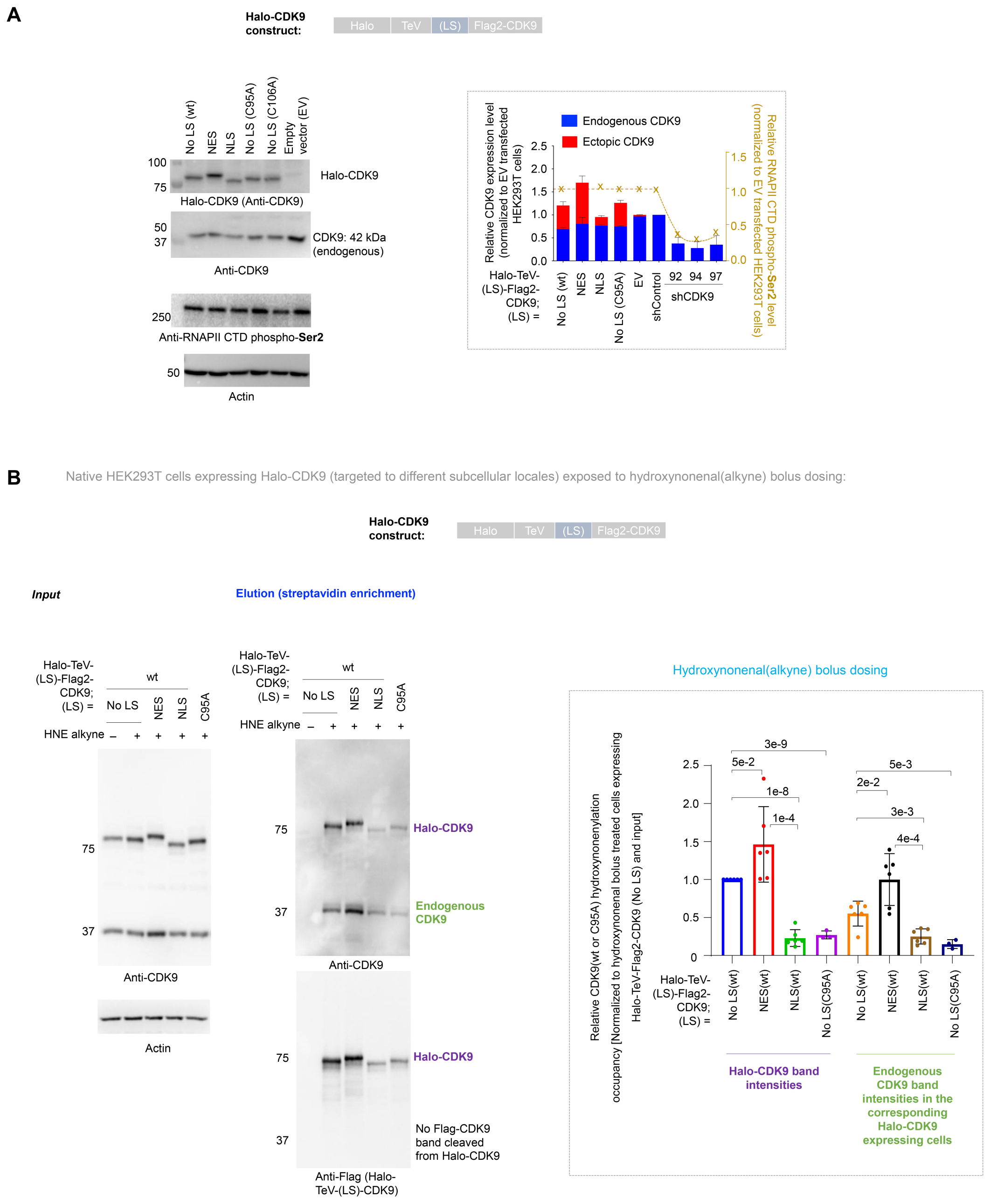

Supplementary Figure 14. **Ectopic Halo-TeV-Flag2-CDK9 is functionally similar to endogenous CDK9; CDK9 is a context-specific sensor under whole-cell hydroxynonenal treatment**.

(**A**) HEK293T cells were transfected with the indicated constructs Halo-TeV-(LS)-Flag2-CDK9, where “LS” designates a specific localization-sequence-tag as indicated above. Levels of CDK9 (LS-tagged or No-LS-tagged as indicated) were analyzed by western blot using anti-CDK9 antibodies. (***Note***: CDK9 transgene is HaloTagged (~75 kDa), and is thus readily resolvable from endogenous CDK9 (~37 kDa) on SDS-PAGE). Representative western blots are shown. ***Inset***: quantification of the amount of CDK9 [both endogenous (**blue**) and ectopic (**red**) CDK9, in transiently-transfected cells (or shRNA-knockdown cells; discussed in later sections)] derived from the western-blot analysis. *The top blot (anti-CDK9 detecting Halo-CDK9) and second to top blots (anti-CDK9 detecting endogenous CDK9) are derived from the same blot that is shown in entirety in the supplemental file containing blots/gels in full view.* **Note**: for subsequent

discussions related to **Fig.S17A** and **Fig.S8A-B**: the brown dotted line with crosses designates the corresponding RNAPII CTD phospho-*Ser2* levels in each case (probed by anti-RNAPII CTD phospho-*Ser2* antibody), relative to either empty vector (EV) transfection or shControl line.

(**B**) HEK293T cells expressing Halo-TeV-(LS)-Flag2-CDK9 (wt) (LS indicates localization sequence: NES, nuclear exclusion sequence; NLS, nuclear localization sequence; no LS, no localization sequence) were treated with DMSO or hydroxynonenal-alkyne (15 μM, 2 h) and the extent of CDK9-hydroxynonenal-modification was assayed post-Click-biotin-pulldown. ***Note-1*****:** *no* TeV cleavage was performed in these lines. ***Note-2*****:** Significance of data associated with C95A is discussed in later sections. Representative western blots are shown. ***Inset***: quantification of hydroxynonenylation of both Halo-CDK9-variants and endogenous CDK9 in the corresponding set, using both input and elution blots probed with anti-CDK9 antibody. (n = 6 biological replicates, error bars indicate s.d.; two-tailed *t*-test was applied). ***Note-3*****:** a corresponding blot probing anti-Flag-signal is included to show that there is no Flag-CDK9 band cleavage from Halo-TeV-(LS)-CDK9, showing that the lower band due to endogenous CDK9 protein (see construct in the top of the figure).

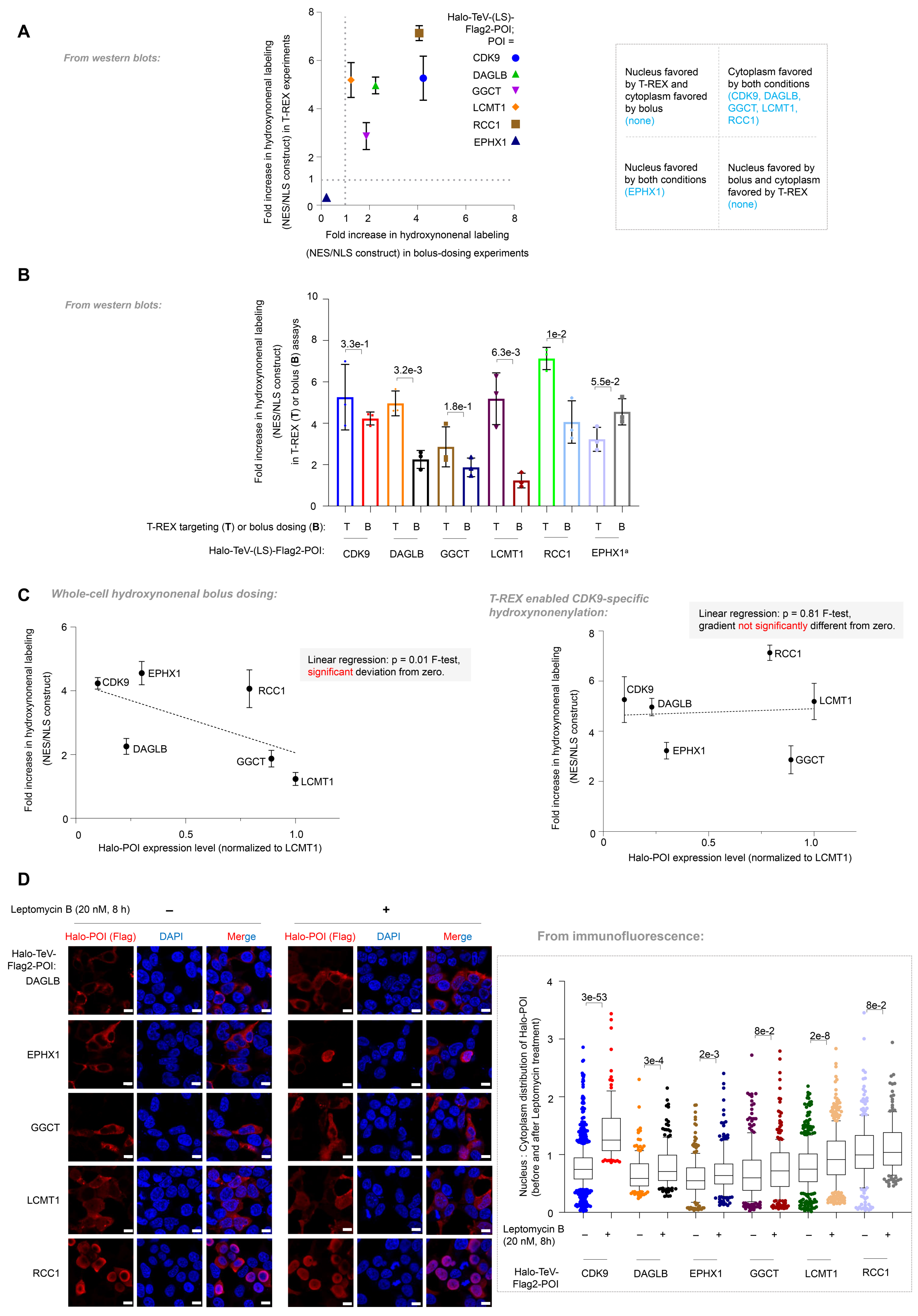
A
From western blots:
Fold increase in hydroxynonenal labeling (NES/NLS construct) in T-REX experiments
Fold increase in hydroxynonenal labeling (NES/NLS construct) in bolus-dosing experiments
Halo-TeV-(LS)-Flag2-POI; POI =
CDK9
DAGLB
GGCT
LCMT1
RCC1
EPHX1
Nucleus favored by T-REX and cytoplasm favored by bolus (none)
Cytoplasm favored by both conditions (CDK9, DAGLB, GGCT, LCMT1, RCC1)
Nucleus favored by both conditions (EPHX1)
Nucleus favored by bolus and cytoplasm favored by T-REX (none)
B
From western blots:
Fold increase in hydroxynonenal labeling (NES/NLS construct) in T-REX (T) or bolus (B) assays
3.3e-1
3.2e-3
1.8e-1
6.3e-3
1e-2
5.5e-2
T-REX targeting (T) or bolus dosing (B):
Halo-TeV-(LS)-Flag2-POI:
CDK9
DAGLB
GGCT
LCMT1
RCC1
EPHX1[a]
C
Whole-cell hydroxynonenal bolus dosing:
Fold increase in hydroxynonenal labeling (NES/NLS construct)
Linear regression: p = 0.01 F-test, significant deviation from zero.
Halo-POI expression level (normalized to LCMT1)
T-REX enabled CDK9-specific hydroxynonenylation:
Linear regression: p = 0.81 F-test, gradient not significantly different from zero.
D
Leptomycin B (20 nM, 8 h)
Halo-POI (Flag)
DAPI
Merge
Halo-TeV-Flag2-POI:
From immunofluorescence:
Nucleus : Cytoplasm distribution of Halo-POI (before and after Leptomycin treatment)
3e-53
3e-4
2e-3
8e-2
2e-8
8e-2
Leptomycin B (20 nM, 8h)
Halo-TeV-Flag2-POI

Supplementary Figure 15. **Results from T-REX-targeted hydroxynonenal delivery and bolus-dosing with hydroxynonenal both *broadly* correlate with data from *Localis-rex***

(A) The fold-difference in the extent of hydroxynonenylation for NES- over NLS-tagged POI constructs under T-REX vs. bolus hydroxynonenal dosing conditions, was analyzed by dividing the extent of protein labeling for Halo-TeV-NES-POI by that for Halo-TeV-NLS-POI in either T-REX or hydroxynonenal-alkyne bolus-dosing conditions, respectively. See **S9,S10,S11,S12,S13B,S14B** for representative blots. For example, the values for the fold-increase in hydroxynonenylation between NES/NLS for CDK9, i.e., the sensing ability for (Halo-TeV-NES-RCC1) / (Halo-TeV-NLS-RCC1), under T-REX (**Fig. S12A**) and bolus (**Fig. S12B**), are respectively 0.482/0.068 = 7.09 and 0.475/0.117 = 4.06. The plot is divided into four quartiles, by the dotted lines starting from 1 from both axes (n = 3 biological replicates, error bars indicate s.e.m.). ***Inset***: explanation of each quartile.

(B) Similar to **A**, fold-increase for each individual POI examined was plotted (n = 3 biological replicates, error bars indicate s.e.m; two-tailed *t*-test was applied). ***Note[a]*:** for EPHX1, the calculation for the fold increase has been reciprocated, i.e., fold-increase = the sensing ability for (Halo-TeV-NLS-EPHX1) / (Halo-TeV-NES-EPHX1). **T** denotes T-REX targeting; **B** denotes hydroxynonenal-bolus dosing.

(C) Analysis of the relationship between Halo-POI expression level against fold-increase between NES/NLS under hydroxynonenal bolus dosing (*left panel*) or T-REX (*right panel*) conditions. ***Note*:** For EPHX1, the ratio of NLS/NES (instead of NES/NLS used for all others) was presented in both graphs. Mean ± s.e.m. are presented in the plot (n = 3 independent biological replicates).

(D) HEK293T cells expressing 6 different Halo-TeV-Flag2-POIs (DAGLB, EPHX1, GGCT, LCMT1, and RCC1) individually were treated with either Leptomycin B (20 nM, 8 h) or an equal volume of solvent, followed by cell fixation using 2% PFA in PBS. Nucleus:cytoplasm ratios were measured by IF analysis detecting anti-Flag-signal. Representative IF images are shown. Scale bars, 10 µm. ***Inset***: quantitation. (n > 150 for each bar. For box plots, center lines indicate medians box limits are the first and third quartiles and whisker ends represent 10–90% confidence intervals. Data not included between the whiskers are plotted dots). Data associated with POI=CDK9 (see **Fig.S16**) are included in this quantification for comparison.

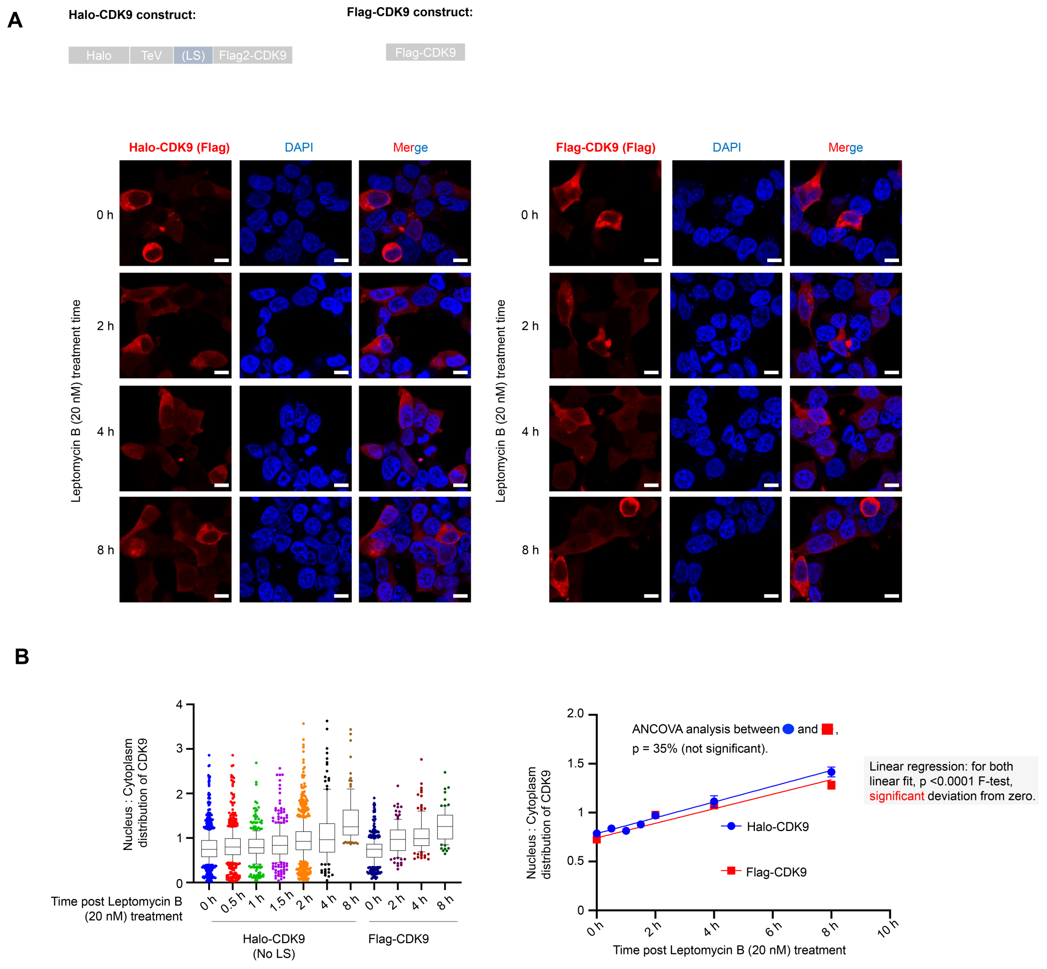

Supplementary Figure 16. **HaloTag does not impair nucleocytoplasmic-shuttling ability of CDK9.**

(A) HEK293T cells expressing Halo-TeV-Flag2-CDK9 or Flag-CDK9 were treated with Leptomycin B (20 nM) for the indicated periods. Cells were then fixed with 2% paraformaldehyde (PFA) in PBS, followed by IF analysis using anti-Flag-antibodies. Representative images are shown. Scale bars, 10 μm.

(B) Quantification. *Left panel*: nucleus:cytoplasm ratios at different treatment periods, for either Halo-TeV-Flag2-CDK9 or Flag-CDK9 (n > 80 for each bar). For box plots, center lines indicate medians; box limits are the first and third quartiles and whisker ends represent 10–90% confidence intervals. Data not included between the whiskers are plotted as dots); *Right panel*: Time-course of CDK9 [either Halo-tagged (●) or non-Halo-tagged (■)] nucleus:cytoplasm ratios following Leptomycin B treatment, analyzed by the following best-fit linear regressions, Y = mX + c, wherein Y, m, X, and c respectively represent the ratios; slope the linear regression fit; time post Leptomycin B treatment (h); and y-axis intercept. For (●): best fit: Y = 0.08X+0.78; for (■): best fit: Y = 0.07X+0.74. Both linear slopes are significantly non-zero (p<0.0001 by F-test).

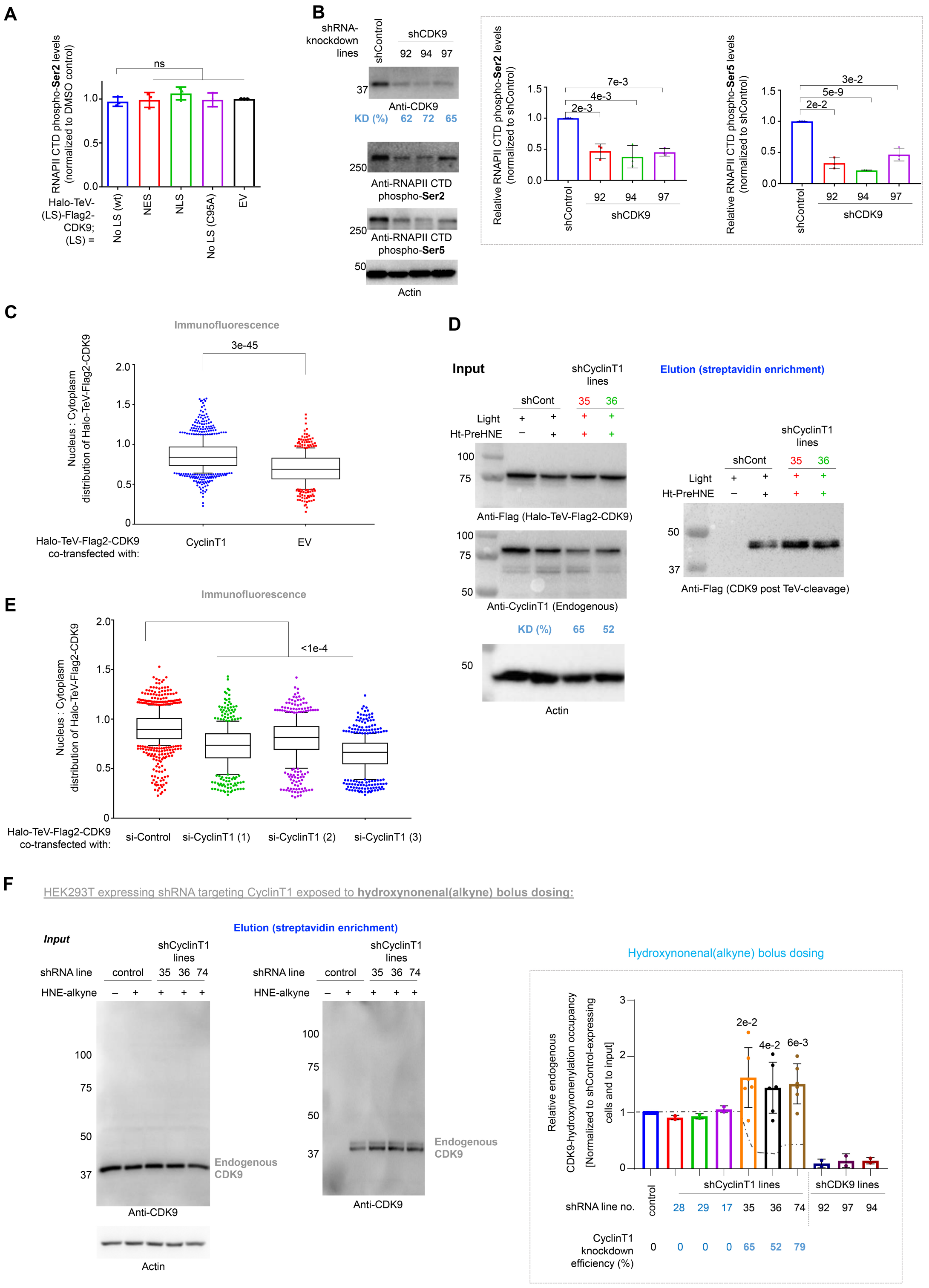

Supplementary Figure 17. **CyclinT1 suppresses the electrophile sensitivity of CDK9 through competitive inhibition under both T-REX and whole-cell bolus dosing conditions**.

(A) Quantification (of data in **Fig.S14A**) RNAPII CTD phospho-*Ser2* levels in cells transiently transfected with the indicated constructs (LS-tagged or No-LS-tagged as indicated, n = 3 biological replicates, error bars indicate s.d.).

(**B**) HEK293T cells were infected with lentiviruses delivering CDK9-specific shRNAs. Three different polyclonal knockdown lines were created, each expressing a specific shRNA targeting CDK9 or a control line expressing a non-targeted shRNA. Representative western blot analyzing the whole-cell lysates using the indicated antibody. ***Inset*:** quantification of RNAPII CTD phospho-*Ser*2 and -*Ser*5 based on western blot data (n = 3 biological replicates, error bars indicate s.d.). KD (%) designates percentage knockdown efficiencies in each knockdown line; 92, 94, and 97 designate the ID of individual independent knockdown lines expressing different CDK9-specific shRNAs (Supplementary Table S2).

(**C**) HEK293T cells were transfected with a plasmid encoding Halo-Tev-Flag2-CDK9 and empty vector (EV) or a plasmid encoding HA-CyclinT1. Nucleus:cytoplasm ratios of Halo-Tev-Flag2-CDK9 were assessed by IF using Flag-signal (n > 400 for each bar. For box plots, center lines indicate medians, box limits are the first and third quartiles and whisker ends represent 10–90% confidence intervals. Data not included between the whiskers are plotted as dots).

(**D**) HEK293T cells were infected separately with two different lentiviruses delivering a specific CyclinT1-specific shRNA. (35 and 36 indicate two independent knockdown lines expressing different CyclinT1-specific shRNAs). The extent of hydroxynonenylation on CDK9 in indicated lines was assessed following T-REX by Click-biotin-pulldown analysis. See also **Fig.2C** for quantitation. KD (%) designates percentage knockdown efficiencies in each knockdown line; 35 and 36 designate the ID of individual independent knockdown lines expressing different CyclinT1-specific shRNAs.

(E) HEK293T cells were transfected with Halo-TeV-Flag2-CDK9 and one of the three different siRNAs targeting CyclinT1 (or control siRNA) for 24 h prior to IF analysis probing Flag-signal. Nucleus:cytoplasm ratios of Halo-TeV-Flag2-CDK9 were quantified (n > 400 for each bar. For box plots, center lines indicate medians, box limits are the first and third quartiles and whisker ends represent 10–90% confidence intervals. Data not included between the whiskers are plotted dots).

(F) HEK293T cells were infected with lentivirus delivering CyclinT1-specific shRNA or a non-targeting-shRNA (shControl). Relative hydroxynonenal-occupancy on *endogenous* CDK9 was evaluated by Click-biotin-pulldown assay and western blot analysis, following hydroxynonenal (HNE, alkyne-functionalized) bolus dosing (15 μM, 2 h) in 6 different sh(CyclinT1) lines (each expressing a different shRNA sequence targeting CyclinT1, only *three* of which, 35, 36, 74, showed knockdown of CyclinT1) [or sh(Control) line]. Labeling was also attempted in independent CDK9-knockdown lines. Representative western blots are shown. ***Inset***: quantification of hydroxynonenylation of endogenous CDK9. The grey dotted line designates the corresponding CyclinT1 level in each case, relative to shControl line. n = 6 biological replicates for shControl; n = 6 biological replicates for shCyclinT1-35, -36, and -74 (which show 65-79% CyclinT1-knockdown); n = 2 biological replicates for "shCyclinT1"-28, -29, and -17 (which show 0% CyclinT1-knockdown); and shCDK9-92, -97, and -94 (which show 62-72% CDK9-knockdown, Figure **S17B**). Error bars indicate s.d.; two-tailed *t*-test was applied.

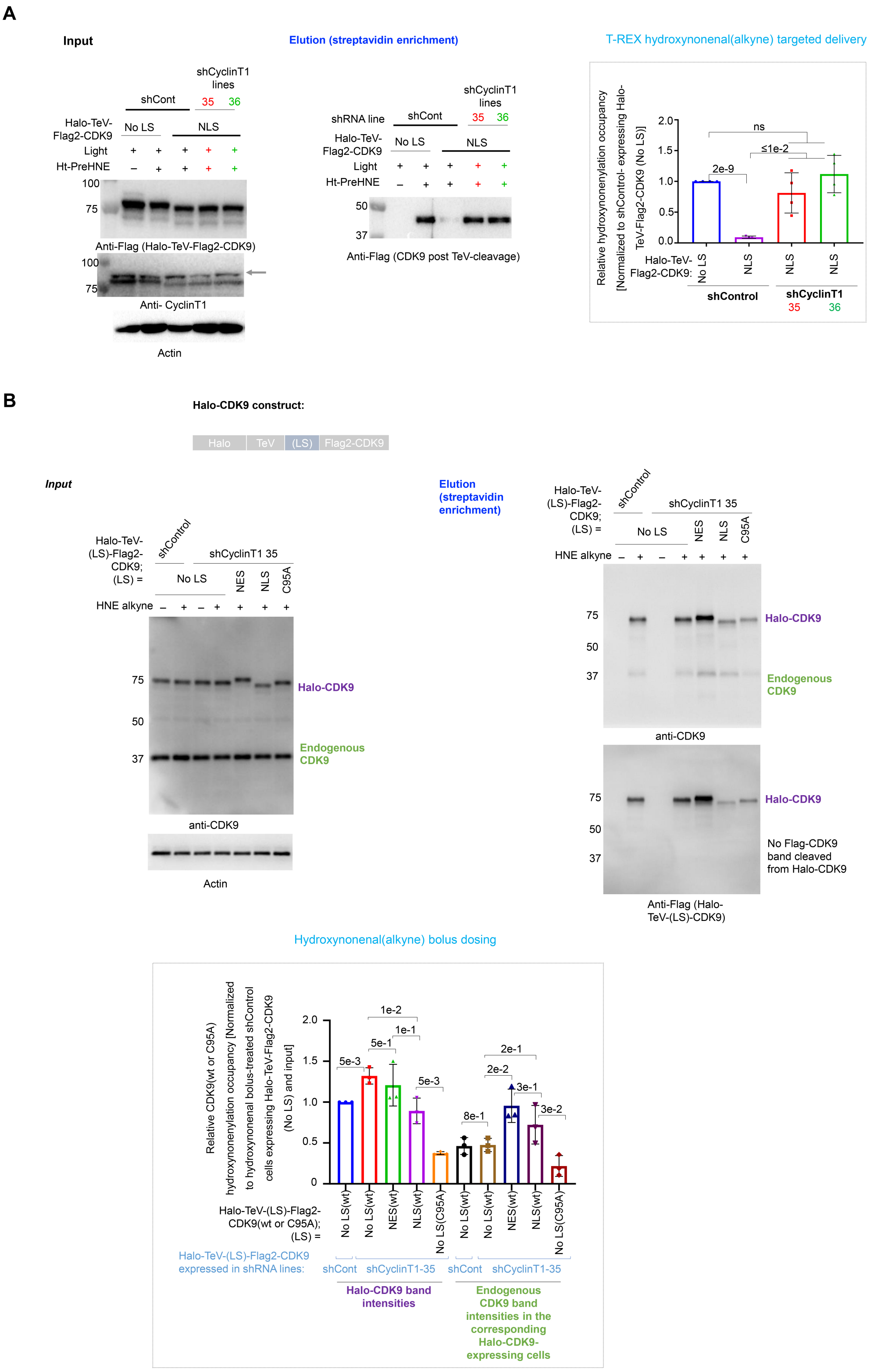

Supplementary Figure 18. **CyclinT1 suppresses the electrophile sensitivity of CDK9 through a combination of alterations in subcellular locale and competitive inhibition, under both T-REX and whole-cell bolus hydroxynonenal dosing conditions**.

(A) HEK293T cells were infected with two different lentiviruses delivering a specific CyclinT1-specific shRNA. (35 and 36 indicate two independent knockdown lines expressing different CyclinT1-specific

shRNAs). The resulting knockdown lines and knockdown controls (shCont) were subsequently transfected with either Halo-TeV-Flag2-CDK9 (No LS) or Halo-TeV-(NLS)-Flag2-CDK9. Following T-REX assay, the extent of hydroxynonenylation on CDK9 was evaluated using Click-biotin-pulldown assay. ***Inset***: quantification (n = 4 biological replicates, error bars indicate s.d.; two-tailed *t*-test was applied). Arrow indicates the band corresponding to CyclinT1 molecular weight and used in quantitation.

(B) HEK293T cells, infected with lentivirus delivering CyclinT1-specific shRNA (shCyclinT1-35) or a non-targeting-shRNA (shControl), expressing Halo-TeV-(LS)-Flag2-CDK9 [wt (no LS, NES, or NLS) or C95A mutant] were treated with DMSO or hydroxynonenal (HNE, alkyne-functionalized) (15 μM, 2 h) and the extent of CDK9-hydroxynonenal-modification was accessed post-Click-biotin-pulldown. Representative western blots are shown for both input and elution of the pulldown. ***Note-1*:** Significance of C95A is discussed in later sections of the manuscript. ***Inset:*** Quantification of hydroxynonenylation of Halo-TeV-(LS)-Flag2-CDK9 [wt (no LS, NES, or NLS) or C95A mutant] in the cells, using both input and elution blots probed with anti-CDK9 antibody. (n = 3 biological replicates, error bars indicate s.d.; two-tailed *t*-test was applied). ***Note-2*:** a corresponding blot probing anti-Flag-signal is included to show that there is no Flag-CDK9 band cleavage from Halo-TeV-(LS)-CDK9, meaning that the lower band in the CDK9 western blot is due to endogenous protein (see construct in the figure).

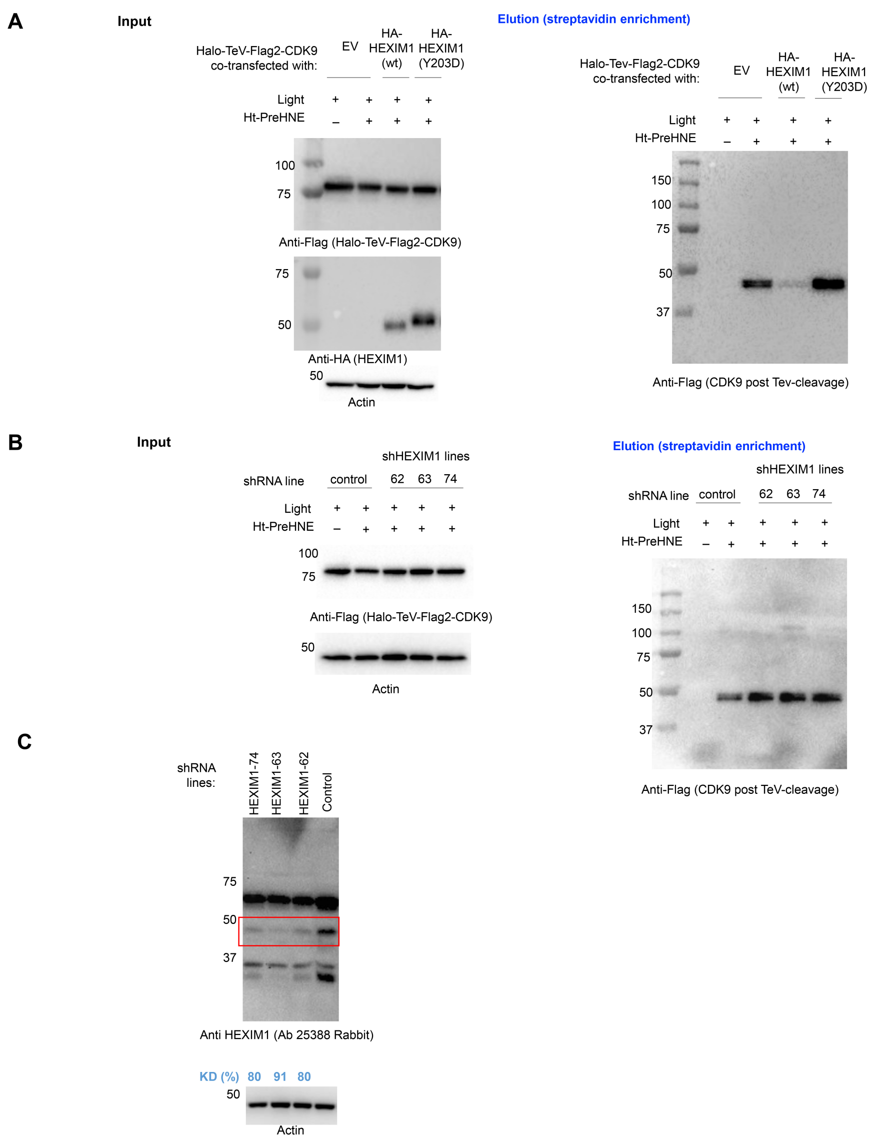

Supplementary Figure 19. HEXIM1 negatively regulates electrophile-sensing propensity of CDK9.

(**A**) HEK293T cells were transfected with indicated plasmids. Following T-REX, cells were lysed, and the extent of hydroxynonenylation on CDK9 was evaluated using Click-biotin-pulldown assay. *Left panel:* input probed by indicated antibodies. *Right panel:* Elution of hydroxynonenylated protein probed by anti-Flag antibodies. ***Note*:** Y203D-HA-HEXIM1 is expressed at noticeably higher levels than the corresponding wt; however, this increased expression of the mutant is biased *against* our conclusion that wt-HEXIM1-overexpression suppresses CDK9-hydroxynonenylation whereas the mutant does not. See **Fig.2D** for quantitation.

(**B**) Similar T-REX experiments to those described in **A** but performed in HEXIM1-knockdown and knockdown-control cells ectopically expressing Halo-TeV-Flag2-CDK9. *Left panel:* input probed by indicated antibodies. *Right panel:* Elution of hydroxynonenylated protein probed by anti-Flag antibodies. See **Fig.2E** for quantitation.

(**C**) Representative blots evaluating HEXIM1-knockdown lines. Red-box indicates the expected band based on HEXIM1 molecular weight. Knockdown (KD) efficiencies are shown as percentages of the control. Rabbit polyclonal anti-HEXIM1 antibody (ab25388) was used in this case. Supplemental full-view gels/blots file shows parallel assessment of the same knockdown lines using sheep polyclonal anti-HEXIM1 antibody (ab28016). KD (%) designates percentage knockdown efficiencies in each knockdown line; 74, 63, and 62 designate the identification no. of individual independent knockdown lines expressing different HEXIM1-specific shRNAs.

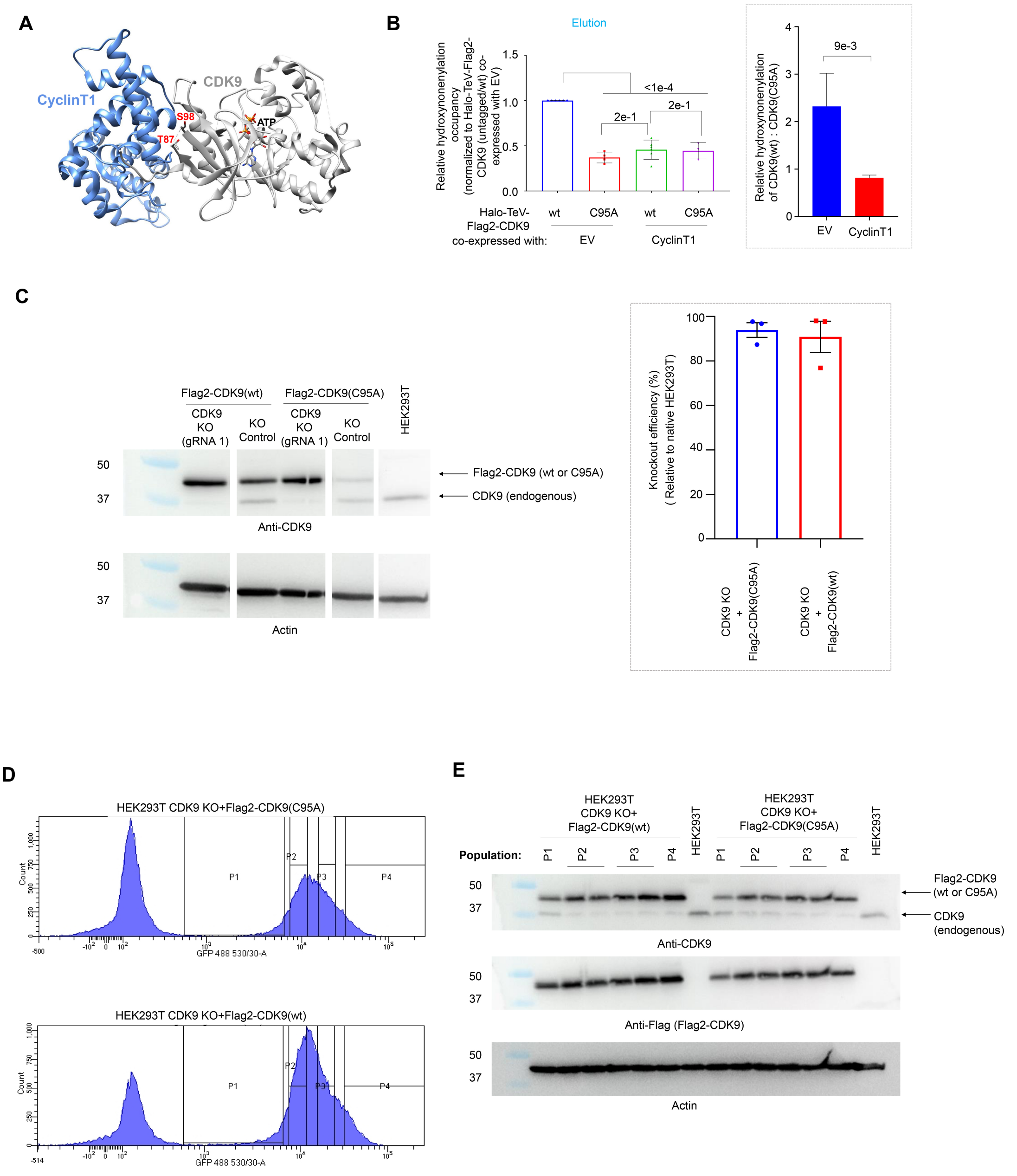

Supplementary Figure 20. CDK9 senses hydroxynonenal principally via C95.

(**A**) Crystal structure of ATP-bound CDK9 complexed with CyclinT1 (PDB:3BLQ). C95 lies on a flexible loop not visible in the structure; but based on the position of nearest visible residues S98 and T87 (indicated in the figure), C95 is likely close to CyclinT1-binding interface.

(**B**) HEK293T cells transiently transfected with the indicated constructs were subjected to T-REX conditions. Quantitation (n = 6 for the first and the third bar, n = 4 for the second bar, n = 3 for the last bar; two-tailed *t*-test was applied). ***Inset*** *on the right:* shows fold-reduction in hydroxynonenylation from CDK9(wt) to CDK9(C95A) in control vs. CyclinT1-overexpressing cells. See **Fig.3A** for representative blot.

(C) Assessment of endogenous CDK9 knockout (KO) prior to GFP-based cell sorting was performed by western blot analysis. Inset: CDK9 KO efficiencies are shown as percentages of the expression in native HEK293T. (n = 3 biological replicates for each condition; the error bars indicate s.e.m.). [Note:

Supplemental full-view gels/blots file shows all conditions tested for the CRISPR-Cas9 mediated KO including three different gRNA sequences. gRNA1 was selected for all subsequent experiments].

(D) Histogram shows the sorted populations by fluorescence-activated cell sorting (FACS) based on GFP expression, which is correlated with Flag2-CDK9(wt) or (C95A) expression. P1: Low; P2: Medium-Low; P3: Medium-High; P4: High GFP-expression levels.

(E) Expression levels of Flag2-CDK9(wt) and Flag2-CDK9(C95A) from sorted populations (P1-P4) were assessed by western blot. P1 population was selected from each cell line as it showed similar expression level as in native HEK293T.

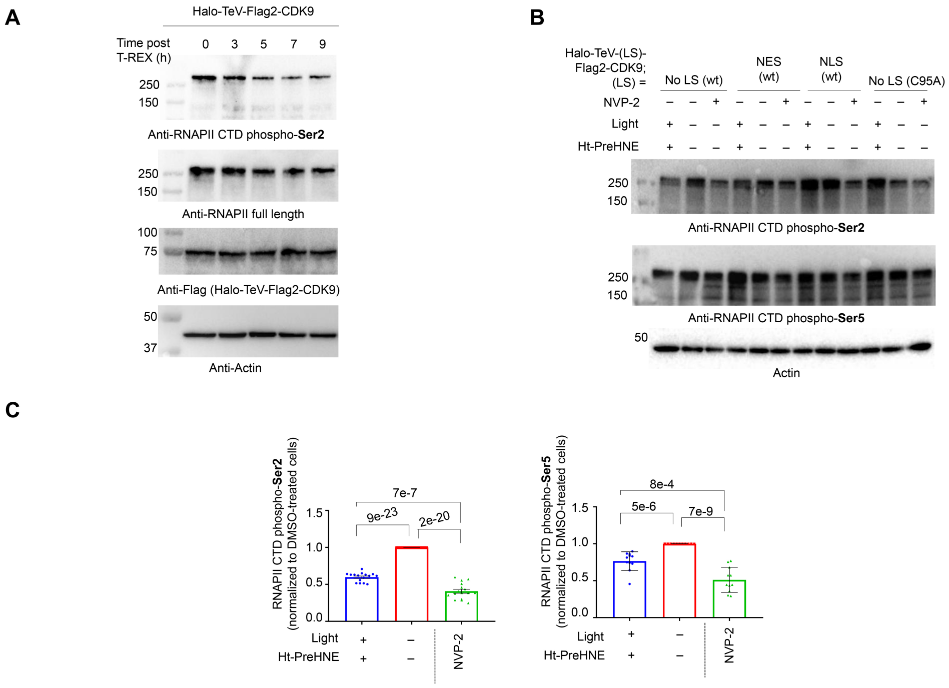

Supplementary Figure 21. Cytoplasm-specific substoichiometric CDK9(C95)-hydroxynonenylation downregulates RNAPII CTD. (NVP-2 treatment where relevant: 200 nM, 2 h).

(**A**) Substoichiometric hydroxynonenylation of CDK9 suppresses RNAPII CTD phospho-*Ser*2. HEK293T cells expressing Halo-TeV-Flag2-CDK9 were subjected to T-REX and harvested at the specific time points post CDK9-specific hydroxynonenylation. Whole-cell lysates were analyzed by western blot using the indicated antibodies. See **Fig.3C** for quantitation.

(**B**) HEK293T cells were transfected with the indicated constructs and were subjected to the indicated conditions. Whole-cell lysates were analyzed by western blot using the indicated antibodies. See **Fig.4A,C** for quantification.

(**C**) Quantification of RNAPII CTD phospho-*Ser2* (left) and phospho-*Ser5* (right) levels comparing the results from T-REX vs. NVP-2-treatment in HEK293T cells expressing Halo-TeV-Flag2-CDK9. Data summarized from all western blot-based experiments in this study. (Biological replicates, n = 16 for RNAPII CTD phospho-*Ser2* data, n = 11 for phospho-*Ser5* data; the error bars indicate s.d.; two-tailed *t*-test was applied).

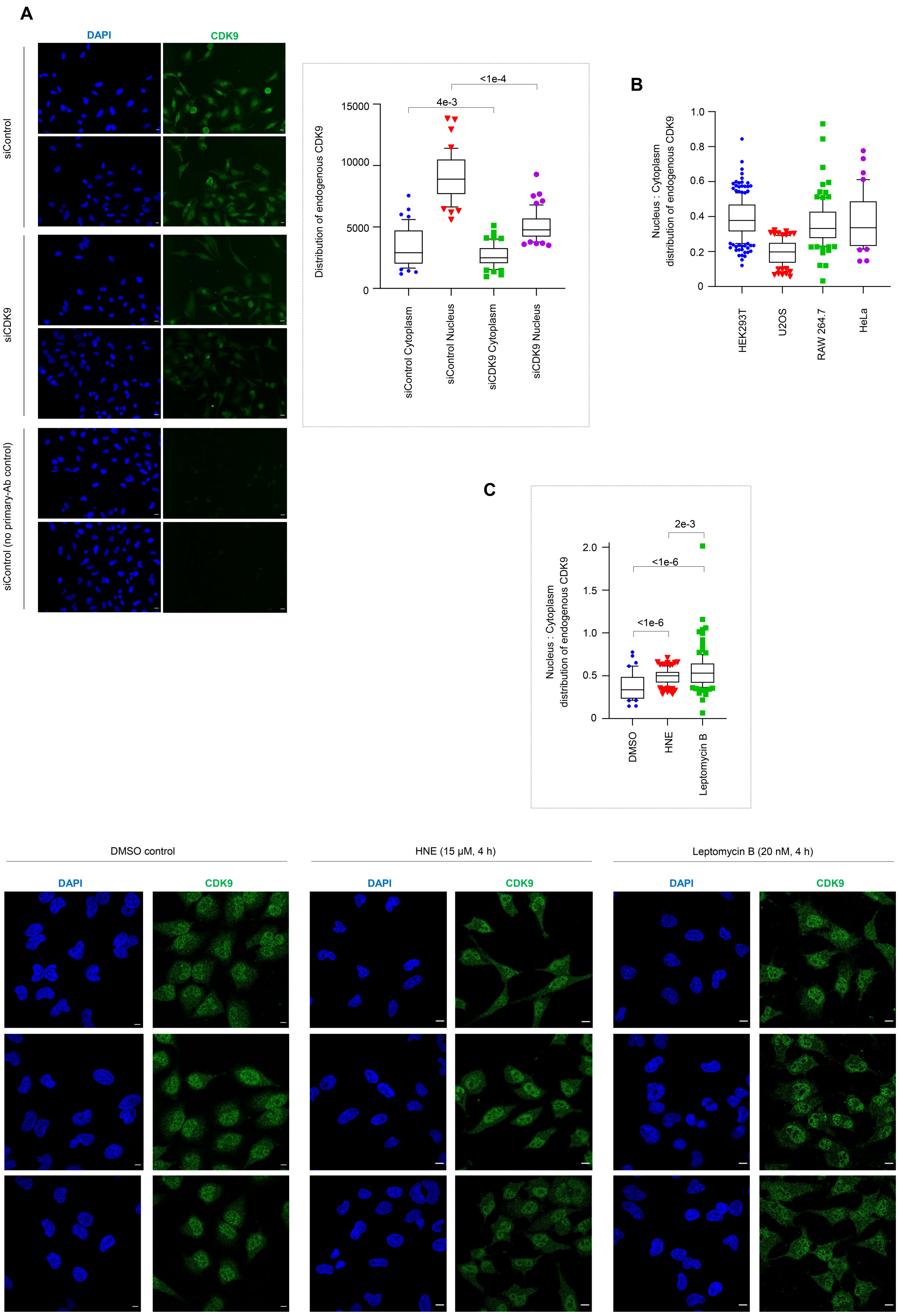

Supplementary Figure 22. Hydroxynonenal treatment enhanced nuclear proportion of endogenous CDK9.

(**A**) Validation of CDK9 antibody (D7 clone, Supplementary Table S5) by immunofluorescence (IF) imaging, following transfection of cultured HeLa cells with either siCDK9 or siControl (Santa Cruz, Supplementary Table S3). Representative IF images post 30-h siRNA-transfection. Scale bars, 15 μm.

Inset: quantification. Mean ± s.e.m.; ($n > 45$ for each bar). For box plots, center lines indicate medians, box limits are the first and third quartiles and whisker ends represent 10–90% confidence intervals. Data not included between the whiskers are plotted dots.

(**B**) Quantification of nucleus:cytoplasm distribution of endogenous CDK9, by IF-imaging in indicated cell lines using anti-CDK9 antibody validated in **A**. Mean ± s.e.m. are presented; ($n > 45$ for each bar). For box plots, center lines indicate median, box limits are the first and third quartiles and whisker ends represent 10–90% confidence intervals. Data not included between the whiskers are plotted as dots.

(**C**) Representative IF images of endogenous CDK9 expression in HeLa cells treated with either HNE (15 μM), Leptomycin B (20 nM), or DMSO, for 4 h. Scale bars, 10 μm. **Inset:** quantification. Mean ± s.e.m.; $n > 108$ for each bar. For box plots, center lines indicate median, box limits are the first and third quartiles and whisker ends represent 10–90% confidence intervals. Data not included between the whiskers are plotted as dots).

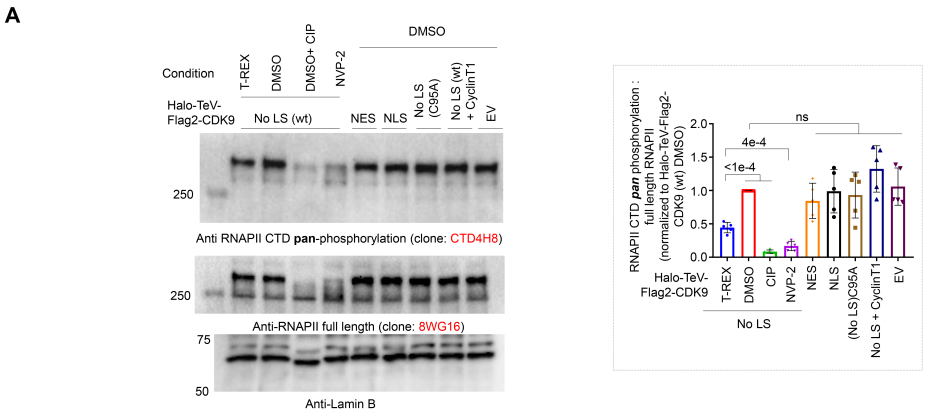

Supplementary Figure 23. Further validations of CDK9-hydroxynonenylation suppressing RNAPII *pan*-phosphorylation, using two indicated independent antibodies.

HEK293T cells were transfected with the indicated constructs and were subjected to indicated conditions. Cells were lysed and normalized for protein content using Bradford assay. For the alkaline calf intestinal phosphatase (CIP) treatment group, 100 units of CIP was added to the normalized cell lysates for 30 min at 37 °C. All samples were resolved on SDS-PAGE gel and analyzed by western blot using the antibodies indicated. The antibody clone CTD4H8 probes RNAPII CTD *pan*-phosphorylation level (top blot); whereas antibody clone 8WG16 probes RNAPII (middle blot). ***Note***: the use of CIP (a non-specific phosphatase) demonstrates that CTD4H8 recognizes specifically the phosphorylated state. Lamin B is used as loading control (bottom blot). ***Inset** below shows* quantification [normalized to DMSO-treated Halo-TeV-Flag2-CDK9 (No LS (wt))]. (n = 4 biological replicates, the error bars indicate s.d.; two-tailed *t*-test was applied).

A

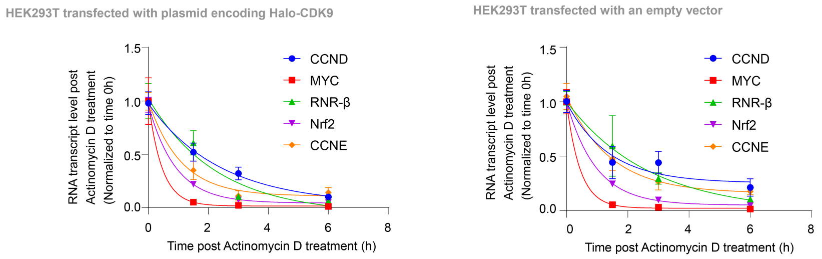

Entry	Gene	RNA transcript level decay t1/2 (h) in cells transfected with plasmids encoding: Halo-CDK9	empty vector
1	CCND	1.7±0.13	0.97±0.2
2	MYC	0.3±0.16	0.3±0.1
3	RNR-β	1.6±0.2	1.6±0.25
4	Nrf2	0.6±0.07	0.6±0.1
5	CCNE	0.7±0.13	0.7±0.16

B

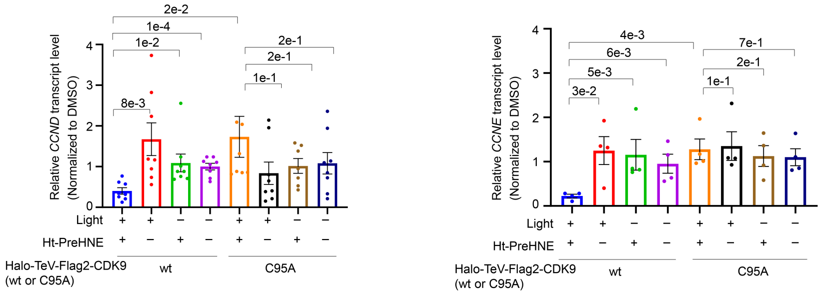

Supplementary Figure 24. $t_{1/2}$ of RNA transcripts decay is similar between the cells transfected with a plasmid encoding HaloCDK9 and empty vector; T-REX-enabled CDK9(C95)-specific hydroxynonenylation can decrease the abundance of short-lived transcripts.

(A) HEK293T cells transfected with either a plasmid encoding Halo-TeV-Flag2-CDK9 (left panel) or an empty vector (right panel) were treated with Actinomycin D (6.5 µg/ml) for the time period indicated. RNAs were extracted and reverse-transcribed, priming off the poly A tail, into the corresponding cDNAs. The expression level of each gene was then analyzed using qPCR (**Supplementary Table S11**). The levels of each transcript at each time point were normalized against GAPDH (housekeeping gene) in each instance, with levels of each transcript also being normalized to its own 0 h time point. ***Inset:*** the data points were fit to one phase decay equation (Y = (1 - Plateau)*exp(-*k**X) + Plateau, where Y is the RNA transcript levels, *k* is the rate constant, and X is the time (h) using Prism) to calculate $t_{1/2}$.

(B) HEK293T cells expressing either Halo-TeV-Flag2-CDK9 (wt) or Halo-TeV-Flag2-CDK9 (C95A) were subjected to T-REX or T-REX controls, and cells were harvested 5 h post T-REX execution. RNAs were extracted and reverse-transcribed, priming off the poly-A tail, into the corresponding cDNAs (**Supplementary Table S11**). The levels of transcripts encoding CCND and CCNE were then analyzed using qPCR. The levels of each transcript at each time point were normalized against *GAPDH* (housekeeping gene) in each instance, with levels of each transcript also being normalized to its own 0 h time point. Also see **Fig.4E**. (n = 8 biological replicates for *CCND*; n = 4 biological replicates for *CCNE*, the error bars indicate s.e.m.; two-tailed *t*-test was applied).

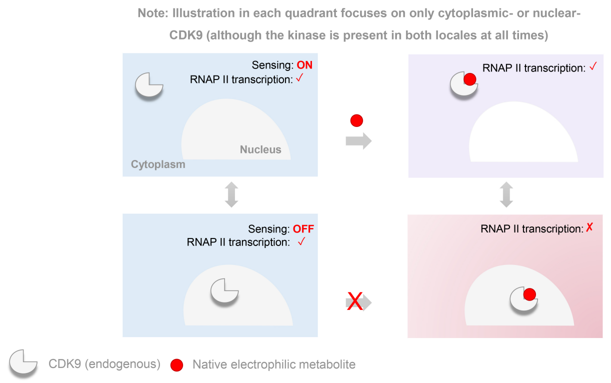

Supplementary Figure 25. The working model for cytoplasmic-specific native electrophilic-metabolite-sensing by CDK9.

The model for cytoplasmic-specific native electrophilic-metabolite sensing by CDK9. (***Note**: endogenous kinase is present in both cytoplasm and nucleus at any given time, but each of the four scenarios focuses only on either cytoplasmic- or nuclear-CDK9*). *Bottom left:* Nuclear CDK9 is kinase active, but cannot sense the electrophile. *Top left:* Cytoplasmic CDK9 cannot modulate RNAPII CTD or phospho-*Ser*2 levels; *Top right:* only cytoplasm-localized CDK9 can sense the electrophile. *Bottom right:* Once in the nucleus, substiochiometrically hydroxynonenylated CDK9 elicits a dominant-negative suppression of RNAPII CTD phospho-*Ser*2.

Full-view data from western blot and FACS analyses corresponding to indicated figures:

Figure 1

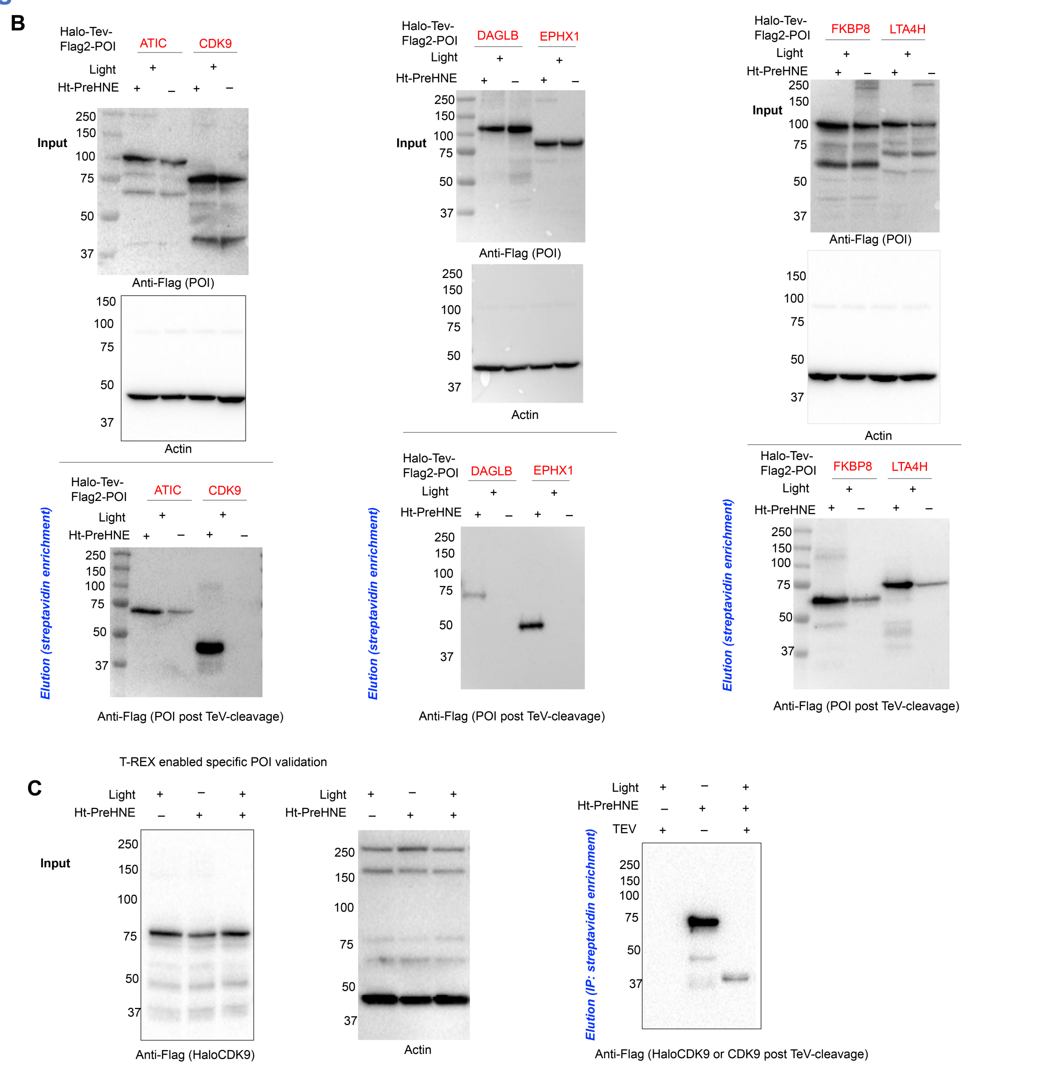

Figure 2

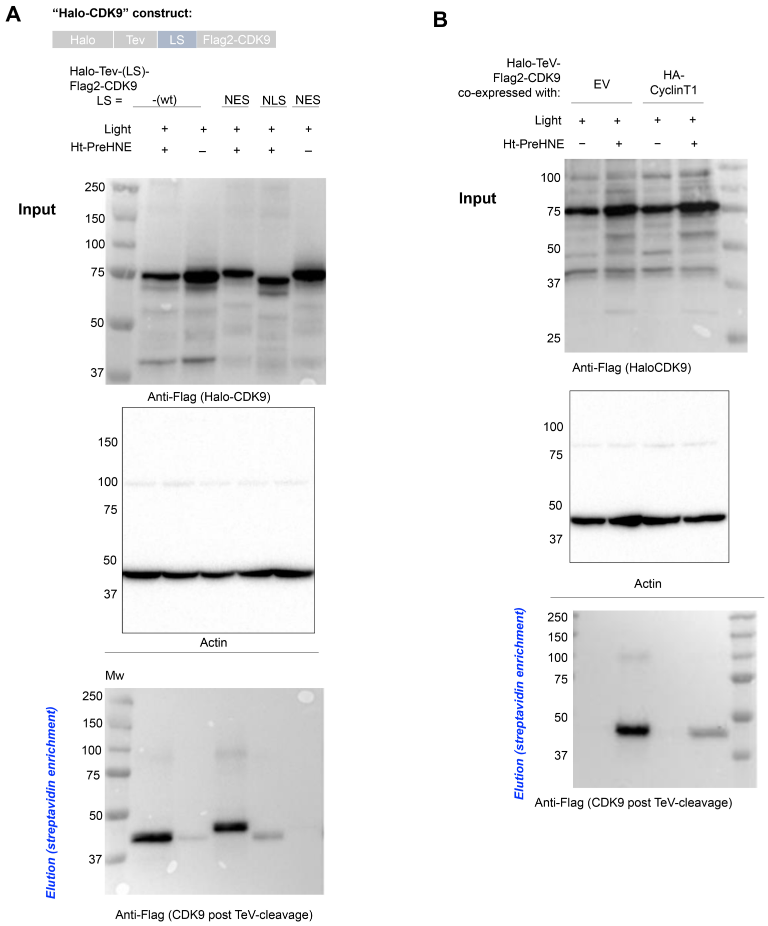
A
"Halo-CDK9" construct:
Halo
Tev
LS
Flag2-CDK9
Halo-Tev-(LS)-Flag2-CDK9
LS =
-(wt)
NES
NLS
NES
Light
Ht-PreHNE
Input
250
150
100
75
50
37
Anti-Flag (Halo-CDK9)
Actin
Mw
Elution (streptavidin enrichment)
Anti-Flag (CDK9 post TeV-cleavage)
B
Halo-TeV-Flag2-CDK9 co-expressed with:
EV
HA-CyclinT1
25
Anti-Flag (HaloCDK9)

Figure 3

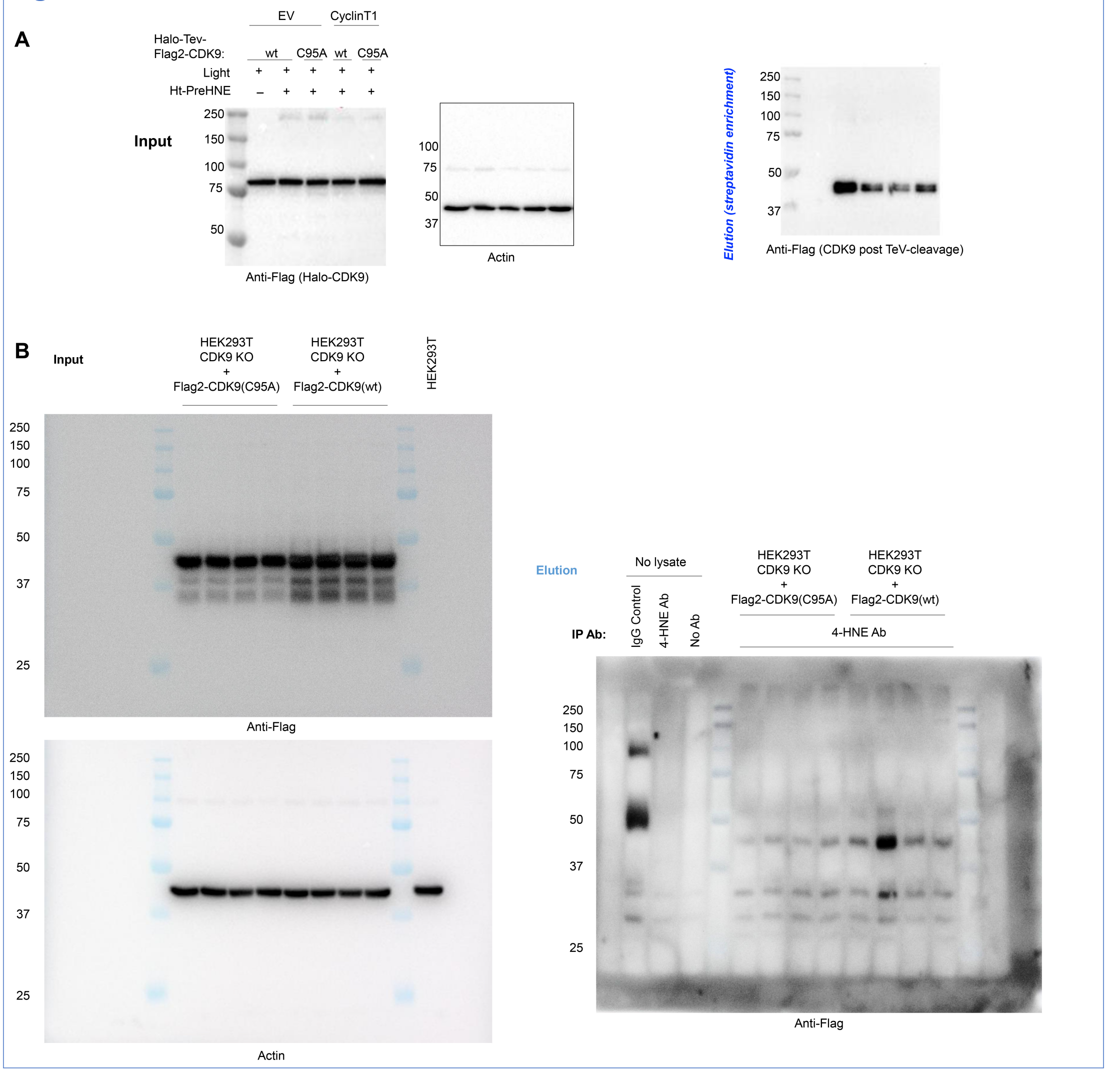
A
EV
CyclinT1
Halo-Tev-
Flag2-CDK9:
wt
C95A
wt
C95A
Light
Ht-PreHNE
Input
250
150
100
75
50
Anti-Flag (Halo-CDK9)
100
75
50
37
Actin
Elution (streptavidin enrichment)
250
150
100
75
50
37
Anti-Flag (CDK9 post TeV-cleavage)
B
Input
HEK293T
CDK9 KO
+
Flag2-CDK9(C95A)
HEK293T
CDK9 KO
+
Flag2-CDK9(wt)
HEK293T
250
150
100
75
50
37
25
Anti-Flag
250
150
100
75
50
37
25
Actin
Elution
No lysate
HEK293T
CDK9 KO
+
Flag2-CDK9(C95A)
HEK293T
CDK9 KO
+
Flag2-CDK9(wt)
IP Ab:
IgG Control
4-HNE Ab
No Ab
4-HNE Ab
250
150
100
75
50
37
25
Anti-Flag

Supplementary Figure 5 (part 1 of 3)

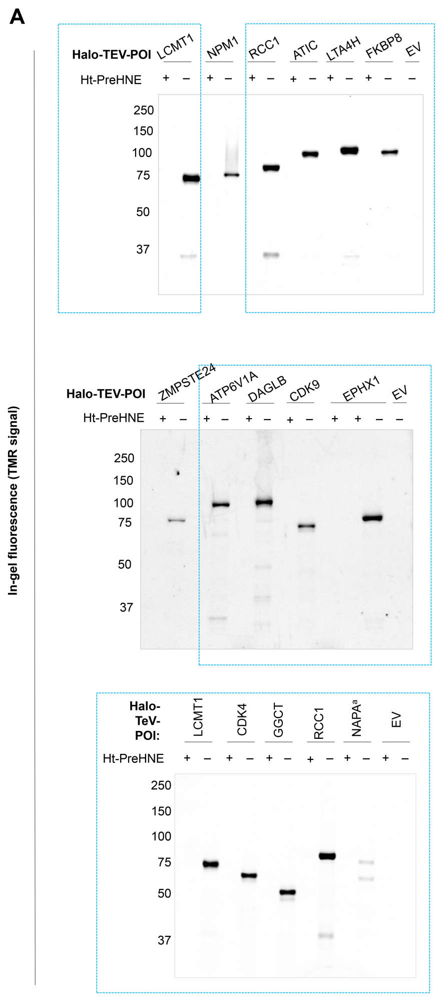

Data from S5A on TMR readout.

Rectangular dotted boxes correspond to data discussed in the main text. See Fig.S3-4 for reasons underlying selection of these genes for further investigations.

Note: The gel-loading for the in-gel fluorescence and the corresponding western blot analyses are arranged so that that relative expression comparison can be made despite these gels/blots being split over multiple gels/blots. For example, the LCMT1 signal is present in both the top and the bottom gels. The ATP6V1A signal is present in both the middle gel and the gel on Supplementary Figure 5 (see part 3 of 3 below).

Supplementary Figure 5 (part 2 of 3)

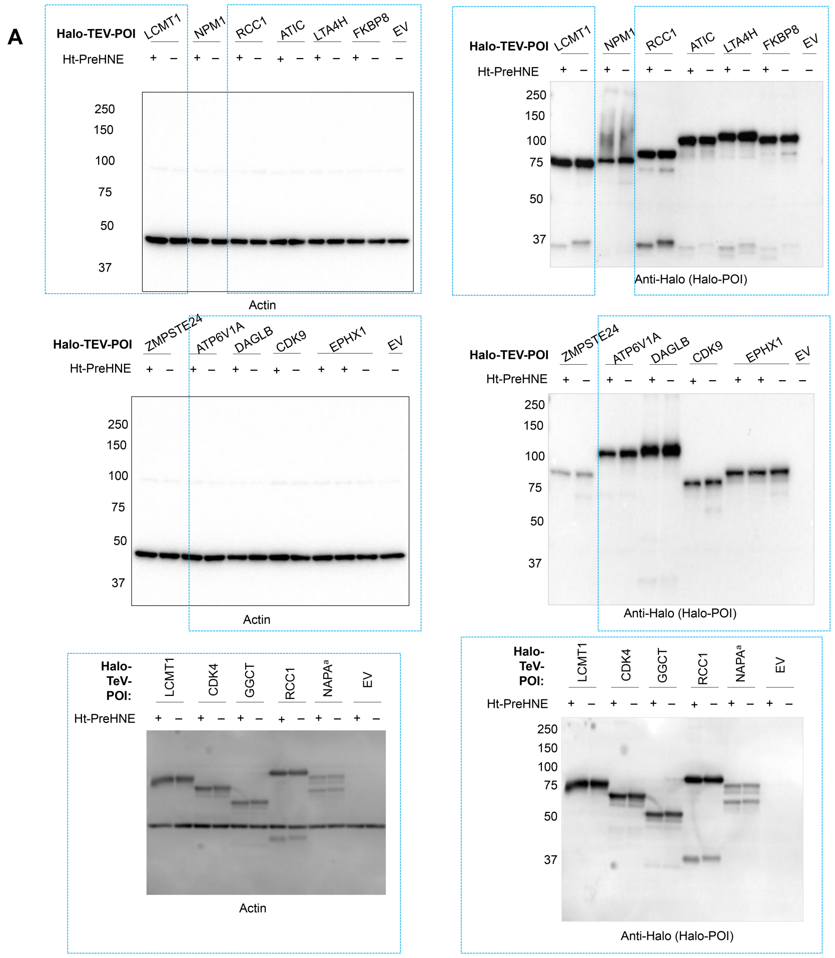

Data from S5A on western blot analyses.

Rectangular dotted boxes correspond to data discussed in the main text. See Fig.S3-4 for reasons underlying selection of these genes for further investigations. (See Note in Part 1 of 3 above, regarding how comparison on multiple gels/blots was ensured to be valid in these experiments.)

Supplementary Figure 5 (part 3 of 3)

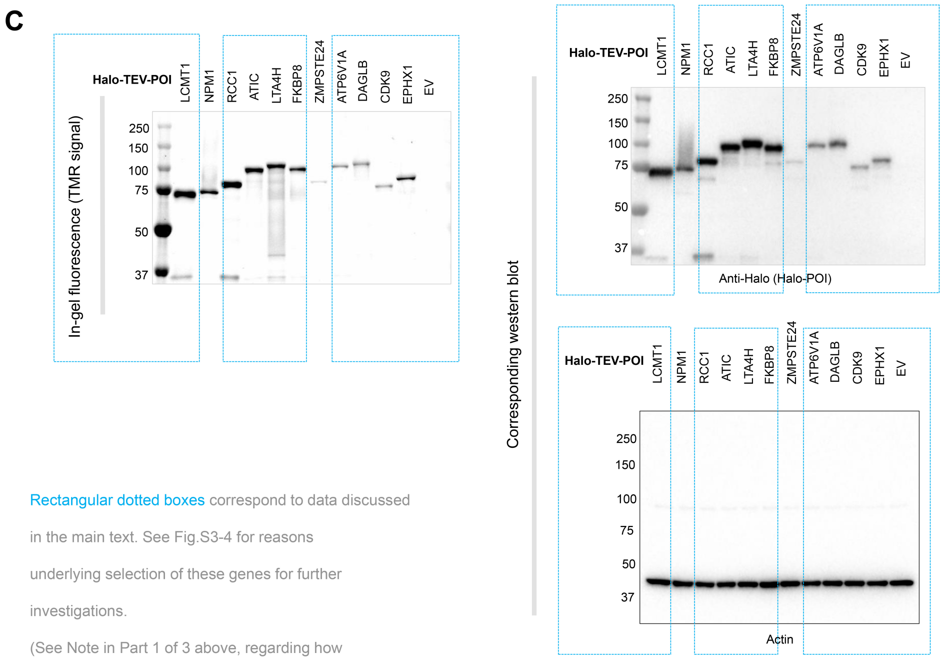

Rectangular dotted boxes correspond to data discussed in the main text. See Fig.S3-4 for reasons underlying selection of these genes for further investigations.

(See Note in Part 1 of 3 above, regarding how comparison on multiple gels/blots was ensured to be valid in these experiments.)

Supplementary Figure 7

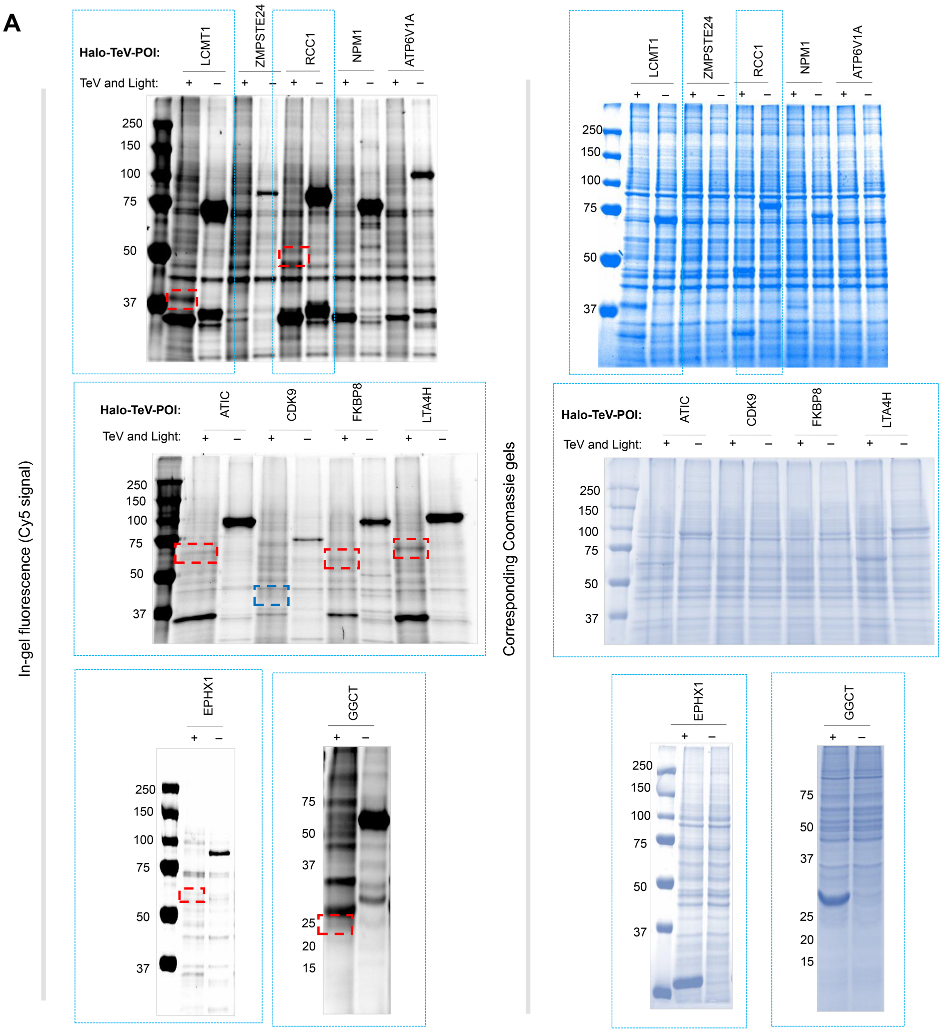

Rectangular dotted boxes correspond to data discussed in the main text. See Fig.S3-4 for reasons underlying selection of these genes for further investigations. (See Note in Part 1 of 3, Fig.S5 above regarding how comparison on multiple gels/blots was ensured to be valid in these experiments.)

Supplementary Figure 9

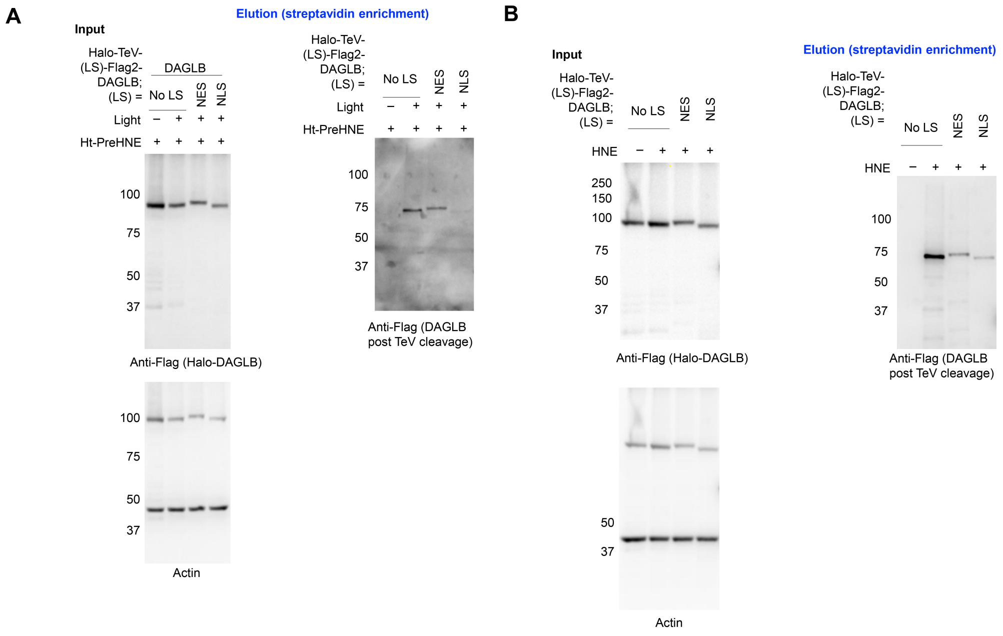

Supplementary Figure 10

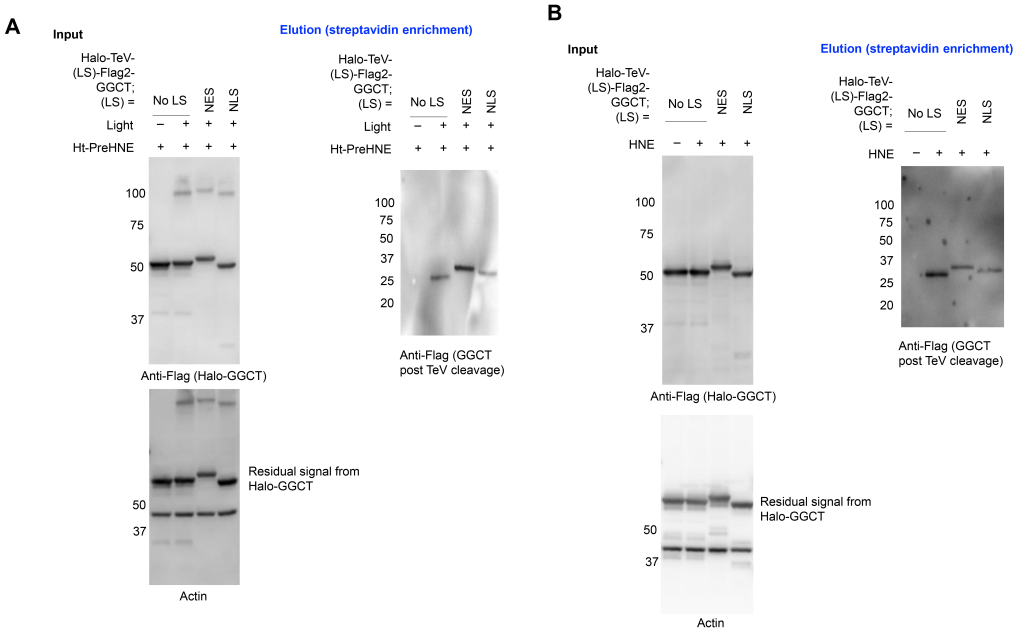

Supplementary Figure 11

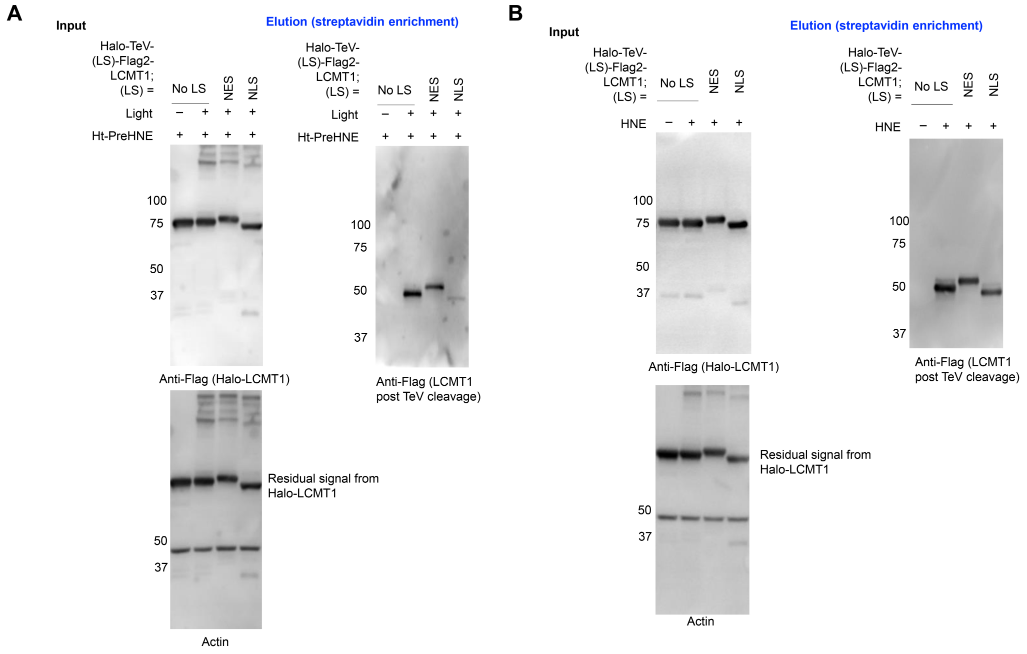

Supplementary Figure 12

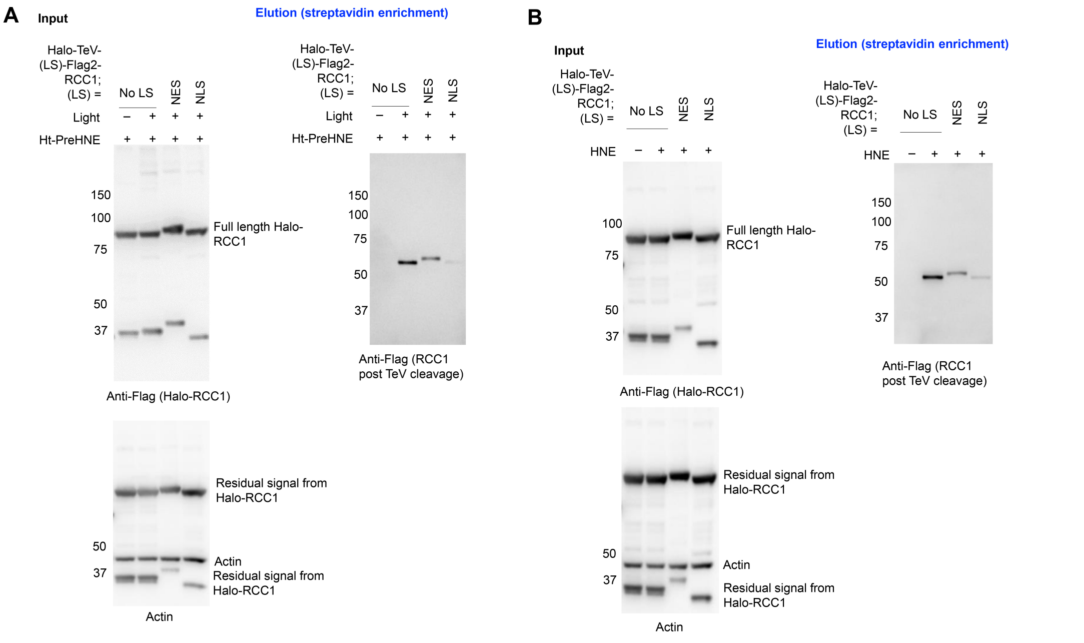

Supplementary Figure 13

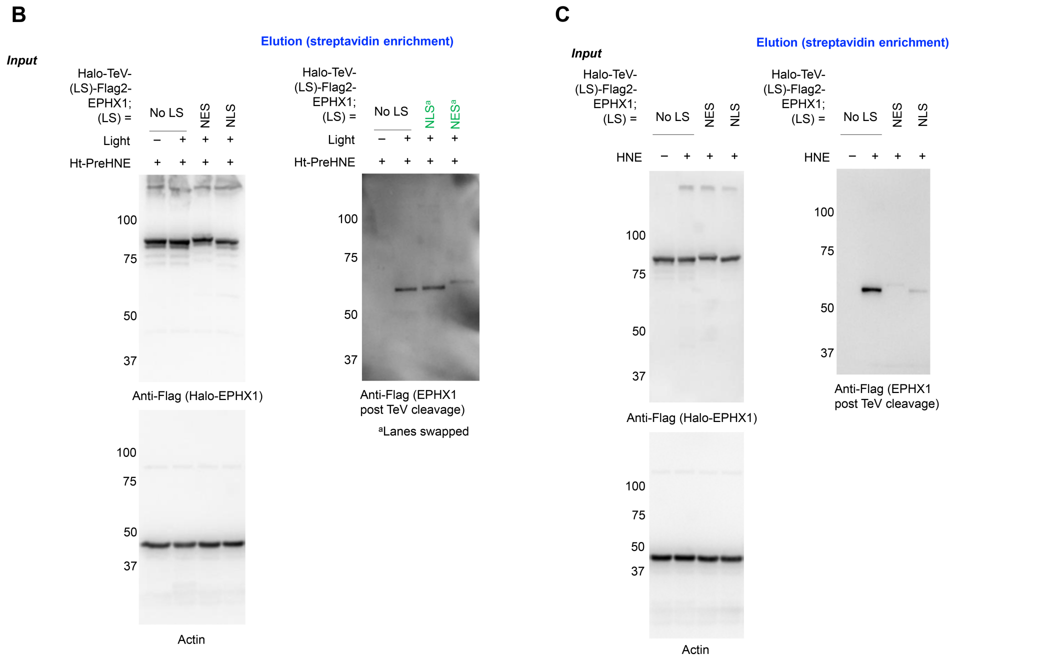

Supplementary Figure 14

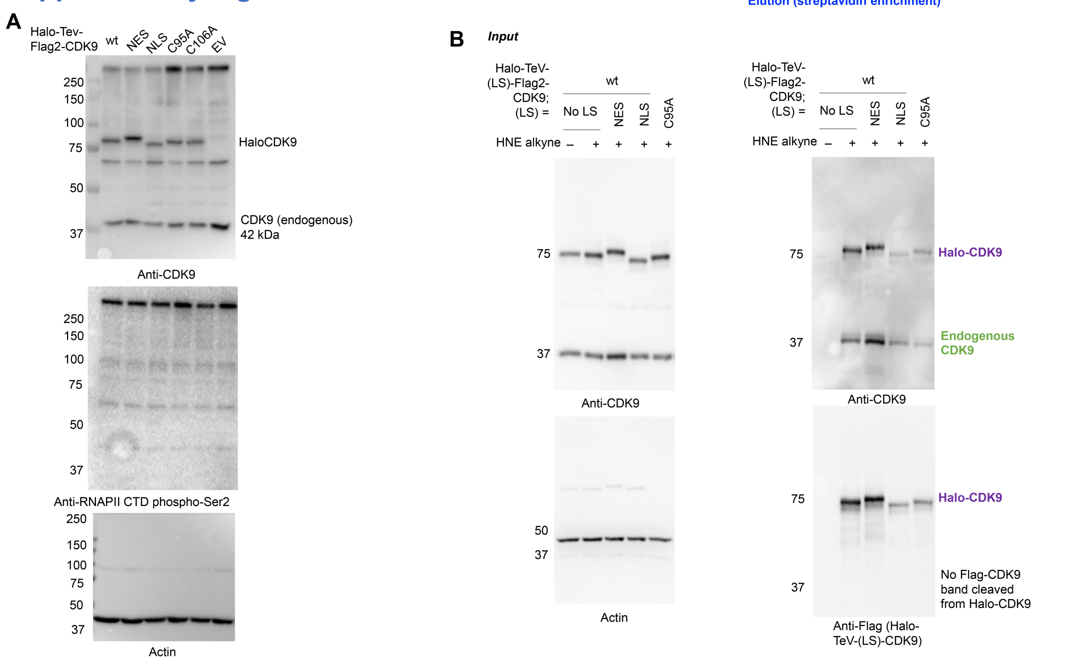

Supplementary Figure 17

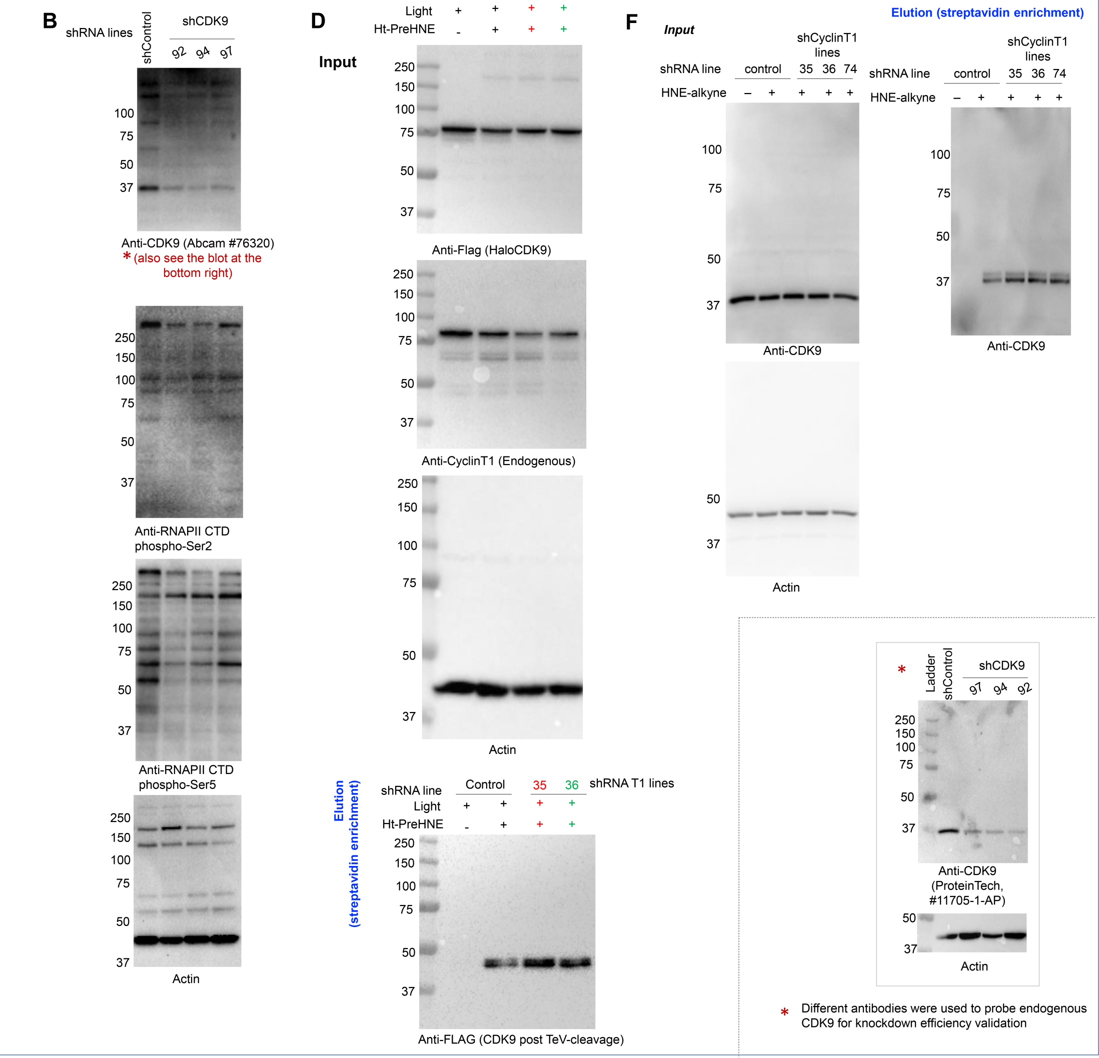

Supplementary Figure 18

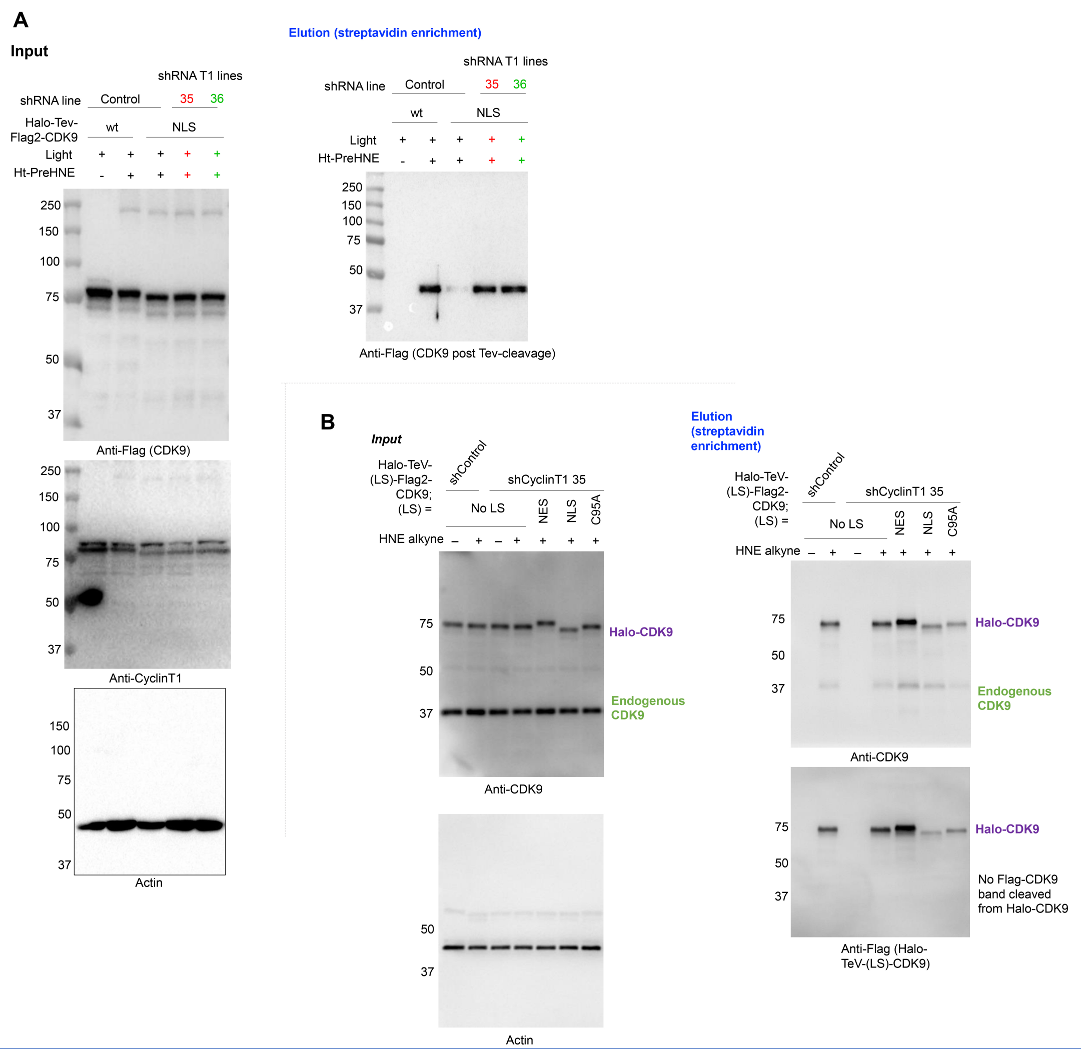

Supplementary Figure 19

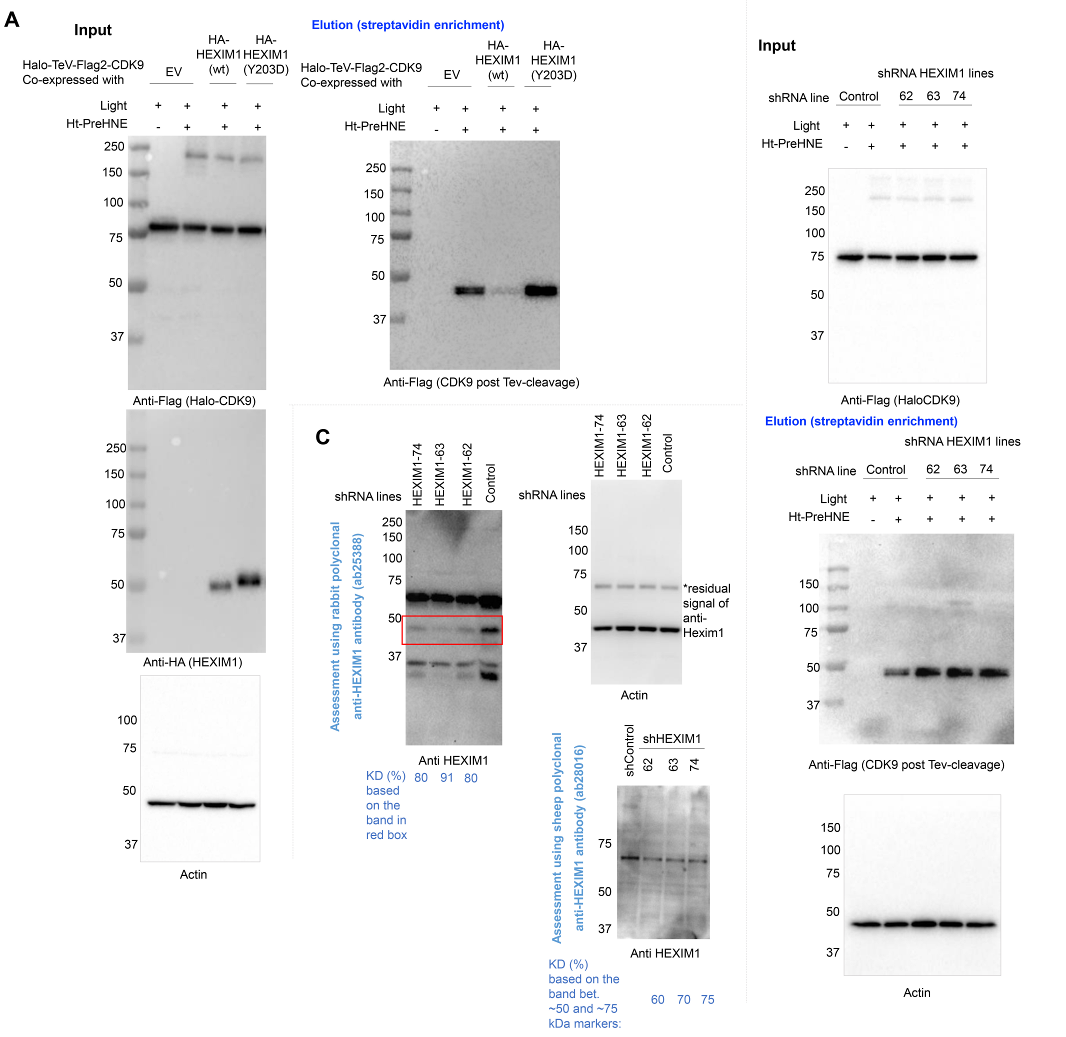

Supplementary Figure 20 (Part 1 of 2)

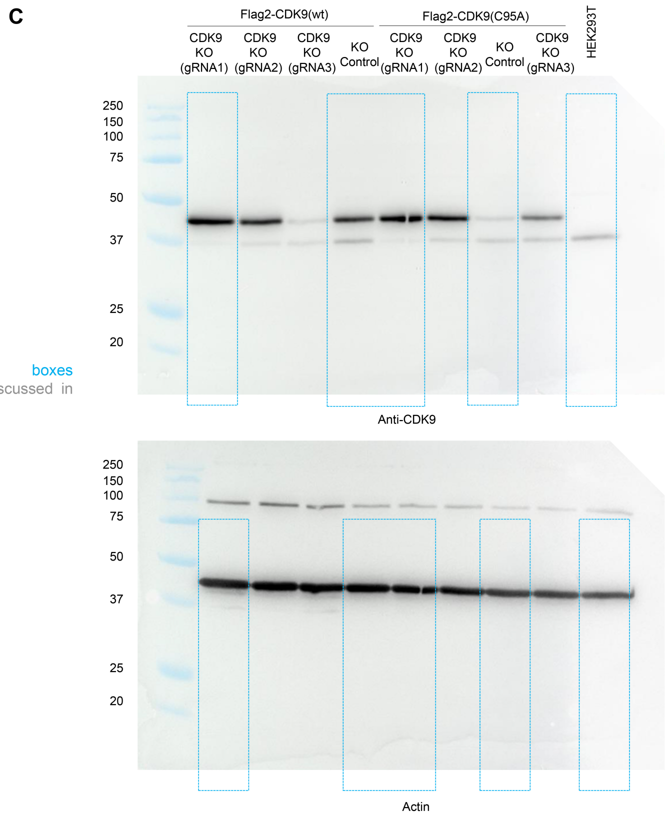

Rectangular dotted boxes correspond to data discussed in the main text.

Note: Three gRNAs were used for knockout line generation using HEK293T. Since gRNA 1 showed the highest KO efficiency, it was used for all subsequent experiments.

Supplementary Figure 20 (Part 2 of 2)

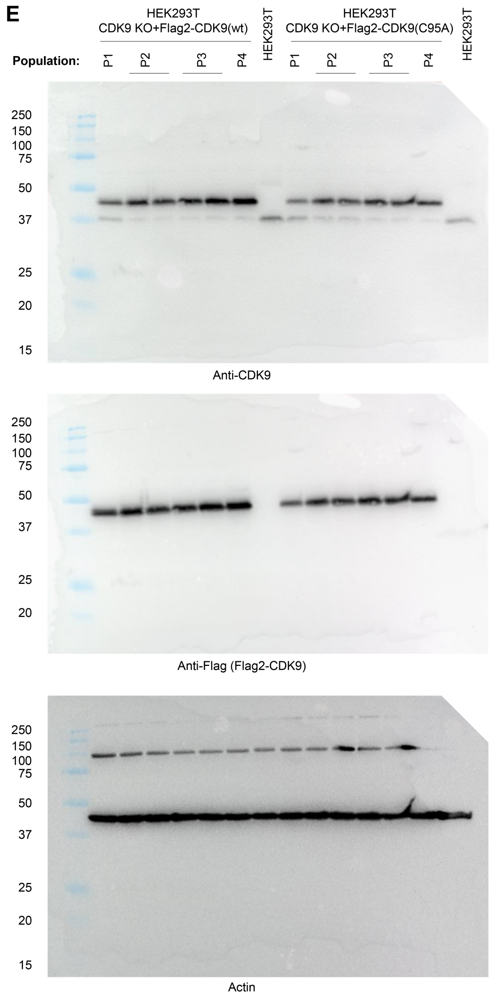

Supplementary Figure 21

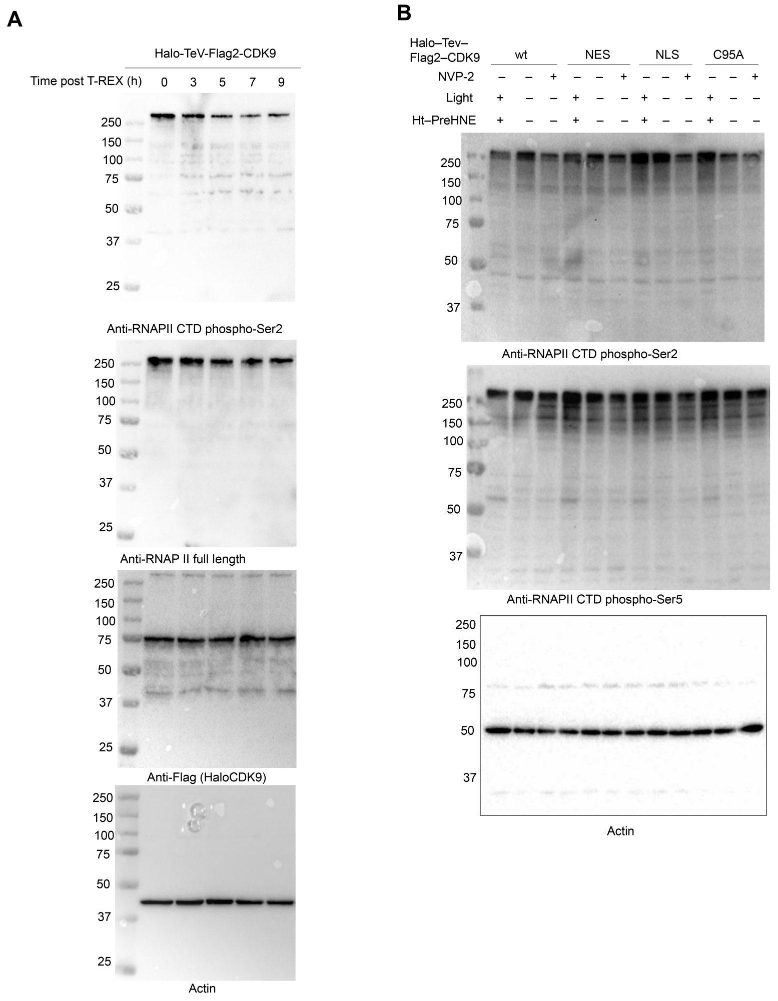

Supplementary Figure 23

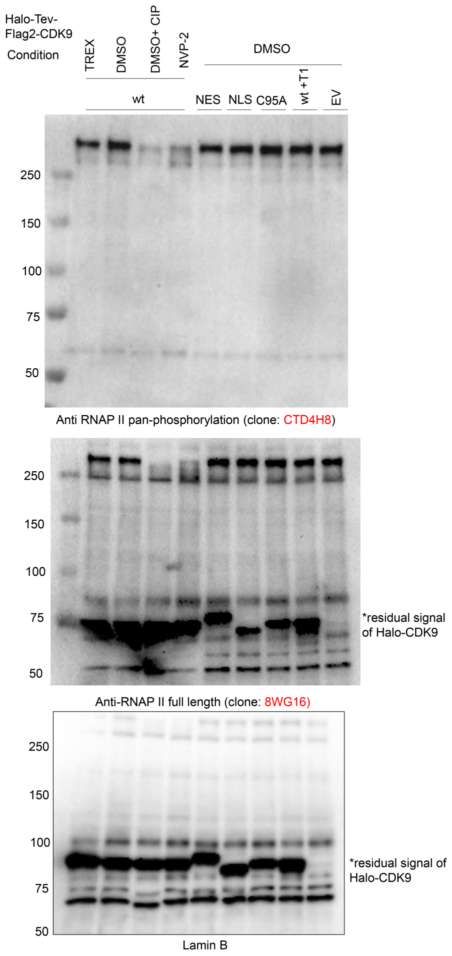

FACS Sorting: data analysis (related to supplemental figure S20C-E)

HEK293T CDK9 KO+Flag2-CDK9(wt)

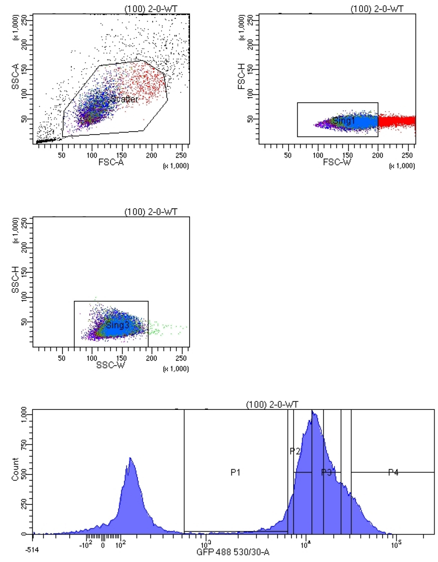

Tube: WT

Population	#Events	%Parent	%Total
All Events	64,937	####	100.0
Scatter	50,000	77.0	77.0
Sing1	43,117	86.2	66.4
Sing3	43,063	99.9	66.3
P1	2,587	6.0	4.0
P2	9,111	21.2	14.0
P3	6,296	14.6	9.7
P4	2,831	6.6	4.4

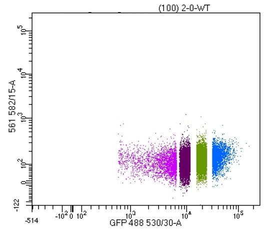

HEK293T CDK9 KO+Flag2-CDK9(C95A)

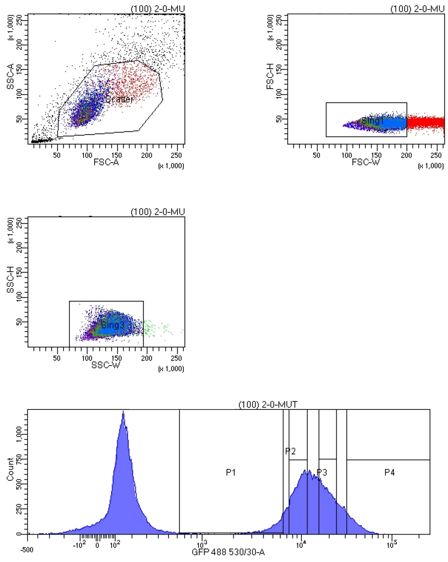

Tube: MUT

Population	#Events	%Parent	%Total
All Events	68,323	####	100.0
Scatter	50,000	73.2	73.2
Sing1	41,623	83.2	60.9
Sing3	41,552	99.8	60.8
P1	2,135	5.1	3.1
P2	6,231	15.0	9.1
P3	4,883	11.8	7.1
P4	1,645	4.0	2.4

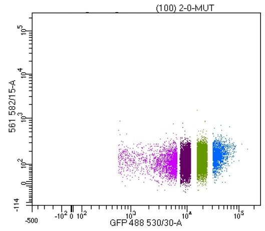

Supplemental Materals and Methods: see also General Meterials and Methods section in the Main Manuscript.

In-gel trypsin digestion of SDS gel bands:

The protein bands from an SDS-PAGE gel that had been sliced into 4-5 slices for each sample were cut into ~1 mm cubes and subjected to in-gel digestion followed by extraction of the tryptic peptide as reported previously[8].The excised gel pieces were washed consecutively in 400-800 µL distilled water, 100 mM ammonium bicarbonate (Ambic)/acetonitrile (1:1) and 100% acetonitrile. The gel pieces were reduced with 100-300 µL of 10 mM DTT in 100 mM Ambic for 1 hr at 56 °C, alkylated with 100-300 µL of 55 mM Iodoacetamide in 100 mM Ambic at room temperature in the dark for 60 mins. Repeated wash steps as described above, the gel slices were dried and rehydrated with 100-200 µL trypsin in 50 mM Ambic, 10% ACN (5 ng/µL) at 37 °C for 16 hrs. The digested peptides were extracted twice with 200-300 µl of 50% acetonitrile, 5% FA and once with 200-300 µl of 90% acetonitrile, 5% FA. Extracts from each sample were combined, filtered with 0.22 µm spin filter (Costar Spin-X from Corning Incorp) and lyophilized. *Note: wash/reduce/alkylation/digestion volume changes depending on gel band size*

Nano LC/MS/MS Analysis on Orbitrap Fusion:

The SILAC tryptic digests were reconstituted in 50 µL of 0.5% FA estimated at 0.1 µg/µL for nanoLC-ESI-MS/MS analysis, which was carried out using an Orbitrap FusionTM TribridTM (Thermo-Fisher Scientific, San Jose, CA) mass spectrometer equipped with a nanospray Flex Ion Source, and coupled with a Dionex UltiMate3000RSLCnano system[8,9] (Thermo, Sunnyvale, CA). The tryptic peptide samples (10 µL) were injected onto a PepMap C-18 RP nano trap column (5 µm, 100 µm i.d x 20 mm, Dionex) with nanoViper Fittings at 20 µL/min flow rate for on-line desalting. The peptides were separated on a PepMap C-18 RP nano column (2 µm, 75 µm x 25 cm) at 35 °C, in a 120 min gradient of 5% to 38% acetonitrile (ACN) in 0.1% formic acid at 300 nL/min and followed by a 8 min ramping to 90% ACN-0.1% FA and a 9 min hold at 90% ACN-0.1% FA. The column was re-equilibrated with 0.1% FA for 25 min prior to the next run. The Orbitrap Fusion is operated in positive ion mode with spray voltage set at 1.6 kV and source temperature at 275°C. External calibration for FT, IT and quadrupole mass analyzers were performed. In data-dependent acquisition (DDA) analysis, the instrument was operated using FT mass analyzer in MS scan to select precursor ions followed by 3 second "Top Speed" data-dependent CID ion trap MS/MS scans at 1.6 m/z quadrupole isolation for precursor peptides with multiple charged ions above a threshold ion count of 10,000 and normalized collision energy of 30%. MS survey scans at a resolving power of 120,000 (fwhm at m/z 200), for the mass range of m/z 375-1575. Dynamic exclusion parameters were set at 50 s

of exclusion duration with ±10 ppm exclusion mass width. All data were acquired under Xcalibur 3.0 operation software (Thermo-Fisher Scientific).

Data analysis:

All MS and MS/MS raw spectra were processed and searched using Sequest HT software within the Proteome Discoverer 1.4.1.14 (PD 1.4, Thermo Scientific). The *Homo_sapiens*_Uniprot (taxonomy 9606) database (120,672 entries) was used for database searches. The database search was performed under a search workflow with the "Precursor Ions Quantifier" node for SILAC 2plex (Arg10, Lys8) quantitation. The default setting for protein identification in Sequest node were: two mis-cleavages for full trypsin with fixed carbamidomethyl modification of cysteine, variable modifications of 10.008 Da on Arginine and 8.014 Da on lysine, N-terminal acetylation, methionine oxidation and deamidation of asparagine and glutamine residues. The peptide mass tolerance and fragment mass tolerance values were 10 ppm and 0.6 Da, respectively. Only high confidence peptides defined by Sequest HT with a 1% FDR by Percolator were considered for the peptide identification. The mass precision for expected standard deviation of the detected mass used to create extracted ion chromatograms was set to 5 ppm. The SILAC 2-plex quantification method within PD 1.4 was used with unique peptides only to calculate the heavy/light ratios of all identified proteins without normalization. The final protein group list was further filtered with two peptides per protein in which only #1-rank peptides within top-scored proteins were used.

T-REX in cultured cells[1,3]:

HEK293T cells were maintained as described previously[1,3] and mentioned above. For in-gel fluorescence analysis and western blot, ~ 0.5–0.6 X10^6 HEK293T cells were seeded in a 12-well cell culture plate. 24 h later, cells were transfected using TransIT-2020 transfection reagent per the manufacturer's recommendation. Subsequent steps were performed under red light. 24–36 h post transfection, monolayer of cells was treated with 20 µM Ht-PreHNE in serum-free media and incubated for 2 h. Cells were gently rinsed with serum-free media three times every 30 min over the next 1.5 h. (including the time for rinsing cycles that wash away the excess photocaged probe unbound to Halo, the overall time for this step is ~ 2 h). Meanwhile, UV lamps were turned on 10 min prior to UV irradiation. For samples designated for light exposure, lids were removed from the dishes and cells were irradiated for 5–8 min. The cells were harvested, washed two times with ice-cold DPBS and frozen in liquid nitrogen.

In-gel fluorescence assay:

All steps in this section were performed under red light, unless otherwise stated. Cells from one well of a 12 well plate were lysed in 30 μL buffer containing 50 mM HEPES (pH 7.6), 150 mM NaCl, 1% Nonidet P–40, 1X Roche cOmplete, mini, EDTA-free protease inhibitor cocktail, and 0.3 mM TCEP by rapid freeze-thaw (x3). Cells debris was removed by centrifugation at 18,000 x g for 8-10 min at 4 °C. Protein concentration of the clarified lysate was determined using Bradford assay. A portion of the lysate protein was made up to 22 μL final volume containing, in final concentrations, 50 mM HEPES (pH 7.6), 150mM NaCl, 1.0 mg/mL lysate protein, 0.3 mM TCEP, and 0.2 mg/mL TEV protease. The sample was incubated at 37 °C for 45 min, and subsequently subjected to Click reaction (that was set up in dim red light, and run in the dark). In a final volume of 27 μL, the click reaction mix consisted of 1.7 mM TCEP, 5% *t*-BuOH, 1% SDS, 1 mM $CuSO_4$, 0.1 mM Cu(TBTA), 10 μM Cy5 azide and the lysate from above. The samples were incubated at 37 °C for 30 min and subsequently quenched with 5 μL 4X Laemmeli dye containing 6% βME. After additional 5-min incubation at 37 °C, 25 μL of the lysate was subjected to SDS-PAGE. After electrophoresis, the gel was rinsed 3X with ddH_2O with 5-min each rinse on a shaker and imaged on a Biorad Chemidoc-MP Imager. Where applicable, the gel was transferred to a PVDF membrane for western blot analysis.

Click biotin-azide and streptavidin pull-down for mammalian lysate:

HEK293T cells (~5–6 $X10^6$) were seeded in a 10-cm tissue culture plate. After reaching 70-80% confluence (~18–24 h), cells were transfected with the designated plasmids and TransIT 2020 reagent per the manufacturer's recommendation for 24–36 h. The cells were treated with 20 μM Ht-PreHNE for 2 h. Rinsing and light shining protocol were as described above in T-REX assays. Cells were harvested, washed twice with chilled 1X DPBS and flash-frozen in liquid nitrogen. Mammalian cell lysis was performed in 200 μL of lysis buffer containing in final concentrations 50 mM HEPES (pH 7.6), 150 mM NaCl, 1% Nonidet P-40 and 1X Roche cOmplete, mini, EDTA-free protease inhibitor cocktail by rapid freeze-thaw (x3). Lysate was clarified by centrifugation at 18,000 x g for 8 min at 4 °C. Total protein concentration was determined using Bradford assay relative to BSA as standard. The lysate was subsequently diluted to 2 mg/mL with a buffer made up of 50 mM HEPES (pH 7.6) and 0.3 mM TCEP before subjected to TeV protease cleavage (0.2 mg/mL final) and Click reaction with biotin-azide for 30 min at 37 °C, respectively. The final concentrations of each component were: 1% SDS, 5% *t*–BuOH, 200 μM biotin-azide, 2 mM TCEP, 0.9 mM $CuSO_4$ and 0.1 mM Cu(TBTA). The lysate proteins were precipitated by adding 4 volumes of EtOH pre-chilled at −20 °C (EtOH final concentration is 80 % v/v). The sample was vortexed briefly and incubated at −80 °C overnight (or for at least 4 h) to facilitate protein precipitation. The precipitate was collected by centrifugation at 21,000 x g for 80-120 min at 4 °C and

washed twice with pre-chilled EtOH (80%), once with pre-chilled acetone. The pellet was air-dried, then dissolved in 20–50 µL 50 mM HEPES (pH 7.6), 4% LDS and 1 mM EDTA and dissolved by vortexing and heating at 50-60 °C until the protein pellet dissolved. LDS was diluted to a final concentration of 0.5% with 50 mM HEPES (pH 7.6) and added to 50 µL of Streptavidin sepharose beads pre-equilibrated with 50 mM HEPES (pH 7.6) and 0.5% LDS. The sample was incubated with beads for 2–3 h at room temperature by end-over-end rotation after which time the supernatant was removed by centrifugation at 500 x g for 3 min. The beads were washed three times with 500 µL of 50 mM HEPES (pH 7.6) with 0.5% LDS with end-over-end rotation at room temperature for 30 min during each wash. The bound protein was eluted by boiling the beads at 98 °C for 10 min with 30 µL of 3 X Laemmeli dye containing 6% βME. The sample was subjected to SDS-PAGE and transferred to a PVDF membrane for western blot analysis.

Anti-Flag pulldown for mammalian cell lysate:

HEK293T cells (~5–6 X10^6) were seeded in a 10 cm tissue culture plate. After the cells reached 70-80% confluence (~18–24 h), the old media was replaced with 8 mL of fresh complete media. Cells were transfected with 7.5 µg of the designated plasmid and TransIT 2020 reagent per the manufacturer's recommendation for 24–36 h. The cells were treated with 20 µM Ht-PreHNE for 2 h. Rinsing and light shining protocol were as described above. Cells were harvested, pooled, washed twice with ice-chilled 1X DPBS and flash-frozen in liquid nitrogen. Cell lysis was performed in 100-200 µL per 1.5×10^6 cells of lysis buffer [containing in final concentrations 50 mM HEPES (pH 7.6), 150 mM NaCl, 1% Nonidet P-40 and 1X Roche cOmplete, mini, EDTA-free protease inhibitor cocktail], followed by rapid freeze-thaw cycles (x3). Lysate was clarified by centrifugation at 18,000 x g for 10 min at 4 °C. Total protein concentration was determined using Bradford assay using BSA as standard. The lysate was subsequently diluted to 2 mg/mL with binding buffer containing in final concentrations 50 mM HEPES (pH 7.6), 150 mM NaCl, 1X Roche cOmplete, mini, EDTA-free protease inhibitor cocktail, and 0.1% Tween-20. This diluted lysate was subjected to either 50–100 µL bed volume of ANTI-Flag® M2 affinity agarose gel (A2220, Sigma) that had been pre-equilibrated with the binding buffer above. The sample was incubated with beads overnight at 4 °C by end-over-end rotation after which time the supernatant was removed post-centrifugation at 500 x g. The beads were washed three times at 4 °C with 500 µL wash buffer containing in final concentrations 50 mM HEPES (pH 7.6), 150 mM NaCl, 1X Roche cOmplete, mini, EDTA-free protease inhibitor cocktail, and 0.1% Tween-20, using end-over-end rotation over 10 min during each wash. The bound protein was eluted by either incubating with 0.15 mg/mL 3 X Flag peptide for 2 h at 4 °C or by boiling the beads at 98 °C for 10 min with 30 µL of 3X Laemmeli dye containing 6% βME. The sample was subjected to SDS-PAGE and transferred to a PVDF membrane for western blot analysis described above.

Immunoprecipitation (IP) of:

(i) **hydroxynonenylated CDK9 from non-hydroxynonenal treated cells using anti-HNE Ab;**

(ii) **endogenous CDK9 from hydroxynonenal non-treated and treated cells using anti-CDK9 Ab, followed by mass-spectrometry (MS) analysis.**

(i) Anti-HNE IP: Cells were grown in 10 cm tissue culture plates until 85-90% confluent and harvested using trypsin. The cell pellets were washed two times with DPBS and one time with 50mM HEPES, 150mM NaCl (pH 7.6) prior to flash-freezing in liquid nitrogen. 100 µL of protein G resin (20397, Thermo Fisher) was pre-equilibrated by three sequential washes using wash buffer (25 mM Tris pH=7.4, 150 mM NaCl, 0.1% Nonidet P-40). 5 µg of the primary antibody was transferred into the resin in a final volume of 300 µL per condition. The resin was incubated with the relevant antibodies overnight at 4°C with end-over-end rotation. The cell pellets were lysed the following day using 100 µL lysis buffer (25 mM Tris pH=7.4, 150 mM NaCl, 1% Nonidet P-40, and 2X cOmplete mini, EDTA-free protease inhibitor cocktail) by three rapid freeze-thaw cycles. Lysates were clarified by centrifugation at 20,000 x g for 10 min at 4 °C. Following measurement of total lysate protein concentration by Bradford, the lysates were diluted to 1.5 mg/mL using the same lysis buffer indicated above. 6 mL of lysate was then precleared using 600 µL of pre-equilibrated and nonconjugated protein G resin per sample for 1 h at 4 °C with end-over-end rotation. The precleared lysates were collected post centrifugation at 1,000 x g for 5 min. The protein G resin incubated with antibodies was washed two times with wash buffer for 5 min per wash at 4 °C followed by centrifugation at 1,000 x g. 1 mL of the precleared lysate was added to the washed protein G resin and incubated at room temperature for 1 h. After each incubation step, the lysate was removed post centrifugation at 1,000 x g, and replaced by fresh lysate a total of four times. For the last incubation step, the lysates were incubated overnight at 4 °C. The next day, the lysate was removed, and the resin was washed three times with wash buffer for 10 min per wash at 4 °C. Proteins were eluted by adding 30 µL of 2X Laemmli buffer (4% SDS, 20% glycerol, 120 mM Tris, 0.02% bromophenol blue, 50 mM TCEP pH=6.8) and heating the samples at 42°C for 8 min. The resin was spun down at 20,000 x g for 10 min at room temperature, and the supernatant was collected for subsequent SDS-PAGE and western blot analyses.

(ii) Anti-CDK9 IP followed by MS analysis: The same procedure described above was followed except for the elution sample. 40uL of 2X modified Laemmli buffer (4% SDS, 20% glycerol, 120 mM Tris, 10 mM TCEP, pH=6.8) was added to the washed resin. The samples were heated at 42°C for 8 min. After the final centrifugation step (20,000 x g for 10 min), the supernatant was collected and 40 mM iodoacetamide

was added. The alkylation step was performed at 37°C for 45 min. Additional TCEP was added to a final concentration of 50 mM and 0.02% bromophenol blue. The samples were then subjected to SDS-PAGE. MS-based proteomics-related experiments were performed by the Proteomics Core Facility at EPFL. The gel pieces containing proteins were excised and washed three times with 50% ethanol in 50 mM ammonium bicarbonate for 20 min and dried by vacuum centrifugation. Proteins were digested overnight at 37°C using sequencing grade ArgC and AspN in 50 mM ammonium bicarbonate. Resulting peptides were extracted two times in 70% ethanol, 5% formic acid for 20 min, and dried by vacuum centrifugation.

Peptides were desalted on C18 StageTips[10] and dried by vacuum centrifugation prior to LC-MS/MS injections. Samples were resuspended in 2% acetonitrile, 0.1% formic acid and nano-flow separations were performed on a Dionex Ultimate 3000 RSLC nano UPLC system (Thermo Fischer Scientific) on-line connected with a Lumos Fusion Orbitrap or an Exploris 480 Orbitrap Mass Spectrometer (Thermo Fischer Scientific). A capillary precolumn (Acclaim Pepmap C18, 3 μm-100Å, 2 cm x 75μm ID) was used for sample trapping and cleaning. A 50cm long capillary column (75 μm ID; in-house packed using ReproSil-Pur C18-AQ 1.9 μm silica beads; Dr. Maisch) was then used for analytical separations at 250 nl/min over 90 min biphasic gradients. Acquisitions were performed through Top Speed Data-Dependent acquisition mode. First MS scans were acquired with a resolution of 240,000 (at 200 m/z) in the Lumos Fusion Orbitrap and a resolution of 120,000 (at 200 m/z) in the Lumos Fusion Orbitrap. The most intense parent ions were selected and fragmented by High energy Collision Dissociation (HCD) with a Normalized Collision Energy (NCE). Fragmented ions were acquired with a resolution 30,000 (at 200 m/z) in the orbitrap analyser and selected ions were then excluded for the following 20 s.

Raw data were processed using Mascot in Proteome Discoverer 2.4 against the human reference proteome (2020_06 and 75069 entries). Enzyme specificity was set to ArgC and AspN. Up to two missed cleavages were allowed and a 1% FDR cut-off was applied both at peptide and protein identification levels. For the database search, carbamidomethylation and HNE modifications were considered as variable modifications. The mass tolerance was set to 10 ppm for the precursors and 0.05 Da for the fragment ions. the Data was further processed and inspected in Scaffold 4.11.1 and in Proteome Discoverer 2.4.

Immunofluorescence (IF) assay:

Cells were grown to 70% confluence in 35 mm glass-bottomed dishes. For experiments involving ectopic expression of CDK9, cells were transfected with the plasmids indicated using TransIT 2020 reagent. T-REX[1-4] was performed as mentioned above. For most IF assays, cells were fixed by adding -20 °C pre-chilled MeOH and incubating at 4 °C for 10 min, at indicated time point post-irradiation by UV light. For all the time course assays for RNAPII phospho-Ser2 levels, cells were fixed at the indicated time point. Fixative was aspirated, and cells were washed twice with 1X DPBS (5 min incubations at room temperature), blocking and permeabilization were performed in one-step by incubation at 37 °C for 1 h in 1X DPBS containing 3% BSA and 0.2% Triton X-100. Cells were subsequently incubated with primary antibody (1:300, **Supplementary Table S5**) in incubation buffer (1% BSA, 0.02% Triton X-100 in 1X DPBS) for 2 h at room temperature. Cells were rinsed 3 times with 1X DPBS with 5 min incubation for each wash and subsequently incubated in dark for 1 h at room temperature with the corresponding second antibody (1:1000, **Supplementary Table S5**) in incubation buffer. Cells were rinsed 3 times with 1X DPBS with 5 min incubation for each rinse. DAPI was freshly prepared in 1X DPBS from 2 mg/mL wt./v stock solution in water and added to the wells at the final concentration of 0.2 µg/mL. The samples were incubated in dark for 3-5 min and washed once with 1X DPBS and stored at 4 °C in dark till images were taken, using either a Zeiss LSM 710 meta or LSM 700 confocal fluorescence microscope (Cornell fluorescence microscope imaging core facility and EPFL imaging and opitcs core facility). Image analysis was performed using Image-J (NIH, version 2.3.0). For IF-imaging experiments in native cell lines, similar protocol was deployed, but anti-CDK9 antibody (D7 clone, Santa-Cruz, **Supplementary Table S3**) was used, following successful validation by knockdown.

Western blotting:

Cells were lysed in 1X RIPA buffer containing in final concentrations 1 X Roche protease inhibitor, 1 mM sodium orthovanadate, and 1 mM PMSF, by rapid freeze-thaw (x3). Cell debris was removed, and the supernatant was collected after centrifugation at 18,000 x g for 8-10 min at 4 °C. Protein concentration was determined using Bradford assay with BSA as standard. 30–50 µg of total lysates were subjected to SDS-PAGE and the gel was transferred onto a PVDF membrane at 100 V for 1 h at 4 °C or at 40 V overnight at 4 °C. The membrane was blocked with 10% milk or 2% BSA in TBST buffer (20 mM Tris pH 7.6, 150 mM NaCl, 0.1% Tween 20) and probed with various antibodies at the indicated dilutions (**Supplementary Table S5**). Prior to probing for another protein with a different primary antibody *of different species origin*, the HRP activity from the preceding secondary antibodies was first inhibited by incubating the blot in 10 mM sodium azide-containing blocking buffer (10% milk or 2% BSA in TBST buffer)

over 3-5 h at room temperature or overnight at 4 °C. The azide-treated blots were then washed with TBST twice for the subsequent incubation with primary antibody of different species origin to the first one.

Construction of plasmids:

A ligase-free cloning method[1,7] was used to clone various plasmids (**Supplementary Table S1**) for expression in mammalian cells. To clone any desired fusion genes in any vector of choice, the gene of interest (GOI) was PCR-amplified out from the original plasmid using the indicated forward (fwd-1) and reverse primers (rev-1) in **Supplementary Table S1**. The resultant PCR product was extended using the indicated fwd-2, and rev-2 primers. The resultant "megaprimer" was inserted into the destination vector of interest that had been linearized with an appropriate restriction enzyme using PCR. The resulting crude mixture was transformed into *E. coli,* and the resulting *E. coli* colonies were amplified. The plasmid was verified by sequencing the entire inserted gene. Plasmids were purified using EZ-10 spin column plasmid DNA miniprep kits (Bio Basic, BS614).

The overexpression plasmids, Flag2-CDK9(wt) and Flag2-CDK9(C95A), were cloned into the IRES-based bicistronic pWPI GFP vector (Addgene #12254) by using Halo-TeV-Flag2-CDK9 and Halo-TeV-Flag2-CDK9 C95A mutant as template plasmids and the primer list shown in **Supplementary Table S1**. Synonymous mutations were incorporated in the region targeted by the gRNA selected for CDK9 to avoid recognition by the CRISPR-*Cas9* system. One of these mutations was incorporated in the protospacer adjacent motif (PAM) to avoid Cas9. The other three mutations were incorporated upstream of PAM (gRNA recognition site) within the first 10 nucleotides.

Generation of lentiviral-based knockdown lines:

HEK293T packaging cells were seeded and grown overnight in antibiotic-free media in 6 well plates. At 80% confluence, each well was transfected with packaging plasmid (pCMV-R8.74psPAX2, 500 ng), envelope plasmid (pCMV-VSV-G, 50 ng) and pLKO vector (sequences list in **Supplementary Table S2**, 500 ng) using TransIT.LT1 as per the manufacturer's protocol. After 18 h, media were removed and replaced with 20% serum-containing media. After 24 h, media containing viruses were collected, spun down and passed through a 0.45-micron filter and stored at –80 °C or used directly.

Cells in log phase were treated with 0.6 ml of virus supernatant (from above) in 8 µg/ml polybrene in a total of 6 ml of media in a 6-well plate. After 24 h, media were removed and replaced with media containing 2 µg/ml puromycin (which was completely toxic to all lines used in this study). Cells were cultured till plate was confluent, then cells were split and moved to a 10 cm dish in 2 µg/ml puromycin containing media

and grown again until reaching confluence. At this point the line was "selected", and target gene expression was analyzed by western blot and compared to shRNA controls.

Lentiviral-based Flag2-CDK9(wt) / Flag2-CDK9(C95A) overexpression in CDK9 KO HEK293T and subsequent cell sorting:

HEK293T cells were seeded in 6-well plate and incubated for 24 h. The cells were transfected with a mixture of 500 ng of packaging plasmid (pCMV-R8.74psPAX2), 50 ng of envelope plasmid (pCMV-VSV-G), and 500 ng of Flag2-CDK9 wt or Flag2-CDK9 C95A using TransIT-LT1 (Mirus Bio) following the manufacturer's protocol. 12 hours post transfection, cells were cultured with media containing 20% FBS for further 36 h. Media containing virus particles were harvested and used directly for infection below. HEK293T cells in 6-well plates were treated with 1 mL of virus containing media in a total volume of 6 mL of media containing 8 µg/mL polybrene. After 24 h, cells were cultured with fresh media until full confluence. Following selection, cells were sorted into low, medium-low, medium-high and high expression of GFP by fluorescence-activated cell sorting (FACS) (FACSAriaIII, SV Cytometry, EPFL). Sorted cells (250,000 cells per sorted cell group) were incubated in 12-well plates. Cells were then assayed by western blot.

Sorted populations of HEK293T cells were seeded in 10 cm dish and incubated for 24h. The cells were transfected with a mixture of 7.5 µg of packaging plasmid (pCMV-R8.74psPAX2), 750 ng of envelope plasmid (pCMV-VSV-G), and 7.5 µg of lentiCRISPR v2 gRNA (GenScript) targerting CDK9 using TransIT-LT1 (Mirus Bio) following the manufacturer's protocol. 48 h post transfection, 10 mL of supernatant was collected and filtered through 0.45-micron filter. To the filtered supernatant, 5.8 mL PBS, 325 µL 5 M NaCl, 2 mL PEG 8000 (20% in PBS), were added and the sample was incubated overnight at 4°C using end-over-end rotation. The supernatant was discarded post centrifugation at 1,500 x g for 1 h (4°C). The pelleted lentiviral particles were re-dissolved in 100 µL PBS.

Flag2-CDK9(wt)- or Flag2-CDK9(C95A)-overexpressing cells (subgroup of low expression of GFP, as described in **Figure S20D-E** and main manuscript) were seeded in 6-well plate and treated with 25 µL of the PBS solution containing lentiviral particles in a total volume of 2.5 mL of media containing 8 µg/mL polybrene. After 24 h, media were changed and the cells were incubated for further 24 h. Following this period, cells were incubated in media containing 2 µg/mL puromycin. Western blot analyses ensued as described elsewhere.

Quantitative real-time PCR (RT-qPCR):

RT-qPCR was carried out as previously described[2,11,12]. Total RNA was isolated from cells using Trizol reagent following the manufacturer's protocol. In order to validate the RNA preparation quality, the purity/integrity was assessed by agarose gel electrophoresis and concentration determined by A_{260nm} using a BioTek Cytation3 microplate reader with a Take3 plate. In principle, only the RNA preparation with two predominant intact ribosomal RNA (rRNA, 18S and 28S) bands with agarose gel and $A_{260nm}/A_{280nm} > 2$ with absorption analysis would be used for further RT-qPCR experiment. After validation, 1 µg of total RNA was incubated with Oligo(dT)$_{20}$ as a primer and Superscript III Reverse Transcriptase (Thermo Fisher) following the manufacturer's protocol. PCR was performed using the cDNA per biological replicate using iQ SYBR Green master mix solution (Bio-Rad) and primers specific priming for gene of interest (**Supplementary Table S11**) following the manufacturer's instruction. For genes with multiple splice variants, primers were chosen that can amplify conserved sequences across all splice variants if possible. Primer efficiencies for each specific gene were tested by titrating the cDNA (6-fold serial dilution with 5 different concentrations). Primers with a standard curve slope between -0.9 and 1.1 and $R^2 \geq 0.97$ were considered efficient. Single PCR products were validated by both melting curve analysis following the PCR protocol and agarose gel electrophoresis. Data were collected using a Thermo Fisher QS6 qPCR instrument. Threshold cycles were determined using the QS6 software. Samples with a threshold cycle >35 or without a single, correct melting point were not included in data analysis. The data analysis was done using the protocol previously described[12].

Statistical analysis, including the sample volume and corresponding *t*-test:

n for western blot represents the number of lanes on western blots under identical experimental conditions and each lane is from a separate individual replicate/experiment.

n for imaging experiments represent the number of single cells quantified from at least 7-8 fields of view with controls (empty vector controls for ectopically-overexpressed proteins, shRNA knockdown cell controls for endogenous proteins) shown in the figures. Multiple plates (n = 2) per condition were used when necessary, as in, for instance, Figure **2H**, and **3D.** All *t*-tests were two-tailed analysis. Both Pearson r and Spearman r have been computed by GraphPad Prism 8. The r- and p- values for both Pearson r and Spearman r analysis were computed using the means of protein expression level (x-axis) against every protein's targeting efficiency (phi, y-axis), with the assumption that all data followed Gaussian-distribution.

For Main Figures:

Figure 1C, n = 3 independent sets of biological replicates; *t*-test p = 3e-4.

Figure 2A, n = 3 independent sets of biological replicates; *t*-test: p = 3e-4 (Halo-TeV-Flag2-CDK9 (untagged/wt), light alone and untagged/wt, T-REX), p = 0.5 (untagged/wt, T-REX and Halo-TeV-NES-Flag2-CDK9 (NES), T-REX), p = 0.003 (untagged/wt, T-REX and Halo-TeV-NLS-Flag2-CDK9 (NLS), T-REX), p = 0.04 (NES, T-REX and NLS, T-REX), p = 2e-2 (untagged/wt, light alone and NES, T-REX), p = 8e-2 (untagged/wt, light alone and NLS, T-REX).

Figure 2B, n = 3 independent sets of biological replicates; *t*-test p = 7e-7.

Figure 2C, n = 4 independent sets of biological replicates; *t*-test: p = 0.0002 (Halo-TeV-Flag2-CDK9 expressed in shControl, T-REX and same construct expressed in shCyclinT1-35, T-REX), p = 0.0004 (Halo-TeV-Flag2-CDK9 expressed in shControl, T-REX and same construct expressed in shCyclinT1-36, T-REX), p = 0.038 (Halo-TeV-Flag2-CDK9 expressed in shCyclinT1-35, T-REX and same construct expressed in shCyclinT1-36, T-REX).

Figure 2D, n = 3 independent sets of biological replicates; *t*-test: p = 3e-6 (Halo-TeV-Flag2-CDK9 co-expressed with empty vector(EV), T-REX and Halo-TeV-Flag2-CDK9 co-expressed with HEXIM1(wt), T-REX), p = 0.09 (Halo-TeV-Flag2-CDK9 co-expressed with empty vector(EV), T-REX and Halo-TeV-Flag2-

CDK9 co-expressed with HEXIM1(Y203D), T-REX), p = 0.002 (Halo-TeV-Flag2-CDK9 co-expressed with HEXIM1(wt), T-REX and Halo-TeV-Flag2-CDK9 co-expressed with HEXIM1(Y203D), T-REX).

Figure 2E, n = 3 independent sets of biological replicates; *t*-test: p = 0.05 (Halo-TeV-Flag2-CDK9 expressed in shControl, T-REX and same construct expressed in HEXIM1-62, T-REX), p = 0.008 (Halo-TeV-Flag2-CDK9 expressed in shControl, T-REX and same construct expressed in shHEXIM1-63, T-REX), p = 0.01 (Halo-TeV-Flag2-CDK9 expressed in shControl, T-REX and same construct expressed in shHEXIM1-74, T-REX), p = 1e-1 (Halo-TeV-Flag2-CDK9 expressed in shHEXIM1-62, T-REX and same construct expressed in shHEXIM1-63, T-REX), p = 3e-1 (Halo-TeV-Flag2-CDK9 expressed in shHEXIM1-62, T-REX and same construct expressed in shHEXIM1-74, T-REX), p = 5e-1 (Halo-TeV-Flag2-CDK9 expressed in shHEXIM1-63, T-REX and same construct expressed in shHEXIM1-74, T-REX).

Figure 3B, n = 7 independent sets of biological replicates. *t*-test: p = 1e-2, Flag2-CDK9(C95A) / Flag2-CDK9(wt).

Figure 3C, n = 6 independent sets of biological replicates except the last time point n = 3; p = 9e-5 (5h and 0h)

Figure 3D, n = 402 (0h, T-REX), n = 300 (3h, T-REX), n = 553 (5h, T-REX), n = 642 (7h, T-REX), 467 (5h, light alone), 458 (5h, Ht-PreHNE alone), 575 (5h, DMSO), n = 336 (7h, light alone); *t*-test: p = 0.4497 (0h, T-REX and 3h, T-REX), p = 9e-113 (0h, T-REX and 5h, T-REX), p = 6e-162 (0h, T-REX and 7h, T-REX), p = 5e-123 (3h, T-REX and 5h, T-REX), p = 4e-73 (5h, T-REX and 5h, light alone), p = 1e-61 (5h, T-REX and 5h, Ht-PreHNE alone), p = 2e-68 (5h, T-REX and 5h, DMSO), p = 6e-80 (7h, T-REX and 7h, light alone).

Figure 4A, n = 5 independent sets of biological replicates; *t*-test between the indicated conditions and DMSO-treated cells in each transfected condition: p = 2e-14 (Halo-TeV-Flag2-CDK9 (untagged/wt), T-REX), p = 1.5e-1 (Halo-TeV-NES-Flag2-CDK9, T-REX), p= 9e-1 (Halo-TeV-NLS-Flag2-CDK9, T-REX), p = 2e-1 (Halo-TeV-Flag2-CDK9(C95A), T-REX), p = 5e-6 (Halo-TeV-Flag2-CDK9 (untagged/wt), NVP-2), p = 1e-2 (Halo-TeV-NES-Flag2-CDK9, NVP-2), p = 2e-5 (Halo-TeV-NLS-Flag2-CDK9, NVP-2), p = 6e-3 (Halo-TeV-Flag2-CDK9(C95A), NVP-2). *t*-tests: p = 1e-4 (Halo-TeV-Flag2-CDK9 (untagged/wt), T-REX and Halo-TeV-NES-Flag2-CDK9, T-REX), p = 8e-4 ((Halo-TeV-Flag2-CDK9 (untagged/wt), T-REX and Halo-TeV-NLS-Flag2-CDK9, T-REX), p = 2e-4 (Halo-TeV-Flag2-CDK9 (untagged/wt), T-REX and Halo-TeV-Flag2-CDK9(C95A), T-REX), p = 1e-1 (Halo-TeV-Flag2-CDK9 (untagged/wt), NVP-2 and NES, NVP-

2), p = 3e-1 (Halo-TeV-Flag2-CDK9 (untagged/wt), NVP-2 and Halo-TeV-NLS-Flag2-CDK9, NVP-2), p = 4e-1 (Halo-TeV-Flag2-CDK9 (untagged/wt), NVP-2 and Halo-TeV-Flag2-CDK9(C95A), NVP-2).

Figure 4B, n = 559 (Halo-TeV-Flag2-CDK9(untagged/wt), T-REX), n = 425 (Halo-TeV-Flag2-CDK9(untagged/wt), light alone), n = 474 (Halo-TeV-Flag2-CDK9(untagged/wt), Ht-PreHNE alone), n = 541 (Halo-TeV-Flag2-CDK9(untagged/wt), DMSO), n = 524 (Halo-TeV-NES-Flag2-CDK9, T-REX), n = 497 (Halo-TeV-NES-Flag2-CDK9, light alone), n = 499 (Halo-TeV-NES-Flag2-CDK9, Ht-PreHNE alone), n = 375 (Halo-TeV-NES-Flag2-CDK9, DMSO), n = 497 (Halo-TeV-NLS-Flag2-CDK9, T-REX), n = 394 (Halo-TeV-NLS-Flag2-CDK9, light alone), n = 405 (Halo-TeV-NLS-Flag2-CDK9, Ht-PreHNE alone), n = 378 (Halo-TeV-NLS-Flag2-CDK9, DMSO), n = 373 (Halo-TeV-Flag2-CDK9(untagged/wt), NVP-2 treatment), n = 530 (Halo-TeV-NES-Flag2-CDK9, NVP-2 treatment), n = 321 (Halo-TeV-NLS-Flag2-CDK9, NVP-2 treatment); *t*-tests: p = 1e-316 (Halo-TeV-Flag2-CDK9(untagged/wt), T-REX and Halo-TeV-Flag2-CDK9(untagged/wt), light alone), p = 3e-322 (Halo-TeV-Flag2-CDK9(untagged/wt), T-REX and Halo-TeV-Flag2-CDK9(untagged/wt), Ht-PreHNE alone), p = 2e-305 (Halo-TeV-Flag2-CDK9(untagged/wt), T-REX and Halo-TeV-Flag2-CDK9(untagged/wt), DMSO), p = 1e-165 (Halo-TeV-Flag2-CDK9(untagged/wt), T-REX and Halo-TeV-Flag2-CDK9(untagged/wt), NVP-2), p = 7e-589 (Halo-TeV-Flag2-CDK9(untagged/wt), NVP-2 and Halo-TeV-Flag2-CDK9(untagged/wt), DMSO), p = 1e-310 (Halo-TeV-NES-Flag2-CDK9, T-REX and Halo-TeV-NES-Flag2-CDK9, NVP-2), p = 8e-199 (Halo-TeV-NES-Flag2-CDK9, NVP-2 and Halo-TeV-NES-Flag2-CDK9, DMSO), p = 2e-139 (Halo-TeV-NLS-Flag2-CDK9, T-REX and Halo-TeV-NLS-Flag2-CDK9, NVP-2), p = 7e-159 (Halo-TeV-NLS-Flag2-CDK9, DMSO and Halo-TeV-NLS-Flag2-CDK9, NVP-2).

Figure 4C, n = 4 independent sets of biological replicates; *t*-test between the indicated conditions and DMSO-treated cells in each transfected condition: p = 4e-4 (Halo-TeV-Flag2-CDK9(untagged/wt), T-REX), p = 0.12 (Halo-TeV-NES-Flag2-CDK9, T-REX), p = 4e-1 (Halo-TeV-NLS-Flag2-CDK9, T-REX), p = 6e-1 (Halo-TeV-Flag2-CDK9(C95A), T-REX), p = 1e-3 (Halo-TeV-Flag2-CDK9(untagged/wt), NVP-2), p = 9e-5 (Halo-TeV-NES-Flag2-CDK9, NVP-2), p = 5e-3 (Halo-TeV-NLS-Flag2-CDK9, NVP-2), p = 3e-2 (Halo-TeV-Flag2-CDK9(C95A), NVP-2). *t*-test: p = 6e-3 (Halo-TeV-Flag2-CDK9(untagged/wt), T-REX and Halo-TeV-NES-Flag2-CDK9, T-REX), p = 3e-3 (Halo-TeV-Flag2-CDK9(untagged/wt), T-REX and Halo-TeV-NLS-Flag2-CDK9, T-REX), p = 2e-2 (Halo-TeV-Flag2-CDK9(untagged/wt), T-REX and Halo-TeV-Flag2-CDK9(C95A), T-REX), p = 5e-1 (Halo-TeV-Flag2-CDK9(untagged/wt), NVP-2 and Halo-TeV-NES-Flag2-CDK9, NVP-2), p = 0.95 (Halo-TeV-Flag2-CDK9(untagged/wt), NVP-2 and Halo-TeV-NLS-Flag2-CDK9, NVP-2), p = 3e-1 (Halo-TeV-Flag2-CDK9(untagged/wt), NVP-2 and Halo-TeV-Flag2-CDK9(C95A), NVP-2).

Figure 4D, n = 494 (Halo-TeV-Flag2-CDK9(wt), T-REX), n = 374 (Halo-TeV-Flag2-CDK9(wt), DMSO), n = 316 (Halo-TeV-Flag2-CDK9(C95A), T-REX), n = 266 (Halo-TeV-Flag2-CDK9(C95A), DMSO), n = 320 (Halo-TeV-Flag2-CDK9(wt), NVP-2 treatment), n = 338 (Halo-TeV-Flag2-CDK9(C95A), NVP-2 treatment); *t*-test: p = 2e-274 (Halo-TeV-Flag2-CDK9(untagged/wt), T-REX and Halo-TeV-Flag2-CDK9(untagged/wt), DMSO), p = 3e-256 (Halo-TeV-Flag2-CDK9(untagged/wt), T-REX and Halo-TeV-Flag2-CDK9(C95A), T-REX), p = 2e-274 (Halo-TeV-Flag2-CDK9(untagged/wt), T-REX and Halo-TeV-Flag2-CDK9(C95A), DMSO), p = 2e-64 (Halo-TeV-Flag2-CDK9(untagged/wt), T-REX and Halo-TeV-Flag2-CDK9(untagged/wt), NVP-2), p = 2e-4 (Halo-TeV-Flag2-CDK9(C95A), T-REX and Halo-TeV-Flag2-CDK9(C95A), DMSO), p = 8e-176 (Halo-TeV-Flag2-CDK9(C95A), T-REX and Halo-TeV-Flag2-CDK9(C95A), NVP-2), p = 6e-187 (Halo-TeV-Flag2-CDK9(C95A), DMSO and Halo-TeV-Flag2-CDK9(C95A), NVP-2).

Figure 4E, n = 8 independent sets of biological replicates; *t*-test between the indicated conditions: for the plot on the left, p = 9e-8 (Halo-TeV-Flag2-CDK9 (untagged/wt), T-REX and Halo-TeV-Flag2-CDK9 (untagged/wt), light alone), p = 4e-3 (Halo-TeV-Flag2-CDK9 (untagged/wt), T-REX and Halo-TeV-Flag2-CDK9 (untagged/wt), Ht-Pre-HNE alone), p = 1e-6 (Halo-TeV-Flag2-CDK9 (untagged/wt), T-REX and Halo-TeV-Flag2-CDK9 (untagged/wt), DMSO), p = 4e-4 (Halo-TeV-Flag2-CDK9 (untagged/wt), T-REX and Halo-TeV-Flag2-CDK9 (C95A), Ht-Pre-HNE alone), p = 8e-2 (Halo-TeV-Flag2-CDK9 (C95A), T-REX and Halo-TeV-Flag2-CDK9 (C95A), light alone), p = 2e-2 (Halo-TeV-Flag2-CDK9 (C95A), T-REX and Halo-TeV-Flag2-CDK9 (C95A), light alone), p = 6e-2 (Halo-TeV-Flag2-CDK9 (C95A), T-REX and Halo-TeV-Flag2-CDK9 (C95A), DMSO).

For the plot in the middle, p = 1e-3 (Halo-TeV-Flag2-CDK9 (untagged/wt), T-REX and Halo-TeV-Flag2-CDK9 (untagged/wt), light alone), p = 2e-3 (Halo-TeV-Flag2-CDK9 (untagged/wt), T-REX and Halo-TeV-Flag2-CDK9 (untagged/wt), Ht-Pre-HNE alone), p = 6e-4 (Halo-TeV-Flag2-CDK9 (untagged/wt), T-REX and Halo-TeV-Flag2-CDK9 (untagged/wt), DMSO), p = 4e-3 (Halo-TeV-Flag2-CDK9 (untagged/wt), T-REX and Halo-TeV-Flag2-CDK9 (C95A), Ht-Pre-HNE alone), p = 3e-1 (Halo-TeV-Flag2-CDK9 (C95A), T-REX and Halo-TeV-Flag2-CDK9 (C95A), light alone), p = 2e-1 (Halo-TeV-Flag2-CDK9 (C95A), T-REX and Halo-TeV-Flag2-CDK9 (C95A), light alone), p = 1e-1 (Halo-TeV-Flag2-CDK9 (C95A), T-REX and Halo-TeV-Flag2-CDK9 (C95A), DMSO).

For the plot on the right, p = 5e-5 (Halo-TeV-Flag2-CDK9 (untagged/wt), T-REX and Halo-TeV-Flag2-CDK9 (untagged/wt), light alone), p = 2e-2 (Halo-TeV-Flag2-CDK9 (untagged/wt), T-REX and Halo-TeV-Flag2-CDK9 (untagged/wt), Ht-Pre-HNE alone), p = 2e-4 (Halo-TeV-Flag2-CDK9 (untagged/wt), T-REX and Halo-TeV-Flag2-CDK9 (untagged/wt), DMSO), p = 5e-4 (Halo-TeV-Flag2-CDK9 (untagged/wt), T-

REX and Halo-TeV-Flag2-CDK9 (C95A), Ht-Pre-HNE alone), p = 2e-1 (Halo-TeV-Flag2-CDK9 (C95A), T-REX and Halo-TeV-Flag2-CDK9 (C95A), light alone), p = 5e-1 (Halo-TeV-Flag2-CDK9 (C95A), T-REX and Halo-TeV-Flag2-CDK9 (C95A), light alone), p = 2e-2 (Halo-TeV-Flag2-CDK9 (C95A), T-REX and Halo-TeV-Flag2-CDK9 (C95A), DMSO).

For Supplementary Figures:

Figure S1D, n = 229 (MOMLS-Halo), n = 134 (NLS-Halo). *t*-test between the two conditions p = 1.6e-1.

Figure S1F, n = 3 independent sets of biological replicates. *t*-test between the two conditions p = 8e-1.

Figure S5B, n = 3 independent sets of biological replicates.

Figure S6B, n = 568 (HaloATIC), n = 865 (HaloATP6V1A), n = 431 [HaloCDK9(Halo-TeV-Flag2-CDK9(untagged/wt))], n = 749 (HaloDAGLB), n = 476 (HaloEPHX1), n = 457 (HaloFKBP8), n = 764 (HaloLCMT1), n = 915 (HaloLTA4H), n = 867 (HaloRCC1).

Figure S7B, n = 4 (HaloATIC), n = 3 (HaloEPHX1), n = 4 (HaloFKBP8), n = 3 (HaloGGCT), n = 6 (HaloLCMT1), n = 4 (HaloLTA4H), n = 6 (HaloRCC1).

Figure S7C, for targeting efficiency (y-axis), n = 4 (HaloATIC), n = 3 [HaloCDK9(Halo-TeV-Flag2-CDK9(untagged/wt))], n = 3 (HaloEPHX1), n = 4 (HaloFKBP8), n = 3 (HaloGGCT), n = 6 (HaloLCMT1), n = 4 (HaloLTA4H), n = 6 (HaloRCC1); for HaloPOI expression level, n = 3 independent sets of biological replicates;

Figure S8B, n = 431 (Halo-TeV-Flag2-CDK9(wt)), n = 275 (Halo-TeV-NES-Flag2-CDK9), n = 227 (Halo-TeV-NLS-Flag2-CDK9), n = 396 (Halo-TeV-Flag2-CDK9(C95A)), n = 472 (FLAG-CDK9); *t*-test p = 2e-20 (Halo-TeV-Flag2-CDK9(untagged/wt) and Halo-TeV-NES-Flag2-CDK9), p = 2e-30 (Halo-TeV-Flag2-CDK9(untagged/wt) and Halo-TeV-NLS-Flag2-CDK9), p = 0.002 (Halo-TeV-Flag2-CDK9(untagged/wt) and Halo-TeV-Flag2-CDK9(C95A)), p = 0.137 (Halo-TeV-Flag2-CDK9(untagged/wt) and Flag-CDK9(no HaloTag)).

Figure S9A, n = 3 independent sets of biological replicates. *t*-test between the indicated conditions: p = 8e-3 (Halo-TeV-Flag2-DAGLB(untagged) and Halo-TeV-NES-Flag2-DAGLB), p = 4e-6 (Halo-TeV-Flag2-DAGLB(untagged) and Halo-TeV-NLS-Flag2-DAGLB), p = 5e-4 (Halo-TeV-Flag2-NES-DAGLB and Halo-TeV-NLS-Flag2-DAGLB).

Figure S9B, n = 3 independent sets of biological replicates. *t*-test between the indicated conditions: p = 2e-4 (Halo-TeV-Flag2-DAGLB(untagged) and Halo-TeV-NES-Flag2-DAGLB), p = 9e-7 (Halo-TeV-Flag2-

DAGLB(untagged) and Halo-TeV-NLS-Flag2-DAGLB), p = 9e-3 (Halo-TeV-Flag2-NES-DAGLB and Halo-TeV-NLS-Flag2-DAGLB).

Figure S10A, n = 3 independent sets of biological replicates. *t*-test between the indicated conditions: p = 5.2e-2 (Halo-TeV-Flag2-GGCT(untagged) and Halo-TeV-NES-Flag2-GGCT), p = 3e-2 (Halo-TeV-Flag2-GGCT(untagged) and Halo-TeV-NLS-Flag2-GGCT), p = 2.5e-2 (Halo-TeV-Flag2-NES-GGCT and Halo-TeV-NLS-Flag2-GGCT).

Figure S10B, n = 3 independent sets of biological replicates. *t*-test between the indicated conditions: p = 9e-3 (Halo-TeV-Flag2-GGCT(untagged) and Halo-TeV-NES-Flag2-GGCT), p = 2.7e-7 (Halo-TeV-Flag2-GGCT(untagged) and Halo-TeV-NLS-Flag2-GGCT), p = 3e-2 (Halo-TeV-Flag2-NES-GGCT and Halo-TeV-NLS-Flag2-GGCT).

Figure S11A, n = 3 independent sets of biological replicates. *t*-test between the indicated conditions: p = 2e-1 (Halo-TeV-Flag2-LCMT1(untagged) and Halo-TeV-NES-Flag2-LCMT1), p = 2e-7 (Halo-TeV-Flag2-LCMT1(untagged) and Halo-TeV-NLS-Flag2-LCMT1), p = 4.5e-3 (Halo-TeV-Flag2-NES-LCMT1 and Halo-TeV-NLS-Flag2-LCMT1).

Figure S11B, n = 3 independent sets of biological replicates. *t*-test between the indicated conditions: p = 2e-2 (Halo-TeV-Flag2-LCMT1(untagged) and Halo-TeV-NES-Flag2-LCMT1), p = 6.5e-3 (Halo-TeV-Flag2-LCMT1(untagged) and Halo-TeV-NLS-Flag2-LCMT1), p = 4.4e-1 (Halo-TeV-Flag2-NES-LCMT1 and Halo-TeV-NLS-Flag2-LCMT1).

Figure S12A, n = 3 independent sets of biological replicates. *t*-test between the indicated conditions: p = 2e-5 (Halo-TeV-Flag2-RCC1(untagged) and Halo-TeV-NES-Flag2-RCC1), p = 1.2e-7 (Halo-TeV-Flag2-RCC1(untagged) and Halo-TeV-NLS-Flag2-RCC1), p = 6.3e-5 (Halo-TeV-Flag2-NES-RCC1 and Halo-TeV-NLS-Flag2-RCC1).

Figure S12B, n = 3 independent sets of biological replicates. *t*-test between the indicated conditions: p = 2e-3 (Halo-TeV-Flag2-RCC1(untagged) and Halo-TeV-NES-Flag2-RCC1), p = 3e-7 (Halo-TeV-Flag2-RCC1(untagged) and Halo-TeV-NLS-Flag2-RCC1), p = 7e-3 (Halo-TeV-Flag2-NES-RCC1 and Halo-TeV-NLS-Flag2-RCC1).

Figure S13A, n = 422 (Halo-TeV-Flag2-EPHX1(untagged)), n = 419 (Halo-TeV-NES-Flag2-EPHX1), n = 77 (Halo-TeV-NLS-Flag2-EPHX1). *t*-test between the indicated conditions: p = 6.9e-15 (Halo-TeV-Flag2-EPHX1(untagged) and Halo-TeV-NES-Flag2-EPHX1), p = 1.2e-22 (Halo-TeV-Flag2-EPHX1(untagged) and Halo-TeV-NLS-Flag2-EPHX1), p = 1.3e-25 (Halo-TeV-Flag2-NES-EPHX1 and Halo-TeV-NLS-Flag2-EPHX1).

Figure S13B, n = 3 independent sets of biological replicates. *t*-test between the indicated conditions: p = 1.5e-4 (Halo-TeV-Flag2-EPHX1(untagged) and Halo-TeV-NES-Flag2-EPHX1), p = 1.3e-1 (Halo-TeV-Flag2-EPHX1(untagged) and Halo-TeV-NLS-Flag2-EPHX1), p = 6.7e-3 (Halo-TeV-Flag2-NES-EPHX1 and Halo-TeV-NLS-Flag2-EPHX1).

Figure S13C, n = 3 independent sets of biological replicates. *t*-test between the indicated conditions: p = 3.1e-10 (Halo-TeV-Flag2-EPHX1(untagged) and Halo-TeV-NES-Flag2-EPHX1), p = 2.3e-9 (Halo-TeV-Flag2-EPHX1(untagged) and Halo-TeV-NLS-Flag2-EPHX1), p = 1.2e-5 (Halo-TeV-Flag2-NES-EPHX1 and Halo-TeV-NLS-Flag2-EPHX1).

Figure S14A, n = 3 independent sets of biological replicates.

Figure S14B, n = 6 independent sets of biological replicates, except for the cohort of Halo-TeV-Flag2-CDK9(C95A), n = 3. *t*-test between the indicated conditions: for the quantification of Halo-CDK9 band intensities, p = 4.5e-2 (Halo-TeV-Flag2-CDK9(untagged/wt) and (Halo-TeV-NES-Flag2-CDK9), p = 1.0e-8 (Halo-TeV-Flag2-CDK9(untagged/wt) and Halo-TeV-NLS-Flag2-CDK9), p = 2.9e-9 (Halo-TeV-Flag2-CDK9(untagged/wt) and Halo-TeV-Flag2-CDK9(C95A)), p = 1.4e-4 (Halo-TeV-NES-Flag2-CDK9 and Halo-TeV-NLS-Flag2-CDK9).

For the quantification of endogenous CDK9 band intensities in the corresponding Halo-CDK9 expressing cells, p = 1.6e-2 (Halo-TeV-Flag2-CDK9(untagged/wt) and Halo-TeV-NES-Flag2-CDK9), p = 3.2e-3 (Halo-TeV-Flag2-CDK9(untagged/wt) and Halo-TeV-NLS-Flag2-CDK9), p = 5.1e-3 (Halo-TeV-Flag2-CDK9(untagged/wt) and Halo-TeV-Flag2-CDK9(C95A)), p = 4.1e-4 (Halo-TeV-NES-Flag2-CDK9 and Halo-TeV-NLS-Flag2-CDK9).

Figure S15A, n = 3 independent sets of biological replicates.

Figure S15B, n = 3 independent sets of biological replicates. *t*-test between the indicated conditions: p = 3.3e-1 (Halo-TeV-Flag2-CDK9(untagged), T-REX and Halo-TeV-Flag2-CDK9(untagged), HNE bolus dosing), p = 3.2e-3 (Halo-TeV-Flag2-DAGLB(untagged), T-REX and Halo-TeV-Flag2-DAGLB(untagged), HNE bolus dosing), p = 1.8e-1 (Halo-TeV-Flag2-GGCT(untagged), T-REX and Halo-TeV-Flag2-GGCT(untagged), HNE bolus dosing), p = 6.3e-3 (Halo-TeV-Flag2-LCMT1(untagged), T-REX and Halo-TeV-Flag2-LCMT1(untagged), HNE bolus dosing), p = 1e-2 (Halo-TeV-Flag2-RCC1(untagged), T-REX and Halo-TeV-Flag2-RCC1(untagged), HNE bolus dosing), p = 5.5e-2 (Halo-TeV-Flag2-EPHX1(untagged), T-REX and Halo-TeV-Flag2-EPHX1(untagged), HNE bolus dosing).

Figure S15C, n = 3 independent sets of biological replicates.

Figure S15D, n = 1079 ((Halo-TeV-Flag2-CDK9(untagged), 0h), n = 107 ((Halo-TeV-Flag2-CDK9(untagged), 8h), n = 250 ((Halo-TeV-Flag2-RCC1(untagged), 0h), n = 165 ((Halo-TeV-Flag2-RCC1(untagged), 8h), n = 238 ((Halo-TeV-Flag2-GGCT(untagged), 0h), n = 275 ((Halo-TeV-Flag2-GGCT(untagged), 8h), n = 454 ((Halo-TeV-Flag2-LCMT1(untagged), 0h), n = 400 ((Halo-TeV-Flag2-LCMT1(untagged), 8h), n = 160 ((Halo-TeV-Flag2-DAGLB(untagged), 0h), n = 179 ((Halo-TeV-Flag2-DAGLB(untagged), 8h), n = 217 ((Halo-TeV-Flag2-EPHX1(untagged), 0h), n = 194 ((Halo-TeV-Flag2-EPHX1(untagged), 8h).

t-test between the indicated conditions: p = 3e-53 ((Halo-TeV-Flag2-CDK9(untagged), 0h and (Halo-TeV-Flag2-CDK9(untagged), 8h), p = 3.4e-4 ((Halo-TeV-Flag2-DAGLB(untagged), 0h and (Halo-TeV-Flag2-DAGLB(untagged), 8h), p = 1.5e-3 ((Halo-TeV-Flag2-EPHX1(untagged), 0h and (Halo-TeV-Flag2-EPHX1(untagged), 8h), p = 8e-2 ((Halo-TeV-Flag2-GGCT(untagged), 0h and (Halo-TeV-Flag2-GGCT(untagged), 8h), p = 2.1e-8 ((Halo-TeV-Flag2-LCMT1(untagged), 0h and (Halo-TeV-Flag2-LCMT1(untagged), 8h), p = 8e-2 ((Halo-TeV-Flag2-RCC1(untagged), 0h and (Halo-TeV-Flag2-RCC1(untagged), 8h).

Figure S16B, for Halo-TeV-Flag2-CDK9(untagged/wt), n = 1079 (0h), n = 648 (0.5h), n = 347 (1h), n = 336 (1.5h), n = 862 (2h), n = 141 (4h), n = 107 (8h). For Flag-CDK9, n = 578 (0h), n = 115 (2h), n = 128 (4h), n = 82 (8h).

Figure S17A, n = 3 independent sets of biological replicates. *t*-test p = 3e-1 (Halo-TeV-Flag2-CDK9(untagged/wt) and Halo-TeV-NES-Flag2-CDK9), p = 2e-1 (Halo-TeV-Flag2-CDK9(untagged/wt) and Halo-TeV-NLS-Flag2-CDK9), p = 3e-1 (Halo-TeV-Flag2-CDK9(untagged/wt) and Halo-TeV-Flag2-CDK9(C95A)), p = 3e-1 (Halo-TeV-Flag2-CDK9(untagged/wt) and empty vector).

Figure S17B, n = 3 independent sets of biological replicates (both middle and right panel). For left panel, *t*-test p = 2e-3 (shControl and shCDK9-92), p = 4e-3 (shControl and shCDK9-94), p = 7e-3 (shControl and shCDK9-97). For right panel, p = 2e-2 (shControl and shCDK9-92), p = 5e-9 (shControl and shCDK9-94), p = 3e-2 (shControl and shCDK9-97).

Figure S17C, n = 882 (Halo-TeV-Flag2-CDK9(wt/untagged) with HA-CyclinT1), n = 429 (Halo-TeV-Flag2-CDK9(untagged/wt) with empty vector); *t*-test p = 3e-45 between these two conditions.

Figure S17E, n = 970 (siRNA knockdown control), n = 479 (siCyclinT1 -1), n = 524 (siCyclinT1 -2), n = 621 (siCyclinT1 -3); *t*-test p = 1e-53 [Halo-TeV-Flag2-CDK9(untagged/wt) co-expressed with si-Control and Halo-TeV-Flag2-CDK9(untagged/wt) co-expressed with si-CyclinT1(1)], p = 6e-23 [Halo-TeV-Flag2-CDK9(untagged/wt) co-expressed with si-Control and Halo-TeV-Flag2-CDK9(untagged/wt) co-expressed

with si-CyclinT1(2)], p = 5e-136 [Halo-TeV-Flag2-CDK9(untagged/wt) co-expressed with si-Control and Halo-TeV-Flag2-CDK9(untagged/wt) co-expressed with si-CyclinT1(3)].

Figure S17F, n = 6 except for shCyclinT1 lines 28, 29, and 17 and shCDK9 lines 92, 97, and 94 (n = 2). *t*-test between the indicated conditions: p = 1.7e-2 (shControl and shCyclinT1 line 35), p = 3.8e-2 (shControl and shCyclinT1 line 36), p = 5.6e-3 (shControl and shCyclinT1 lines 74).

Figure S18A, n = 4 independent sets of biological replicates; *t*-test p = 2e-9 (Halo-TeV-Flag2-CDK9(untagged/wt), T-REX and Halo-TeV-NLS-Flag2-CDK9, T-REX both in shControl line), p = 0.29 (Halo-TeV-Flag2-CDK9(untagged/wt), T-REX in shControl line and Halo-TeV-NLS-Flag2-CDK9, T-REX in shCyclinT1-35), p = 0.46 (Halo-TeV-Flag2-CDK9(untagged/wt), T-REX in shControl line and Halo-TeV-NLS-Flag2-CDK9, T-REX in shCyclinT1-36), p = 0.01 (Halo-TeV-NLS-Flag2-CDK9 in shControl line and Halo-TeV-NLS-Flag2-CDK9 in shCyclinT1-35), p = 0.002 (Halo-TeV-NLS-Flag2-CDK9 in shControl line and Halo-TeV-NLS-Flag2-CDK9 in shCyclinT1-36).

Figure S18B, n = 3 independent sets of biological replicates. *t*-test between the indicated conditions: for the quantification of Halo-CDK9 band intensities, p = 5e-3 (Halo-TeV-Flag2-CDK9(untagged/wt), shControl and Halo-TeV-Flag2-CDK9(untagged/wt), shCyclinT1-35), p = 5e-1 (Halo-TeV-Flag2-CDK9(untagged/wt), shCyclinT1-35 and Halo-TeV-NES-Flag2-CDK9, shCyclinT1-35), p = 1e-2 (Halo-TeV-Flag2-CDK9(untagged/wt), shCyclinT1-35 and Halo-TeV-NLS-Flag2-CDK9, shCyclinT1-35), p = 1e-1 (Halo-TeV-NES-Flag2-CDK9, shCyclinT1-35 and Halo-TeV-NLS-Flag2-CDK9, shCyclinT1-35), p = 5e-3 (Halo-TeV-NLS-Flag2-CDK9, shCyclinT1-35 and Halo-TeV-Flag2-CDK9(C95A), shCyclinT1-35).

For the quantification of endogenous CDK9 band intensities in the corresponding Halo-CDK9 expressing cells, p = 8e-1 (Halo-TeV-Flag2-CDK9(untagged/wt), shControl and Halo-TeV-Flag2-CDK9(untagged/wt), shCyclinT1-35), p = 2e-2 (Halo-TeV-Flag2-CDK9(untagged/wt), shCyclinT1-35 and Halo-TeV-NES-Flag2-CDK9, shCyclinT1-35), p = 2e-1 (Halo-TeV-Flag2-CDK9(untagged/wt), shCyclinT1-35 and Halo-TeV-NLS-Flag2-CDK9, shCyclinT1-35), p = 3e-1 (Halo-TeV-NES-Flag2-CDK9, shCyclinT1-35 and Halo-TeV-NLS-Flag2-CDK9, shCyclinT1-35), p = 3e-2 (Halo-TeV-NLS-Flag2-CDK9, shCyclinT1-35 and Halo-TeV-Flag2-CDK9(C95A), shCyclinT1-35).

Figure S20B, n = 6 for the first and the third bars, n = 4 for the second bar, n = 3 for the last bar; *t*-test p = 2e-7 (Halo-TeV-Flag2-CDK9(untagged/wt) with empty vector and Halo-TeV-Flag2-CDK9(C95A) with EV), p = 4e-9 (Halo-TeV-Flag2-CDK9(untagged/wt) with empty vector and Halo-TeV-Flag2-CDK9(untagged/wt) with CyclinT1), p = 9e-7 (Halo-TeV-Flag2-CDK9(untagged/wt) with empty vector and Halo-TeV-Flag2-CDK9(C95A) with CyclinT1), p = 0.18 (Halo-TeV-Flag2-CDK9(C95A) with empty vector

and Halo-TeV-Flag2-CDK9(untagged/wt) with CyclinT1), p = 0.24 (Halo-TeV-Flag2-CDK9(untagged/wt) with CyclinT1 and Halo-TeV-Flag2-CDK9(C95A) with CyclinT1).

For the ***inset*** graph, n = 6 for EV and n = 3 for CyclinT1, *t*-test p = 9e-3**.**

Figure S20C, n = 3 independent sets of biological replicates for HEK293T CDK9 KO+Flag2-CDK9 wt and CDK9 KO+Flag2-CDK9 C95A.

Figure S21C, n = 16 independent sets of biological replicates for RNAPII CTD phospho-Ser2 data (left panel); n = 11 independent sets of biological replicates for RNAPII CTD phospho-Ser5 data (right panel). For the left panel, *t*-test p = 9e-23 (T-REX and DMSO), p = 2e-20 (DMSO and NVP-2), p = 7e-7 (T-REX and NVP-2). For the right panel, p = 5e-6 (T-REX and DMSO), p = 7e-9 (DMSO and NVP-2), p = 0.0008 (T-REX and NVP-2).

Figure S22A, n = 45 (siControl cytoplasm), n = 45 (siControl nucleus), n = 56 (siCDK9 cytoplasm), n = 56 (siCDK9 cytoplasm). *t*-test: p = 4e-3 (siControl cytoplasm and siCDK9 cytoplasm), p < 1e-4 (siControl nucleus and siCDK9 nucleus).

Figure S22B, n = 224 (HEK293T), n = 45 (HeLa), n = 95 (U2OS), n = 100 (RAW 264.7)

Figure S22C, n = 224 (DMSO), n = 108 (HNE), n = 117 (Leptomycin B). *t*-test: p < 1e-6 (DMSO and HNE), p < 1e-6 (DMSO and Leptomycin B), p = 2e-3 (HNE and Leptomycin B).

Figure S23, n = 5 independent sets of biological replicates; *t*-test p = 2e-7 (Halo-TeV-Flag2-CDK9(untagged/wt), T-REX and Halo-TeV-Flag2-CDK9(untagged/wt), DMSO), p = 1e-5 (Halo-TeV-Flag2-CDK9(untagged/wt), T-REX and Halo-TeV-Flag2-CDK9(untagged/wt), CIP treatment), p = 6e-13 (Halo-TeV-Flag2-CDK9(untagged/wt), DMSO and Halo-TeV-Flag2-CDK9(untagged/wt), CIP treatment), p = 0.0004 (Halo-TeV-Flag2-CDK9(untagged/wt), T-REX and Halo-TeV-Flag2-CDK9(untagged/wt), NVP-2), p = 2e-1 (Halo-TeV-Flag2-CDK9(untagged/wt), DMSO and Halo-TeV-NES-Flag2-CDK9, DMSO), p = 9e-1 (Halo-TeV-Flag2-CDK9(untagged/wt), DMSO and Halo-TeV-NLS-Flag2-CDK9, DMSO), p = 7e-1 (Halo-TeV-Flag2-CDK9(untagged/wt), DMSO and Halo-TeV-Flag2-CDK9(C95A), DMSO), p = 7e-2 (Halo-TeV-Flag2-CDK9(untagged/wt), DMSO and Halo-TeV-Flag2-CDK9(untagged/wt) with CyclinT1, DMSO), p = 7e-1 (Halo-TeV-Flag2-CDK9(untagged/wt), DMSO and empty vector, DMSO).

Figure S24A, n = 4 independent sets of biological replicates.

Figure S24B, for the plot on the left, n = 8 independent sets of biological replicates. *t*-test p = 8e-3 (Halo-TeV-Flag2-CDK9(untagged/wt), T-REX and Halo-TeV-Flag2-CDK9(untagged/wt), light alone), p = 1e-2 (Halo-TeV-Flag2-CDK9(untagged/wt), T-REX and Halo-TeV-Flag2-CDK9(untagged/wt), Ht-PreHNE

alone), p = 1e-4 (Halo-TeV-Flag2-CDK9(untagged/wt), T-REX and Halo-TeV-Flag2-CDK9(untagged/wt), DMSO), p = 2e-2 (Halo-TeV-Flag2-CDK9(untagged/wt), T-REX and Halo-TeV-Flag2-CDK9(C95A), T-REX), p = 1e-1 (Halo-TeV-Flag2-CDK9(C95A), T-REX and Halo-TeV-Flag2-CDK9(C95A), light alone), p = 2e-1 (Halo-TeV-Flag2-CDK9(C95A), T-REX and Halo-TeV-Flag2-CDK9(C95A), Ht-PreHNE alone), p = 2e-1 (Halo-TeV-Flag2-CDK9(C95A), T-REX and Halo-TeV-Flag2-CDK9(C95A), DMSO).

For the plot on the right, n = 4 independent sets of biological replicates. *t*-test p = 3e-2 (Halo-TeV-Flag2-CDK9(untagged/wt), T-REX and Halo-TeV-Flag2-CDK9(untagged/wt), light alone), p = 5e-3 (Halo-TeV-Flag2-CDK9(untagged/wt), T-REX and Halo-TeV-Flag2-CDK9(untagged/wt), Ht-PreHNE alone), p = 6e-3 (Halo-TeV-Flag2-CDK9(untagged/wt), T-REX and Halo-TeV-Flag2-CDK9(untagged/wt), DMSO), p = 4e-3 (Halo-TeV-Flag2-CDK9(untagged/wt), T-REX and Halo-TeV-Flag2-CDK9(C95A), T-REX), p = 1e-1 (Halo-TeV-Flag2-CDK9(C95A), T-REX and Halo-TeV-Flag2-CDK9(C95A), light alone), p = 2e-1 (Halo-TeV-Flag2-CDK9(C95A), T-REX and Halo-TeV-Flag2-CDK9(C95A), Ht-PreHNE alone), p = 7e-1 (Halo-TeV-Flag2-CDK9(C95A), T-REX and Halo-TeV-Flag2-CDK9(C95A), DMSO).

Supplementary datasets

Dataset S1: Proteomics data for local-specific protein targets identified from Localis-rex obtained under the experimental setup and conditions described in the manuscript. Relate to **Fig.S3D.**

See 3 Excel files, corresponding to S1-1, S1-2, and S1-3. For correlation between the abbreviated protein names used in the paper and part of the names used in the Excel files, see table below and Fig.S3D.

S1-1: Localis-rex locale-specific protein targets list-1: the first tab labeled as the entire list corresponds to the list of all proteins after removal of keratin/keratin-like proteins and proteins without H/L ratios; the second tab features the targets either ranked top 16% or bottom 16% (i.e., either > 1σ or < -1σ)

S1-2: Localis-rex locale-specific protein targets list-2: the first tab labeled as the entire list corresponds to the list of all proteins after removal of keratin/keratin-like proteins and proteins without H/L ratios; the second tab features the targets either ranked top 16% or bottom 16% (i.e., either > 1σ or < -1σ)

S1-3: Localis-rex locale-specific protein targets list-3: the first tab labeled as the entire list corresponds to the list of all proteins after removal of keratin/keratin-like proteins and proteins without H/L ratios; the second tab features the targets either ranked top 16% or bottom 16% (i.e., either > 1σ or < -1σ)

Analysis for protein targets from **Group-1** (Hits 2X, Null 1X) (See **Fig.S3D**): denoting proteins enriched when hydroxynonenal was released within the same locale in two replicates [with heavy:light ratio either > +1σ or < -1σ, (either ranked top 16% or bottom 16%)], but these proteins were missing altogether in the remaining replicate. **In Red:** protein is a hit (either > 1σ or < -1σ); **n/a:** protein is not in the indicated replicate data set. *Confirmed as FAM49B, by blasting the 2 identified unique peptides ("DAEGILEDLQSYR" and "INNVPAEGENEVNNELANR") against the entire human proteome.*

	Abbreviation	(Partial) Name in S1-1	(Partial) Name in S1-2	(Partial) Name in S1-3
1	ACO2	n/a	…Aconitate hydratase, mito.	Aconitate hydratase, mito.
2	ATIC	5-aminoimidazole-4-carboxamide ribonucleotide formyltransferase/IMP cyclohydrolase	5-aminoimidazole-4-carboxamide ribonucleotide formyltransferase…	n/a
3	CDK9	….cyclin-dependent kinase 9	….cyclin-dependent kinase 9	n/a
4	DAGLB	….diacylglycerol lipase beta	….diacylglycerol lipase beta	n/a
5	FAM49B	n/a	*Putative uncharacterized protein DKFZp686B04128	Protein FAM49B
6	GGCT	n/a	γ-glutamylcyclotransferase	γ-glutamylcyclotransferase
7	IPO7	n/a	Importin-7	Importin-7
8	LCMT1	Leucine carboxyl methyltransferase 1	n/a	Leucine carboxyl methyltransferase 1
9	NAPA	n/a	Alpha-soluble NSF attachment..	Alpha-soluble NSF attachment…

Analysis for protein targets from **Group-2** (hits 2X, middle 1X) (See **Fig.S3D**): denoting proteins enriched when hydroxynonenal was released within the same locale in two replicates (with heavy:light ratio either > +1σ or < -1σ), but these proteins appeared in the middle (or unenriched) group in the remaining replicate. **In Red:** protein is a hit (either > 1σ or < -1σ); **In Blue:** protein is not a hit (neither > 1σ nor < -1σ).

	Abbreviation	(Partial) Name in S1-1	(Partial) Name in S1-2	(Partial) Name in S1-3
10	ATP6V1A	V-type proton ATPase catalytic subunit A	V-type proton ATPase catalytic subunit A	V-type proton ATPase catalytic subunit A
11	CALR	… Calreticulin	… Calreticulin	Calreticulin
12	CKMT1A	Creatine kinase U-type, mitochondrial	Creatine kinase U-type, mitochondrial	Creatine kinase U-type, mitochondrial
13	DDX17	DEAD (Asp-Glu-Ala-Asp) box polypeptide 17	DEAD (Asp-Glu-Ala-Asp) box polypeptide 17	DEAD (Asp-Glu-Ala-Asp) box polypeptide 17
14	EPHX1	cDNA, FLJ93975,…(EPHX1)	cDNA, FLJ93975,…(EPHX1)	cDNA, FLJ93975,…(EPHX1)
15	FKBP8	cDNA, FLJ93891…(FKBP8),…	cDNA, FLJ93891…(FKBP8),…	cDNA, FLJ93891…(FKBP8),…

16	KCTD12	cDNA FLJ46506…(KCTD12),…	cDNA FLJ46506…(KCTD12),…	cDNA FLJ46506…(KCTD12),…
17	PCCA	cDNA, FLJ96567…propionyl Coenzyme A carboxylase (PCCA)	cDNA, FLJ56469… Propionyl-CoA carboxylase alpha chain…	Propionyl-CoA carboxylase alpha chain, mitochondrial
18	SCCPDH	Saccharopine dehydrogenase-like ….	Saccharopine dehydrogenase….	Saccharopine dehydrogenase-like
19	SLC25A13	cDNA FLJ54671… Aralar2	cDNA FLJ54671… Aralar2	…Aralar2
20	TUFM	Elongation factor Tu, mitochon.	Elongation factor Tu, mitochon.	Elongation factor Tu, mitochon.
21	RCC1	Regulator of chsm. condensation	Regulator of chsm. condensation	Regulator of chsm. condensation

Analysis for protein targets from **Group-3** (hits 2X, opposite 1X) (See **Fig.S3D**): denoting proteins enriched when hydroxynonenal was released within the same locale in two replicates (with heavy:light ratio either > +1σ or < -1σ), but these proteins were enriched (either > 1σ or < -1σ) in the opposite locale in the remaining replicate. Parentheses indicate locale they are enriched in respective replicate.

	**Abbreviation**	**(Partial) Name in S1-1**	**(Partial) Name in S1-2**	**(Partial) Name in S1-3**
22	ANXA2	Annexin A2 (NLS)	Annexin A2 (MOMLS)	Annexin A2 (MOMLS)
23	ATP5B	ATP synthase subunit beta (NLS)	ATP synthase subunit beta (MOMLS)	ATP synthase subunit beta (MOMLS)
24	DSG1	Desmoglein-1 (NLS)	Desmoglein-1 (MOMLS)	Desmoglein-1 (MOMLS)
25	FLG2	Filaggrin-2 (NLS)	Filaggrin-2 (MOMLS)	Filaggrin-2 (MOMLS)
26	HRNR	Hornerin (NLS)	Hornerin (MOMLS)	Hornerin (MOMLS)
27	PGRMC1	Membrane-associated progesterone receptor component 2 (NLS)	Membrane-associated progesterone receptor component 2 (MOMLS)	Membrane-associated progesterone receptor component 2 (MOMLS)
28	LTA4H	Leukotriene A-4 hydrolase (NLS)	Leukotriene A-4 hydrolase (NLS)	Leukotriene A-4 hydrolase (MOMLS)
29	SERPINB3	Serpin B3 (NLS)	Serpin B3 (MOMLS)	Serpin B3 (MOMLS)
30	SERPINB12	Serpin B12 (NLS)	Serpin B12 (MOMLS)	Serpin B12 (MOMLS)
31	S100A8	Protein S100-A8 (NLS)	Protein S100-A8 (MOMLS)	Protein S100-A8 (MOMLS)
32	S100A9	Protein S100-A9 (NLS)	Protein S100-A9 (MOMLS)	Protein S100-A9 (MOMLS)

Supplementary Tables

Supplementary Table S1:

Primers used for the construction of human Halo-TeV-Flag2-ATIC, Halo-TeV-Flag2-CDK9, Halo-TeV-Flag2-DAGLB, Halo-TeV-Flag2-EPHX1, Halo-TeV-Flag2-FKBP8, Halo-TeV-Flag2-LTA4H in pFN21a vector.

Entry	Plasmid	Primers
(1)	Halo-TeV-Flag2-ATIC	Fwd-1<br>GATGACGACGATAAGGACTACAAAGACGATGACGACAAGTCAGGAATGGCTCCCGGCCAG<br>Fwd-2<br>GTACTTTCAGAGCGATAACGCGATCGCCGACTACAAGGATGACGACGATAAGGACTACAA<br>Rev-1<br>GGATCCCCGGGTACCGAGCCCGAATTCGTTTAAACGTGGTGGAAGAGCCG<br>Rev-2<br>TCGGTACCCGGGGATCCTCTAGAGTCGACCTGCAGGCATGCAAGCTGATCCGGCTGCTAA
(2)	Halo-TeV-Flag2-CDK9	Fwd-1<br>CGACGATAAGGACTACAAAGACGATGACGACAAGTCAGGAATGGCGAAGCAGTACGACTC<br>Rev-1<br>GGATCCCCGGGTACCGAGCCCGAATTCGTTTAAACGAAGACGCGCTCAA<br>Fwd-2<br>TCAGAGCGATAACGCGATCGCCGACTACAAGGATGACGACGATAAGGACTACAAAGACGA<br>Rev-2<br>TCGGTACCCGGGGATCCTCTAGAGTCGACCTGCAGGCATGCAAGCTGATCCGGCTGCTAA
(3)	Halo-TeV-Flag2-DAGLB	Fwd-1<br>GACGACGATAAGGACTACAAAGACGATGACGACAAGTCAGGAATGCCGGGGATGGTACTC

		Fwd-2<br>TTCAGAGCGATAACGCGATCGCCGACTACAAGGATGAC<br>GACGATAAGGACTACAAAGACG<br>Rev-1<br>GTCGACTCTAGAGGATCCCCGGGTACCGAGCCCGAAT<br>TCGTTTAGGCCACGTCCACACTG<br>Rev-2<br>GGGCTTTGTTAGCAGCCGGATCAGCTTGCATGCCTGCA<br>GGTCGACTCTAGAGGATCCCCG
(4)	Halo-TeV-Flag2-<br>EPHX1	Fwd-1<br>CGATAAGGACTACAAAGACGATGACGACAAGTCAGGAA<br>TGTGGCTAGAAATCCTCCTCAC<br>Fwd-2<br>AGCGATAACGCGATCGCCGACTACAAGGATGACGACG<br>ATAAGGACTACAAAGACGATGAC<br>Rev-1<br>GTCGACTCTAGAGGATCCCCGGGTACCGAGCCCGAAT<br>TCGTTTATCATTGCCGCTCCAGC<br>Rev-2<br>GGGCTTTGTTAGCAGCCGGATCAGCTTGCATGCCTGCA<br>GGTCGACTCTAGAGGATCCCCG
(5)	Halo-TeV-Flag2-<br>FKBP8	Fwd-1<br>GACGACGATAAGGACTACAAAGACGATGACGACAAGTC<br>AGGAATGGCATCGTGTGCTGAA<br>Fwd-2<br>TTCAGAGCGATAACGCGATCGCCGACTACAAGGATGAC<br>GACGATAAGGACTACAAAGACG<br>Rev-1<br>GGATCCCCGGGTACCGAGCCCGAATTCGTTTAAACGTT<br>CCTGGCAGCGA<br>Rev-2<br>GGGCTTTGTTAGCAGCCGGATCAGCTTGCATGCCTGCA<br>GGTCGACTCTAGAGGATCCCCG

(6)	Halo-TeV-Flag2-LTA4H	Fwd-1<br>GACGATAAGGACTACAAAGACGATGACGACAAGTCAGGAATGCCCGAGATAGTGGATACC<br>Fwd-2<br>TTCAGAGCGATAACGCGATCGCCGACTACAAGGATGACGACGATAAGGACTACAAAGACG<br>Rev-1<br>GATCCCCGGGTACCGAGCCCGAATTCGTTTAAACATCCACTTTTAAGTCTTTCCCC<br>Rev-2<br>GGGCTTTGTTAGCAGCCGGATCAGCTTGCATGCCTGCAGGTCGACTCTAGAGGATCCCCG
(7)	HA-CyclinT1	Fwd-1<br>CATACGACGTCCCAGACTACGCTGGAAGCTTGGTACCGATGGAGGGAGAGAGGAAGAACA<br>Fwd-2<br>TTAATACGACTCACTATAGGGCTAGCAAAGCCACCTACCCATACGACGTCCCAGACTACG<br>Rev-1<br>GGTCGACTCTAGAGGATCCCCGGGTACCGAGCCCGAATTCGTTTAGCCACACCAGCCACC<br>Rev-2<br>CGGGCTTTGTTAGCAGCCGGATCAGCTTGCATGCCTGCAGGTCGACTCTAGAGGATCCCC
(8)	Halo-TeV-NLS-FLAG2-CDK9	Fwd-1<br>CGACGATAAGGACTATAAAGATGACGACGATAAGTCAGGAATGGCGAAGCAGTACGACTC<br>Fwd-2<br>AAGAAGAAGAGGAAGGTGGATTACAAGGATGACGACGATAAGGACTATAAAGATGACGAC<br>Rev-1<br>CTTTCAGAGCGATAACGCGATCGCCTCAGGACCCAAGAAGAAGAGGAAGGTGGATTACAA

		Rev-2<br>CGCTCGAGATTTCCGGCGAGCCAACCACTGAGGATCT<br>GTACTTTCAGAGCGATAACGCGA
(9)	Halo-TeV-NES-<br>Flag2-CDK9	Fwd-1<br>CGACGATAAGGACTATAAAGATGACGACGATAAGTCAG<br>GAATGGCGAAGCAGTACGACTC<br>Fwd-2<br>CTCGCCGGGCTAGACCTGGATTACAAGGATGACGACG<br>ATAAGGACTATAAAGATGACGAC<br>Rev-1<br>AGAGCGATAACGCGATCGCCTCAGGAGAGGAACTCGC<br>ACTCAAACTCGCCGGGCTAGACC<br>Rev-2<br>TCGAGATTTCCGGCGAGCCAACCACTGAGGATCTGTAC<br>TTTCAGAGCGATAACGCGATCG
(10)	HA-HEXIM1	Fwd-1<br>TACCCATACGACGTCCCAGACTACGCTGGAAGCTTGGT<br>ACCGATGGCCGAGCCATTCTTG<br>Fwd-2<br>GAGTATTAATACGACTCACTATAGGGCTAGCAAAGCCA<br>CCTACCCATACGACGTCCCAGA<br>Rev-1<br>AGCAGCCAACTCAGCTTCCTTTCGGGCTTTGTTAGCAG<br>CCGGATCAGCTTGCATGCCTGC<br>Rev-2<br>GCGGCCGCCCCAAGGGGTTATGCTAGTTATTGCTCAGC<br>GGTGGCAGCAGCCAACTCAGCT
(11)	HA-HEXIM1<br>Y203D mutant	Fwd-1<br>GTTCGCCAAGGGCCAGCCGGTCGCGCCCGATAACACC<br>ACGCAGTTCCTCATGGATG<br>Rev-1<br>CATCCATGAGGAACTGCGTGGTGTTATCGGGCGCGAC<br>CGGCTGGCCCTTGGCGAAC

(12)	Halo-TeV-Flag2-CDK9 C95A mutant	Fwd-1<br>AGGGTAGTATATACCTGGTGTTCGACTTCTGCGAGCATGACCTTGCTGGGC<br>Rev-1<br>GCCCAGCAAGGTCATGCTCGCAGAAGTCGAACACCAGGTATATACTACCCT
(13)	Halo-TeV-Flag2-CDK9 D106A Halo dead mutant	Fwd-1<br>CTGGAAGAGGTCGTCCTGGTCATTCACGCTTGGGGCTCCGCTCTGGGTTTCCACTGGGC<br>Rev-1<br>GACCTTCTCCAGCAGGACCAGTAAGTGCGAACCCCGAGGCGAGACCCAAAGGTGACCCG
(14)	HA-CDK9	Fwd-1<br>ACCCATACGACGTCCCAGACTACGCTGGAAGCTTGGTACCGATGGCGAAGCAGTACGACT<br>Rev-1<br>CCAACTCAGCTTCCTTTCGGGCTTTGTTAGCAGCCGGATTAAACGAAGACGCGCTCAAAC<br>Fwd-2<br>TATTAATACGACTCACTATAGGGCTAGCAAAGCCACCATGTACCCATACGACGTCCCAGA<br>Rev-2<br>CCAAGGGGTTATGCTAGTTATTGCTCAGCGGTGGCAGCAGCCAACTCAGCTTCCTTTCGG
(15)	NES-HA-CDK9	Fwd-2<br>GGAACTCGCACTCAAACTCGCCGGGCTAGACCTGAGTGGTTACCCATACGACGTCCCAGA<br>Rev-2<br>CCAAGGGGTTATGCTAGTTATTGCTCAGCGGTGGCAGCAGCCAACTCAGCTTCCTTTCGG<br>Fwd-3<br>TTAATACGACTCACTATAGGGCTAGCAAAGCCACCATGGAGGAACTCGCACTCAAACTCG

		Rev-3<br>AATGTATCTTATCATGTCTGCTCGAAGCGGCCGCCCCA<br>AGGGGTTATGCTAGTTATTGCT
(16)	NLS-HA-CDK9	Fwd-2<br>TCTTAAGGCTAGAGTATTAATACGACTCACTATAGGGCT<br>AGCAAAGCCACCATGCCCAAG<br>Rev-2<br>AATGTATCTTATCATGTCTGCTCGAAGCGGCCGCCCCA<br>AGGGGTTATGCTAGTTATTGCT
(17)	Flag2-CDK9(wt) or Flag2-CDK9(C95A)	Fwd-1<br>CGACGATAAGGACTACAAAGACGATGACGACAAGTCAG<br>GAATGGCGAAGCAGTACGACTC<br>Rev-1<br>TTAGAAGACGCGCTCAAACTCCG<br>Fwd-2<br>CCTGGTGCCTCGTGGTAGCCATGACTACAAGGATGACG<br>ACGATAAGGACTACAAAGAC<br>Rev-2<br>TTAGAAGACGCGCTCAAACTCCG<br>Fwd-3<br>ACCATGGGCAGCAGCCATCATCATCATCATCACAGCAG<br>CGGCCTGGTGCCTCGTGGTAGC<br>Rev-3<br>TTAGAAGACGCGCTCAAACTCCG<br>Fwd-4<br>TTTCAGGTGTCGTGAGGAATTTCGACATTTCCACCATG<br>GGCAGCAGCC<br>Rev-4<br>TAACCGATACCGTCGAGATTAATTAAATTTTTAGAAGAC<br>GCGCTCAAACTC<br>Fwd-5<br>CAGACAGTGGTTCAAAGTTTTTTTCTTCCATTTCAGGTG<br>TCGTGAGGAATTTC

		Rev-5<br>CCCCCCAATCCCCCCTTTTCTTTTAAAAGTTAACCGATA<br>CCGTCGAGATTAA<br>Fwd-6<br>GAACAGCCAGCCCAACCGCTACACAAATCGAGTTGTGA<br>CACTCTGGTACCGGCCCCCGGA<br>Rev-6<br>GATTAATTAAATTTTTAGAAGACGCGC

Supplementary Table S2: shRNA sequences

The following shRNA sequences were used in pLKO1 vector (from Sigma MISSION shRNA clone library). For details, see lentivirus production and infection method sections.

Entry	Plasmid	Hairpin sequences
(1)	shCyclinT1-35 (TRCN0000273835)	CCGGGCCAATGTGAAGTCACAATATCTCGAGATATTGTGACTTCACATTGGCTTTTTG
(2)	shCyclinT1-36 (TRCN0000273836)	CCGGAGATTGCCAAGAGTACTAAATCTCGAGATTTAGTACTCTTGGCAATCTTTTTTG
(3)	shHEXIM1-62 (TRCN0000245062)	CCGGGCGGCATTGGAAACCGTACTACTCGAGTAGTACGGTTTCCAATGCCGCTTTTTG
(4)	shHEXIM1-63 (TRCN0000245063)	CCGGACACCAGCGATGACGACTTCACTCGAGTGAAGTCGTCATCGCTGGTGTTTTTTG
(5)	shHEXIM1-74 (TRCN0000074174)	CCGGGCATTGGAAACCGTACTACAACTCGAGTTGTAGTACGGTTTCCAATGCTTTTTG
(6)	shLac Z (shRNA control)-D11 (TRCN0000231722)	CGCGATCGTAATCACCCGAGT
(7)	shCDK9-92 (TRCN0000199892)	CCGGGTTCGACTTCTGCGAGCATGACTCGAGTCATGCTCGCAGAAGTCGAACTTTTTTG
(8)	shCDK9-94 (TRCN0000000494)	CCGGGCACAGTTTGGTCCGTTAGAACTCGAGTTCTAACGGACCAAACTGTGCTTTTT
(9)	shCDK9-97 (TRCN0000000497)	CCGGCTACTACATCCACAGAAACAACTCGAGTTGTTTCTGTGGATGTAGTAGTTTTT
(10)	shCyclinT1-28 (TRCN0000273837)	CCGGTTAGGCTTTGAACTAACAATTCTCGAGAATTGTTAGTTCAAAGCCTAATTTTTG
(11)	shCyclinT1-29 (TRCN0000013673)	CCGGGCCTTGTTTCTAGCAGCTAAACTCGAGTTTAGCTGCTAGAAACAAGGCTTTTT
(12)	shCyclinT1-17 (TRCN0000013677)	CCGGCGGTGGTATTTCACTCGAGAACTCGAGTTCTCGAGTGAAATACCACCGTTTT

Supplementary Table S3: siRNA sequences (from GE horizon dharmacon siRNA collection)

Entry	Plasmid	Company	Sequences
(1)	siCyclinT1-1	GE dharmacon	UAGUAUACAUGCAUCGAUU
(2)	siCyclinT1-2	GE dharmacon	GAACAAACGUCCUGGUGAU
(3)	siCyclinT1-3	GE dharmacon	UAUCAACACUGCUAUAGUA
(4)	siCDK9	Santa Cruz (sc-29268)	Sequence not released by supplier (Pooled siRNAs targeting human CDK9)
(5)	siCont	Santa Cruz (sc-44233)	Sequence not released by supplier (Scrambled sequence: siRNA control)

Supplementary Table S4: gRNA sequence

The following gRNA sequence was used in the lentiCRISPR v2 vector (Addgene #52961).

For details, see lentivirus production and infection method sections.

**Entry**	**Plasmid**	**Sequences**
(1)	gRNA 1	CAACCGCTACACCAACCGTG
(2)	gRNA Control	CTTCGAAATGTCCGTTCGGT

Supplementary Table S5: Summary of antibodies

Antibody	Application	Catalog number; Supplier	Dilution
Mouse monoclonal anti-β-Actin-HRP	WB	A4700, Sigma Aldrich	1:30000
Rabbit polyclonal anti-Halo	WB	G9281, Promega	1:1000
Monoclonal anti-gapdh-peroxidase	WB	G92296, Sigma	1:30000
Mouse monoclonal anti-FLAG M2	WB<br>IF	F3165, Sigma	1:3000<br>1:300
Rabbit polyclonal anti-FLAG	WB<br>IF	PA1-984B, Fisher Pierce	1:3000<br>1:300
Goat polyclonal anti-FLAG	IF	Ab1257, Abcam	1:300
Rat monoclonal anti-HA HRP, 3F10	WB<br>IF	11867423001, Sigma Aldrich	1: 3000<br>1: 300
Secondary antibody to rabbit IgG, HRP linked	WB	7074; Cell Signaling Technology	1:5000
Secondary antibody to mouse, HRP linked	WB	7076, Cell signaling Technology	1:5000
Donkey Anti-rabbit IgG AlexaFluor® 647	IF	Ab150075; Abcam	1:1000
Goat Anti-Rat IgG H&L AlexaFluor® 568	IF	Ab175710; Abcam	1:1000
Goat Anti-rabbit AlexaFluor® 488	IF	A11008; Invitrogen	1:1000
Goat Anti-Mouse Ig, Human ads-FITC	IF	1010-02, Southern Biotech	1:1000
Anti-RNA polymerase II CTD repeat YSPTSPS (phospho S2) antibody	WB<br>IF	Ab5095, Abcam	1: 3000<br>1: 300
Anti-RNA polymerase II CTD repeat YSPTSPS (phospho S5) antibody	WB<br>IF	Ab5131, Abcam	1: 3000<br>1: 300
Pol II Antibody (CTD4H8), RNAPII CTD pan-phospho antibody	WB	sc-47701, Santa Cruz Bio	1:300
Pol II Antibody (8WG16), RNAPII full-length antibody	WB	sc-56767, Santa Cruz Bio	1:300
Anti-Cdk9 antibody	WB<br>IF	ab76320, Abcam	1:3000<br>1:300
Anti-Cdk9 antibody	WB	11705-1-AP, ProteinTech	1:3000
Anti-CyclinT1 antibody	WB<br>IF	ab184703, Abcam	1:3000<br>1:300
Anti-HEXIM1 antibody	WB<br>IF	Ab25388, Abcam	1:3000<br>1:300
Anti-HEXIM1 antibody	WB	Ab28016, Abcam	1:3000
Anti-Lamin B antibody (B-10)	WB	sc-374015, Santa Cruz	1:300
Anti-4-HNE antibody	IP	ab46544, Abcam	1:45
Anti-Cdk9 antibody	IP	11705-1-AP, Proteintech	1:40
Goat IgG isotype control	IP	31245, ThermoFisher	1:660
Normal Rabbit IgG	IP	2729, Cell signaling Technology	1:60
Anti-Cdk9 Antibody (D-7)	IF	sc-13130, Santa Cruz Bio	1:500

Supplementary Table S6: Chemical structures of hydroxynonenal(HNE)-derived adducts at Cysteine, Lysine, and Histidine residues with their corresponding mass shifts (rounded to 5 decimal points) searched in MS analysis. Also see **Supplementary Table S7-S10**.

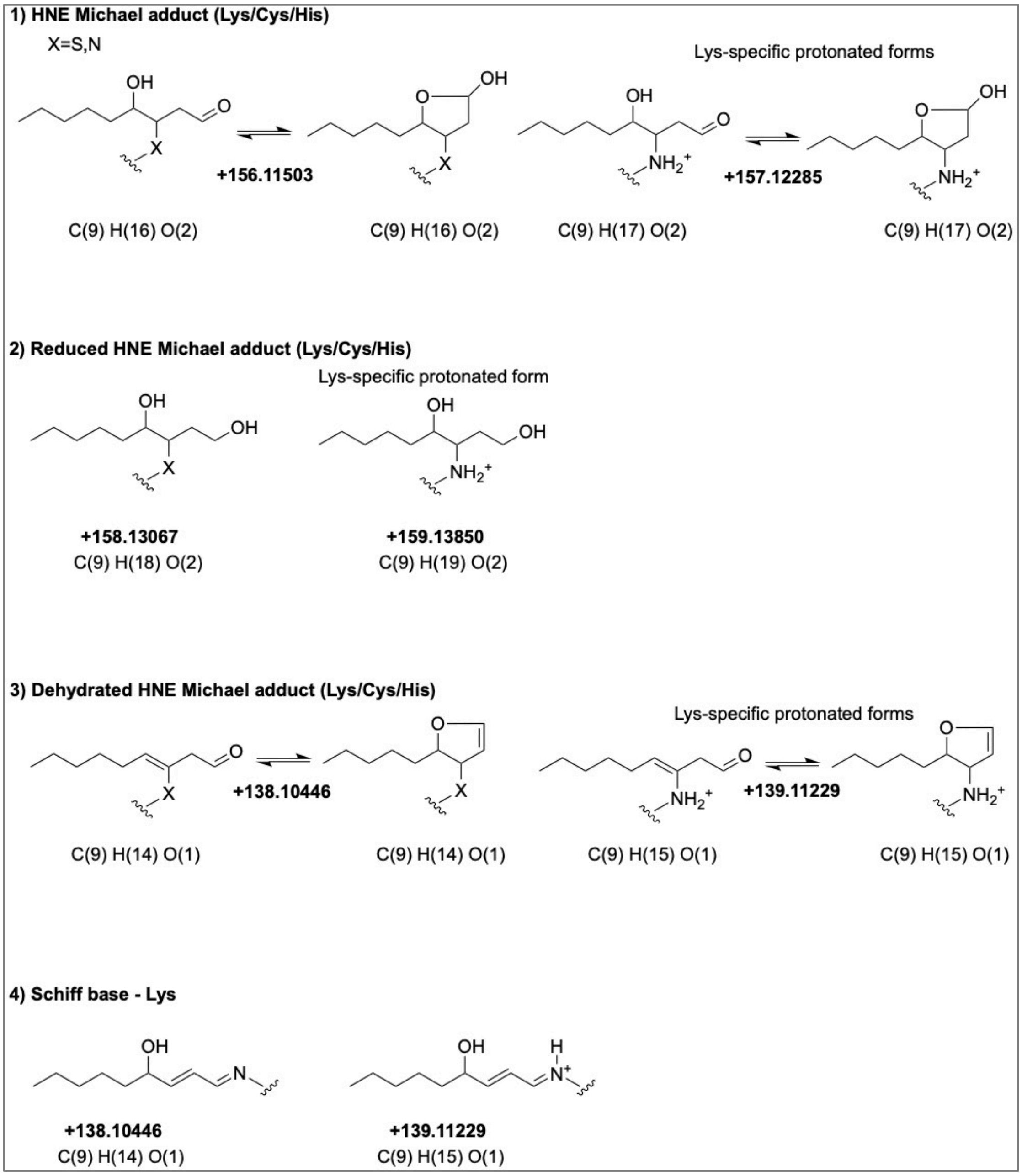

Supplementary Table S7: LC-MS/MS-based identification of HNE modification on CDK9 immunoprecipitated from untreated HEK293T cells.

Human CDK9 isoform 1 (100%), 42.7 kDa, Mascot Score 1426, 9 unique peptides with different modifications, 1 distinct peptide with added mass of 156.11503 Da for HNE Michael adduct (see **Supplementary Table S6**). 105/372 amino acids (28% coverage). Matched peptide with HNE-related modifications is marked in green, other matched peptides are shown in red. Protease: Asp-N+Arg-C

MAKQYDSVEC PFCDEVSKYE KLAKIGQGTF GEVFKARHRK TGQKVALKKV LMENEKEGFP ITALREIKIL QLLKHENVVN LIEICRTKAS PYNRCKGSIY LVFDFCEHDL AGLLSNVLVK FTLSEIKRVM QMLLNGLYYI HRNKILHRDM KAANVLITRD GVLKLADFGL ARAFSLAKNS QPNRYTNRVV TLWYRPPELL LGERDYGPPI DLWGAGCIMA EMWTRSPIMQ GNTEQHQLAL ISQLCGSITP EVWPNVDNYE LYEKLELVKG QKRKVKDRLK AYVRDPYALD LIDKLLVLDP AQRIDSDDAL NHDFFWSDPM PSDLKGMLST HLTSMFEYLA PPRRKGSQIT QQSTNQSRNP ATTNQTEFER VF

Unique peptide with modification (asterisk indicates modified residue)

<u>K345</u>
K*GSQITQQSTNQSR.

Charge: +3. Monoisotopic m/z: 573.63546 Da (-3.78 mmu/-6.6 ppm). MH+: 1718.89181 Da. Retention time: 28.8494 min.

Identified with: Mascot (v1.36). Ions Score: 57. p-value: 2e-6. Percolator q-Value: 1.4e-3. Percolator PEP: 2.8e-2. Ions matched by search engine: 7/156.

Modification: K1(HNE Michael adduct). Fragment match tolerance used for search: 0.05 Da. Expectation value: 1.2e-4.

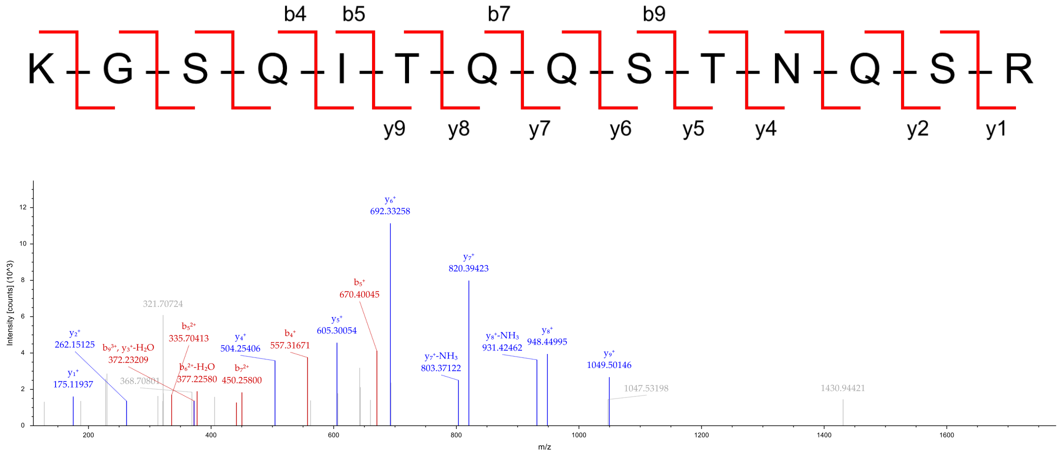

Supplementary Table S8: LC-MS/MS-based identification of HNE modification on CDK9 immunoprecipitated from HEK293T cells treated with 113 μM HNE for 2 h.

Human CDK9 isoform 1 (100%), 42.7 kDa, Mascot Score 753, 37 unique peptides with different modifications, 2 distinct peptides with added mass of 156.11503 Da for HNE Michael adduct (see **Supplementary Table S6**). 196/372 amino acids (52% coverage). Matched peptide with HNE-related modifications is marked in green, other matched peptides are shown in red. Protease: Asp-N+Arg-C<br><br>MAKQYDSVEC PFCDEVSKYE KLAKIGQGTF GEVFKARHRK TGQKVALKKV LMENEKEGFP ITALREIKIL QLLKHENVVN LIEICRTKAS PYNRCKGSIY LVFDFCEHDL AGLLSNVLVK FTLSEIKRVM QMLLNGLYYI HRNKILHRDM KAANVLITRD GVLKLADFGL ARAFSLAKNS QPNRYTNRVV TLWYRPPELL LGERDYGPPI DLWGAGCIMA EMWTRSPIMQ GNTEQHQLAL ISQLCGSITP EVWPNVDNYE LYEKLELVKG QKRKVKDRLK AYVRDPYALD LIDKLLVLDP AQRIDSDDAL NHDFFWSDPM PSDLKGMLST HLTSMFEYLA PPRRKGSQIT QQSTNQSRNP ATTNQTEFER VF
**Unique peptide with modification (asterisk indicates modified residue)**<br><br>**<u>K345</u>**<br>**K*GSQITQQSTNQSR.**<br><br>Charge: +3. Monoisotopic m/z: 573.63422 Da (-5.03 mmu/-8.76 ppm). MH+: 1718.88809 Da, Retention time: 26.7550 min.<br><br>Identified with: Mascot (v1.36). Ions Score: 61. p-value: 7.9e-7. Percolator q-Value: 1.9e-3. Percolator PEP: 1.3e-2. Ions matched by search engine: 7/156.<br><br>Modification: K1(HNE Michael adduct). Fragment match tolerance used for search: 0.05 Da. Expectation value: 9.5e-6.<br><br>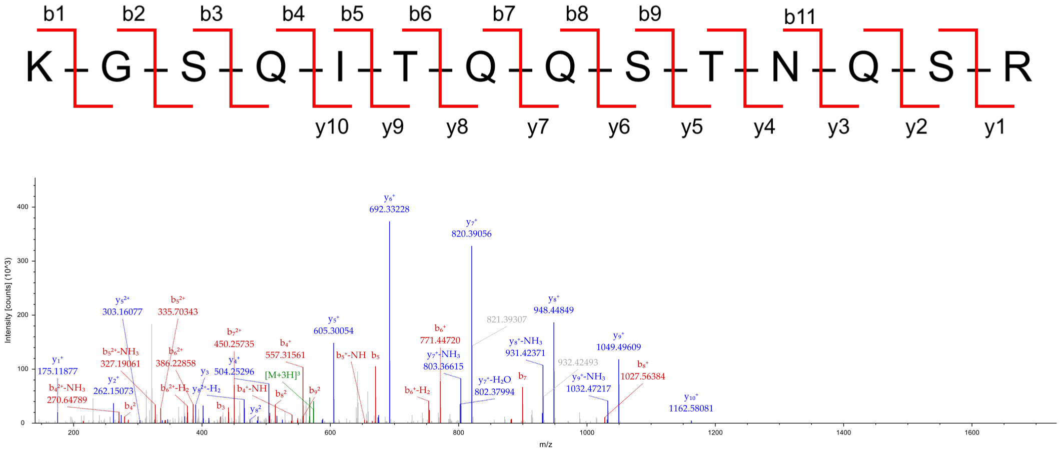

Supplementary Table S9: LC-MS/MS-based identification of HNE modification on CDK9 immunorpecipitated from untreated HeLa cells.

Human CDK9 isoform 1 (100%), 42.7 kDa, Mascot Score 1332, 10 unique peptides with different modifications, 1 distinct peptides with added mass of 156.11503 Da for HNE Michael adduct (see **Supplementary Table S6**). 88/372 amino acids (24% coverage). Matched peptide with HNE-related modifications is marked in green, other matched peptides are shown in red. Protease: Asp-N+Arg-C

MAKQYDSVEC PFCDEVSKYE KLAKIGQGTF GEVFKARHRK TGQKVALKKV LMENEKEGFP ITALREIKIL QLLKHENVVN LIEICRTKAS PYNRCKGSIY LVFDFCEHDL AGLLSNVLVK FTLSEIKRVM QMLLNGLYYI HRNKILHRDM KAANVLITRD GVLKLADFGL ARAFSLAKNS QPNRYTNRVV TLWYRPPELL LGERDYGPPI DLWGAGCIMA EMWTRSPIMQ GNTEQHQLAL ISQLCGSITP EVWPNVDNYE LYEKLELVKG QKRKVKDRLK AYVRDPYALD LIDKLLVLDP AQRIDSDDAL NHDFFWSDPM PSDLKGMLST HLTSMFEYLA PPRRKGSQIT QQSTNQSRNP ATTNQTEFER VF

Unique peptide with modification (asterisk indicates modified residue)

K345
K*GSQITQQSTNQSR.

Charge: +3. Monoisotopic m/z: 573.63537 Da (-3.87 mmu/-6.75 ppm). MH+: 1718.89155 Da. Retention time: 28.9315 min.

Identified with: Mascot (v1.36). Ions Score: 68. p-value: 1.5e-7. Percolator q-Value: 2.1e-3. Percolator PEP: 7.9e-3. Ions matched by search engine: 8/156.

Modification: K1(HNE Michael adduct). Fragment match tolerance used for search: 0.05 Da. Expectation value: 2.4e-6.

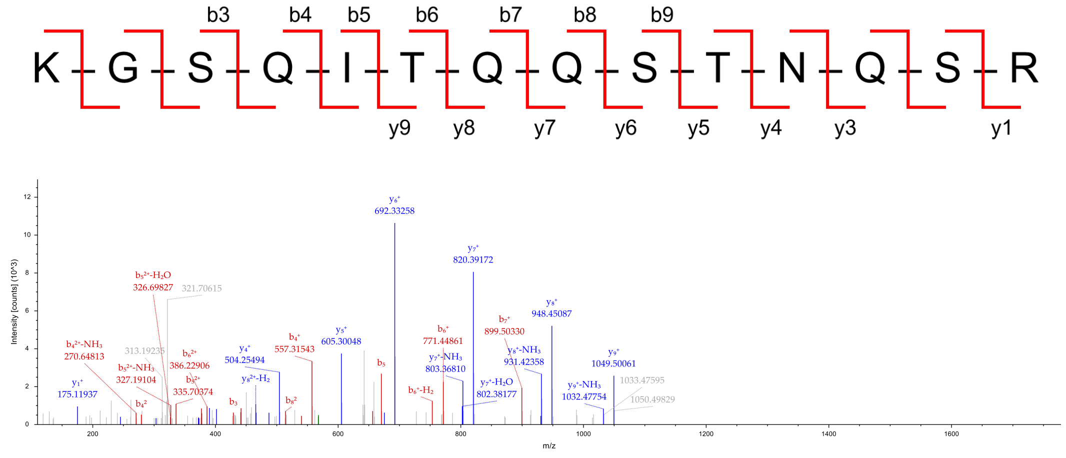

Supplementary Table S10: LC-MS/MS-based identification of HNE modification on CDK9 immunoprecipitated from HeLa cells treated with 113 μM HNE for 2 h.

Human CDK9 isoform 1 (100%), 42.7 kDa, Mascot Score 506, 26 unique peptides with different modifications, 2 distinct peptides with added mass of 156.11503 Da for HNE Michael adduct (see **Supplementary Table S6**). 133/372 amino acids (35% coverage). Matched peptide with HNE-related modifications is marked in green, other matched peptides are shown in red. Protease: Asp-N+Arg-C

MAKQYDSVEC PFCDEVSKYE KLAKIGQGTF GEVFKARHRK TGQKVALKKV LMENEKEGFP ITALREIKIL QLLKHENVVN LIEICRTKAS PYNRCKGSIY LVFDFCEHDL AGLLSNVLVK FTLSEIKRVM QMLLNGLYYI HRNKILHRDM KAANVLITRD GVLKLADFGL ARAFSLAKNS QPNRYTNRVV TLWYRPPELL LGERDYGPPI DLWGAGCIMA EMWTRSPIMQ GNTEQHQLAL ISQLCGSITP EVWPNVDNYE LYEKLELVKG QKRKVKDRLK AYVRDPYALD LIDKLLVLDP AQRIDSDDAL NHDFFWSDPM PSDLKGMLST HLTSMFEYLA PPRRKGSQIT QQSTNQSRNP ATTNQTEFER VF

Unique peptide with modification (asterisk indicates modified residue)

<u>K345</u>
K*GSQITQQSTNQSR.

Charge: +3. Monoisotopic m/z: 573.63465 Da (-4.59 mmu/-8 ppm). MH+: 1718.88941 Da. Retention time: 26.9812 min.

Identified with: Mascot (v1.36). Ions Score: 65. p-value: 3.1e-7. Percolator q-Value: 1.2e-3. Percolator PEP: 5.6e-3. Ions matched by search engine: 9/156.

Modification: K1(HNE Michael adduct). Fragment match tolerance used for search: 0.05 Da. Expectation value: 4.2e-6.

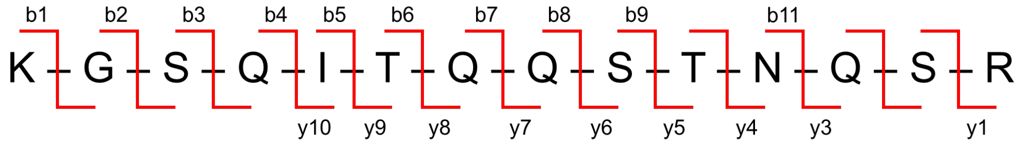

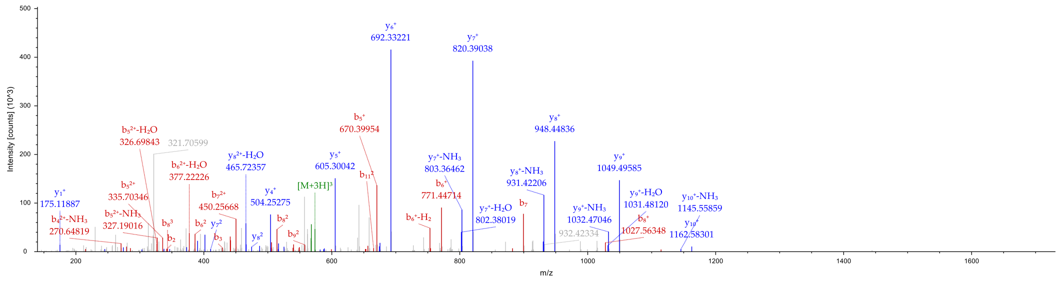

Supplementary Table S11: Summary of RT-qPCR primers

**Entry**	**Plasmid**	**Sequences**
(1)	GAPDH Fwd	GACAGTCAGCCGCATCTTCT
(2)	GAPDH Rev	AAATGAGCCCCAGCCTTCTC
(3)	MYC Fwd	CGTCCTCGGATTCTCTGCTC
(4)	MYC Rev	GCTGCGTAGTTGTGCTGATG
(5)	CCND Fwd	CTGATTGGACAGGCATGGGT
(6)	CCND Rev	GTGCCTGGAAGTCAACGGTA
(7)	CCNE Fwd	GGGGATCAGTCCTTGCATTA
(8)	CCNE Rev	TCAGGCAAAGGTGAAGGATT
(9)	RNR-β Fwd	AGGAGGTGGACCTCTCCAAG
(10)	RNR-β Rev	TCTTTGTCCCCAATCCAGCG
(11)	Nrf2 Fwd	TATCCATTCCTGAGTTACAGTGTC
(12)	Nef2 Rev	CTGTCAGTTTGGCTTCTGGAC

Supplemental References

1. Parvez, S. *et al.* T-REX on-demand redox targeting in live cells. *Nature protocols* **11**, 2328 (2016).
2. Long, M.J. *et al.* β-TrCP1 is a vacillatory regulator of Wnt signaling. *Cell chemical biology* **24**, 944-957. e947 (2017).
3. Long, M.J.C. *et al.* Akt3 is a privileged first responder in isozyme-specific electrophile response. *Nature Chemical Biology* **13**, 333-338 (2017).
4. Van Hall‐Beauvais, A., Zhao, Y., Urul, D.A., Long, M.J. & Aye, Y. Single‐Protein‐Specific Redox Targeting in Live Mammalian Cells and C. elegans. *Current protocols in chemical biology* **10**, e43 (2018).
5. Lin, H.-Y., Haegele, J.A., Disare, M.T., Lin, Q. & Aye, Y. A generalizable platform for interrogating target-and signal-specific consequences of electrophilic modifications in redox-dependent cell signaling. *Journal of the American Chemical Society* **137**, 6232-6244 (2015).
6. Emmott, E. & Goodfellow, I. Identification of protein interaction partners in mammalian cells using SILAC-immunoprecipitation quantitative proteomics. *JoVE (Journal of Visualized Experiments)*, e51656 (2014).
7. Zhao, Y., Long, M.J., Wang, Y., Zhang, S. & Aye, Y. Ube2V2 is a Rosetta stone bridging redox and ubiquitin codes, coordinating DNA damage responses. *ACS central science* **4**, 246-259 (2018).
8. Yang, Y., Anderson, E. & Zhang, S. Evaluation of six sample preparation procedures for qualitative and quantitative proteomics analysis of milk fat globule membrane. *Electrophoresis* **39**, 2332-2339 (2018).
9. Harman, R.M., He, M.K., Zhang, S. & Van de Walle, G.R. Plasminogen activator inhibitor-1 and tenascin-C secreted by equine mesenchymal stromal cells stimulate dermal fibroblast migration in vitro and contribute to wound healing in vivo. *Cytotherapy* **20**, 1061-1076 (2018).
10. Rappsilber, J., Mann, M. & Ishihama, Y. Protocol for micro-purification, enrichment, pre-fractionation and storage of peptides for proteomics using StageTips. *Nature Protocols* **2**, 1896-1906 (2007).
11. Parvez, S. *et al.* Substoichiometric hydroxynonenylation of a single protein recapitulates whole-cell-stimulated antioxidant response. *J Am Chem Soc* **137**, 10-13 (2015).
12. Fu, Y. *et al.* Nuclear RNR-α antagonizes cell proliferation by directly inhibiting ZRANB3. *Nature Chemical Biology* **14**, 943-954 (2018).
